# Compounds targeting OSBPL7 increase ABCA1-dependent cholesterol efflux preserving kidney function in two models of kidney disease

**DOI:** 10.1038/s41467-021-24890-3

**Published:** 2021-08-02

**Authors:** Matthew B. Wright, Javier Varona Santos, Christian Kemmer, Cyrille Maugeais, Jean-Philippe Carralot, Stephan Roever, Judith Molina, G. Michelle Ducasa, Alla Mitrofanova, Alexis Sloan, Anis Ahmad, Christopher Pedigo, Mengyuan Ge, Jeffrey Pressly, Laura Barisoni, Armando Mendez, Jacopo Sgrignani, Andrea Cavalli, Sandra Merscher, Marco Prunotto, Alessia Fornoni

**Affiliations:** 1 Pharma Research and Early Development (pRED), Roche Innovation Center Basel, F. Hoffmann-La Roche Ltd, Basel, Switzerland; 2 Katz Family Division of Nephrology and Hypertension, Department of Medicine, University of Miami, Miller School of Medicine, Miami, FL USA; 3 Peggy and Harold Katz Family Drug Discovery Center, University of Miami, Miller School of Medicine, Miami, FL USA; 4 Department of Molecular and Cellular Pharmacology, University of Miami Miller School of Medicine, Miami, FL USA; 5 Sylvester Comprehensive Cancer Center, University of Miami, Miller School of Medicine, Miami, FL USA; 6 Department of Pathology, University of Miami, Miller School of Medicine, Miami, FL USA; 7 Diabetes Research Institute, University of Miami, Miller School of Medicine, Miami, FL USA; 8 Institute for Research in Biomedicine, Università della Svizzera Italiana, Bellinzona, Switzerland; 9 Swiss Institute of Bioinformatics, Lausanne, Switzerland; 10 School of Pharmaceutical Sciences, University of Geneva, Geneva, Switzerland

**Keywords:** Drug discovery and development, Alport syndrome, Kidney diseases, Lipids

## Abstract

Impaired cellular cholesterol efflux is a key factor in the progression of renal, cardiovascular, and autoimmune diseases. Here we describe a class of 5-arylnicotinamide compounds, identified through phenotypic drug discovery, that upregulate ABCA1-dependent cholesterol efflux by targeting Oxysterol Binding Protein Like 7 (OSBPL7). OSBPL7 was identified as the molecular target of these compounds through a chemical biology approach, employing a photoactivatable 5-arylnicotinamide derivative in a cellular cross-linking/immunoprecipitation assay. Further evaluation of two compounds (Cpd A and Cpd G) showed that they induced ABCA1 and cholesterol efflux from podocytes in vitro and normalized proteinuria and prevented renal function decline in mouse models of proteinuric kidney disease: Adriamycin-induced nephropathy and Alport Syndrome. In conclusion, we show that small molecule drugs targeting OSBPL7 reveal an alternative mechanism to upregulate ABCA1, and may represent a promising new therapeutic strategy for the treatment of renal diseases and other disorders of cellular cholesterol homeostasis.

## Abstract (web summary)

This study describes a class of small molecule compounds that promote ABCA1-dependent cholesterol efflux via a non-transcriptional mechanism, the identification of the molecular target by a chemical biology approach, and the potential of these agents for the treatment of chronic kidney diseases and potentially other diseases where lipid accumulation drives disease progression.

## Introduction

Chronic kidney disease (CKD) is a major health burden in the United States affecting more than 30 million people^1^. Substantial progress to slow the progression of CKD has been made through the introduction of angiotensin converting enzyme inhibitors (ACEi) and angiotensin receptor blockers (ARBs) as well as sodium-glucose cotransporter-type 2 inhibitors (SGLT2i). However, many patients with CKD still progress to end stage renal disease (ESRD)^2–6^. Substantial research efforts are ongoing to improve the treatment of CKD. The identification of new disease-specific targets, and new drugs allowing early intervention in prevalent (e.g., diabetic kidney disease), as well as less common renal diseases (e.g., focal segmental glomerulosclerosis and Alport syndrome), are desired.

We and others have shown that glomerular accumulation of lipids, e.g., cholesterol, occurs in diabetic kidney disease (DKD)^7–11^, focal segmental glomerulosclerosis (FSGS)^12–14^, and Alport syndrome (AS)^13^ and is associated with downregulation of ATP-binding cassette transporter (ABCA1)-mediated cholesterol efflux. We have also shown that genetic overexpression of ABCA1 rescues mice from FSGS-type glomerulosclerosis^14^, and that podocyte-specific ABCA1 deficiency worsens experimental DKD^7^. We recently reported that depleting glomeruli of cholesterol with cyclodextrin is also renoprotective in models of DKD^9^, FSGS^13,14^, and AS^13^. Though protective, cyclodextrin promotes cholesterol removal in a rather unselective way, and its route of administration (i.e., parenteral) is inconvenient. Together, these data suggest alternative approaches to upregulate ABCA1-mediated cholesterol efflux may offer effective treatments for FSGS and other renal diseases.

Prior compounds, such as T1317 and GW3965, agonists of the liver-X-receptors (LXR), were shown to significantly induce ABCA1 mRNA expression and promote cellular cholesterol efflux to lipoproteins^15–17^. In renal disease, LXR agonists improve cellular cholesterol transport, and blunt inflammatory responses in macrophages exposed to lipoproteins from patients with moderate to severe CKD^18^. In diabetic mice, LXR agonists decrease proteinuria, renal inflammation, and renal glomerular cholesterol content by increasing ABCA1^19–21^. Unfortunately, LXR agonists have the undesired effect to increase plasma and liver triglycerides which has frustrated clinical development^22^.

In this work, we describe the results of a phenotypic drug discovery (PDD) initiative that led to the identification of a class of small molecule inducers of ABCA1 (5-arylnicotinamides). Target deconvolution, using chemical biology, led to the identification of oxysterol binding protein like 7 (OSBPL7) as the molecular target. Homology modeling and ligand docking reveal that the compounds interact directly with the predicted sterol binding pocket. Further optimization led to the identification of compounds A (Cpd A) and G (Cpd G) that are suitable for in vivo testing. Both Cpd A and Cpd G increase plasma membrane ABCA1 in cultured human podocytes, and significantly increase ABCA1-dependent cholesterol efflux. Efficacy testing in mouse models of proteinuric kidney disease (adriamycin-induced nephropathy and Alport syndrome) revealed that Cpds A and G normalize proteinuria, and significantly reduce renal fibrosis and renal functional decline. These results show that small molecule drugs that target OSBPL7 offer an alternative approach to upregulate ABCA1 activity, and may represent an effective, safe new therapeutic strategy for the treatment of renal diseases.

## Results

### Discovery of the 5-arylnicotinamides as inducers of ABCA1

**Fig. 1:**
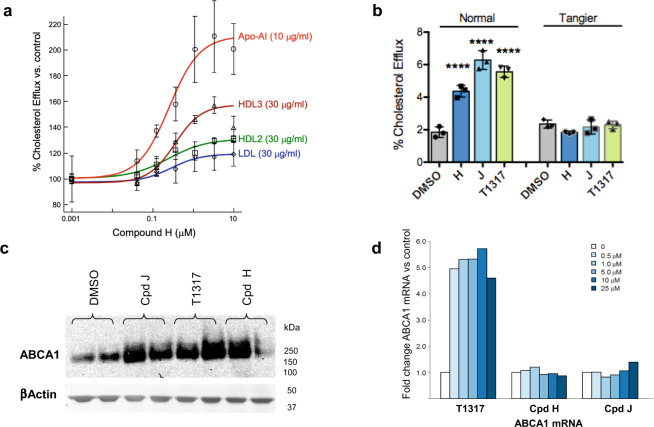
Effects of 5-arylnicotinamides on ABCA1-mediated cholesterol efflux. **a** Concentration-response of Cpd H on cholesterol efflux from human THP1 macrophages to different extracellular apolipoprotein acceptors. Data are represented as the mean ± SD from three independent experiments. **b** Cholesterol efflux assays were performed in normal human or Tangier (ABCA1-deficient) fibroblasts. Left panel, in normal fibroblasts both Cpd H and Cpd J (10 μM) increased cholesterol efflux to apoAI. Right panel, none of the test agents had an effect on cholesterol efflux to apoAI or other lipoprotein acceptors in fibroblasts genetically deficient for ABCA1. Data expressed as the mean ± SD from three independent experiments. Groups were compared to DMSO control using One-way ANOVA, (F(7, 16) = 76.4), followed by Dunnett’s test. *P* < 0.0001 (****) when compounds were used in normal fibroblasts. *P* > 0.05 when compounds were used in Tangier fibroblasts: Cpd H, *P* = 0.4; Cpd J, *P* > 0.99; T1317, *P* = 0.5. **c** Western blot for ABCA1 protein from cholesterol-loaded THP1 macrophages treated with the LXR agonist T1317 or the two ABCA1 inducers, Cpd J and Cpd H. Image representative from three independent experiments. Duplicate protein samples from separate wells were loaded for each treatment. β-Actin was used as a loading control. **d** Relative mRNA expression level of ABCA1 mRNA determined by quantitative real-time PCR after treatment of cholesterol-loaded THP1 macrophages for 24 h with the indicated compounds and concentrations.

We performed a phenotypic screen using cholesterol-loaded human macrophages (THP1 cells) to identify compounds able to stimulate cholesterol efflux to extracellular apoAI. We identified a new lead series, represented by the 5-arylnicotinamide compound H (Cpd H). Cpd H increased efflux of radiolabeled cholesterol most effectively to lipid-poor HDL (HDL3) or to apoAI, suggesting that the effect is mediated by ABCA1 (Fig. 1a). Cpd H originated from a prior drug discovery program for cannabinoid receptor type 1 (CB1R) antagonists for the treatment of metabolic disorders^23^. Its structure and potency for binding to CB1R are shown in Supplementary Fig. 1 and Supplementary Table 1.

Efflux experiments were performed in fibroblasts from an individual with Tangier disease^24^, a syndrome caused by genetic loss of ABCA1. Both the 5-arylnicotinamides, Cpd H and Cpd J, and the LXR agonist T1317, were able to increase cholesterol efflux to apoAI from normal, but not from ABCA1-deficient fibroblasts, demonstrating the absolute requirement for ABCA1 (Fig. 1b). Western blots of lysates from human THP1 macrophages, treated with the 5-arylnicotinamides and T1317, showed that all agents increased cellular ABCA1 protein (Fig. 1c). Real-time quantitative PCR (RT-qPCR) revealed that T1317 upregulated ABCA1 mRNA by ~5-fold (Fig. 1d) while, in contrast, Cpd H and Cpd J up to 25 μM showed no effect. Collectively, these data demonstrate that the 5-arylnicotinamides upregulate ABCA1 protein and cholesterol efflux by a non-transcriptional mechanism.

### Identification of OSBPL7 as a target of the 5-arylnicotinamides

**Fig. 2:**
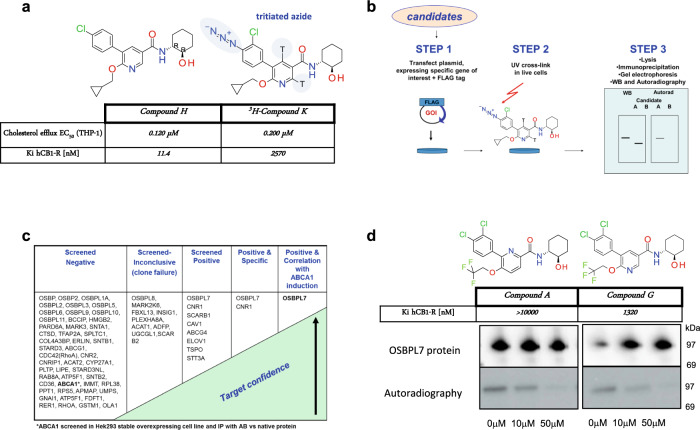
Chemical biology identifies OSBPL7 as the pharmacological target of the 5-arylnicotinamides. **a** Comparison of the potency of Cpd K (tritiated azide derivative) vs. Cpd H in apoAI-dependent cholesterol efflux from human THP1 cells. Cpd K retains similar potency as Cpd H validating Cpd K as an active chemical probe for target identification efforts. **b** Target identification screen flowchart: in step 1, a mammalian expression plasmid expressing a full-length protein for the candidate of interest as a fusion protein with a C-terminal FLAG tag was transfected into HEK293 cells; in step 2 ^3^H-Cpd K was added (1 µM), cells were incubated for 3 h, then exposed to UV light for 15 min; in step 3, cells were lysed, the expressed protein was purified by 1-step immunoaffinity using anti-FLAG antibody, proteins were separated on two identical SDS-PAGE gels, one was then subjected to WB to detect the expressed protein, and the second was dried and exposed to film (4 wks) to detect ^3^H-Cpd K binding to the expressed target. **c** Table showing the progression of the candidates screened according to the flowchart outlined in (**b**). Most candidate targets were properly expressed and demonstrated no binding of ^3^H-Cpd K. Several were not properly expressed (incorrect protein size, no protein detected) and were marked inconclusive. Seven targets showed binding of ^3^H-Cpd K but only OSBPL7 and CB1R emerged following competition binding experiments with unlabeled Cpd K. CB1R was excluded as a candidate since the high affinity ligand rimonabant (Cpd O) was inactive to induce cellular cholesterol efflux to apoAI (Fig. 3a). **d** Competition binding activities of Cpd G and Cpd A that were selected for studies in podocytes and kidney disease models. Data representative from three independent experiments. Both compounds bind to OSBPL7 as reflected by their ability to compete ^3^H-Cpd K interaction with OSBPL7 (full western blots shown in Supplementary Fig. 2).

We hypothesized that the activity of Cpd H to induce ABCA1 may be an off-target effect due to the lack of ABCA1 activity of unrelated CB1R ligands. For target deconvolution, a derivative of Cpd H with a photoactivatable azide functionality was synthesized and evaluated for cholesterol efflux activity. This derivative, Cpd K (5-(4-azido-3-chlorophenyl)-6-(cyclopropylmethoxy)-*N*-[(1*R*,2*R*)-2-hydroxycyclohexyl]-3-pyridine-carboxamide), was equally active as Cpd H to upregulate ABCA1 efflux in vitro (Fig. 2a), but had more than 200-fold reduced affinity for hCB1R. The azide group of Cpd K allows it to covalently crosslink to lysine residues when exposed to UV light. Cpd K was employed as a probe to identify protein target(s).

**Fig. 3:**
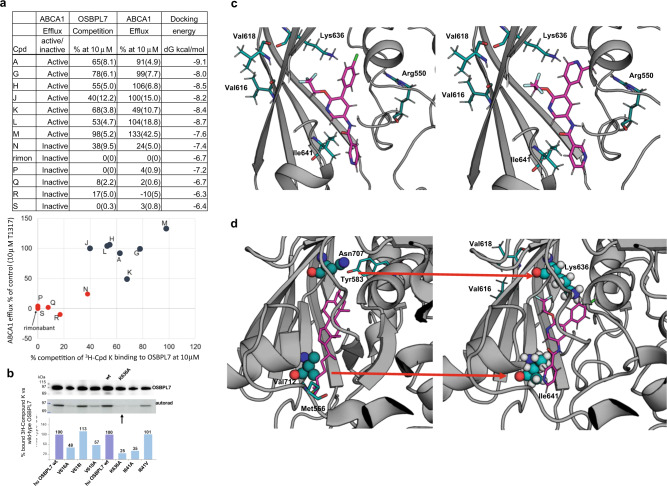
The 5-arylnicotinamides interact with the predicted OSBPL7 sterol binding domain. **a** Twelve 5-arylnicotinamides (7 active, 5 inactive) and rimonabant were used to explore the correlation between their ability to induce ABCA1-mediated cholesterol efflux in THP1 cells and their ability to compete with ^3^H Cpd K binding to OSBPL7. The table summarizes the ABCA1 efflux data for the selected set, the binding data results generated for OSBPL7, as well as the docking energies calculated from the homology model. This is the complete dataset including all Cpds evaluated for OSBLP7 binding. The graph depicts the relationship between ABCA1 efflux activity and ability to compete with ^3^H Cpd K binding to OSBPL7. Cpds A, G, H, J, K, L, M were scored as active (>50% increase in cholesterol efflux in THP1 cells vs. the effect of T1317) while Cpds N, P, Q, R, S and rimonabant were scored as inactive (<50% increase in cholesterol efflux). The efflux data strongly correlated with the ability of these Cpds to interact with OSBPL7. **b** Evaluation of ^3^H Cpd K binding to wildtype OSBPL7 (wt) and various single mutations introduced into the putative ORD. Mutation of K636A reduced ^3^H Cpd K binding by ~75% suggesting that Lys 636 is the site of covalent interaction via its reactive azide group. Representative data from three independent experiments, full western blot shown in Supplementary Fig. 3). **c** The predicted best poses and energies for binding of active Cpd M (left panel) to OSBPL7 in comparison to inactive Cpd R (right panel). Both compounds make a clear π-cation interaction with the catalytic Lys636. However, R, which has a higher GlideScore, fits the binding site less well due to the methyl group of the 4-pyridine ring which appears too close to the positively charged Arg550. **d** Left image: Focus on the binding site of the cholesterol/ORP1-ORD complex (pdb code 5zm5). Right image: Focus on the binding site of the Cpd M/OSBPL7 complex. The ligands are displayed as ball- and-stick models with the carbon atoms colored in magenta. The residues that occupy equivalent positions are indicated by the filled circles.

To identify candidate targets, we first undertook a global screen by compound capture mass spectrometry (CC-MS). Proteins identified by CC-MS were elaborated by addition of other candidates based on plausibility for their involvement in the ABCA1 pathway, leading to sixty-nine candidate targets of interest. Plasmids expressing each as a full-length protein with a C-terminal FLAG tag were obtained. Plasmids were individually transfected into HEK293 cells and then incubated in the presence of ^3^H-Cpd K, followed by UV cross-linking in situ, preparation of cell lysates, immunoprecipitation with anti-FLAG antibody, and then parallel western blotting to confirm protein expression, and autoradiography to evaluate ^3^H-Cpd K binding (Fig. 2b). Forty-nine candidates, including ABCA1 itself, were eliminated in a first screen due to no crosslinking to ^3^H-Cpd K (Fig. 2c). Eight candidates scored positive for interaction with Cpd K. These included OSBPL7, CB1R, SCARB1, CAV1, ABCG4, ELOV1, TSPO, and STT3A (Fig. 2c). To evaluate if ^3^H-Cpd K binding was specific, we conducted competition experiments by performing crosslinking in the presence of a 50-fold excess of unlabeled Cpd K. Unlabeled Cpd K effectively competed for ^3^H-Cpd K binding to OSBPL7 and CB1R, but not to the other six candidates, eliminating them as non-specific binders. We repeated the experiment to determine if Cpd A and Cpd G, both effective to induce ABCA1-dependent cholesterol efflux, compete for ^3^H Cpd K binding to OSBPL7. Both Cpd A and Cpd G were effective competitors, indicating that they also interact with OSBPL7 (Fig. 2d, Supplementary Fig. 2). To build further confidence in OSBPL7, we added a third filter through evaluation of a set of 12 structurally similar molecules plus rimonabant (Cpds A, G, H, J, K, L, M, N, P, Q, R, S). Seven of the compounds had been shown to upregulate ABCA1-mediated cholesterol efflux, while the other five and rimonabant were inactive (Fig. 3a). We found that the activity of these molecules to stimulate ABCA1 efflux correlated with their ability to compete for binding of ^3^H-Cpd K to OSBPL7. This positive “activity-activity” relationship between OBPL7 binding in the competition assay with upregulation of ABCA1 activity, strongly suggests that OSBPL7 is the pharmacologically relevant target of these ABCA1-inducing small molecules.

**Fig. 4:**
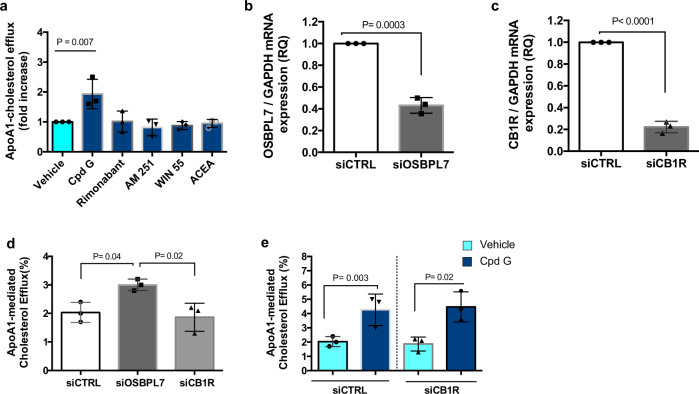
Effects of OSBPL7 and CB1R siRNA and CB1R agonists on ABCA1-mediated cholesterol efflux. **a** Effects Cpd G and several CB1R-targeted compounds on apoAI-mediated cholesterol efflux in THP1 cells. All compounds were used at a concentration of 5 µM. Data expressed as mean ± SD (*n* = 3). All treatments were compared to vehicle using one-way ANOVA, F(5, 12) = 6.5), followed by Dunnett’s test. *P* = 0.01 for Cpd G; *P* > 0.05 for the other Cpds. **b**, **c** Repression of OSBPL7 or CB1R mRNA following transfection of specific siRNAs in differentiated THP1 macrophages. Data expressed as mean ± SD (*n* = 3). Groups were compared using a double-tailed *t*-test. RNAi reduced OSBPL7 expression by ~60% (t(4) = 13.7, P = 0.0002) and CB1R expression by ~80% (t(4) = 26, *P* < 0.0001). **d** Effects of siRNAs targeting OSBPL7 and CB1R on apoAI-mediated cholesterol efflux from THP1 macrophages. Data expressed as mean ± SD (*n* = 3). Treatments were compared one to another using unpaired one-way ANOVA, F(2, 6) = 8.3, *P* = 0.019, followed by Tukey’s test: siRNA knockdown of OSBPL7 expression significantly increased cholesterol efflux when compared to non-targeting control (*P* = 0.04) and to siCB1R (*P* = 0.02), while knockdown of CB1R had no effect (*P* = 0.85). **e** Effect of Cpd G (5 µM) on apoAI-mediated cholesterol efflux from macrophages in which CB1R expression was repressed by siRNA. Data expressed as mean ± SD (*n* = 3). Groups were compared to each other using unpaired one-way ANOVA, F(3, 8) = 8.7), followed by Tukey’s test. When comparing Vehicle vs Cpd G: *P* = 0.04 in siCTRL, and *P* = 0.02 in siCB1R cells. Knockdown of CB1R expression had no effect on cholesterol efflux induced by Cpd G (si CTRL + Cpd G vs siCR1B + Cpd G, *P* = 0.99). For **a**–**e** only independent experiments were compared.

A potential contribution of CB1R remained, however, rimonabant, a structurally unrelated CB1R antagonist, did not induce ABCA1 cholesterol efflux (Fig. 3a; Fig. 4a). Several other CB1R agonists/antagonists including AM251, WIN55-212-2, and arachidonyl choloroethylamine (ACEA), were also inactive (Fig. 4a). Cpd A and Cpd G, carried forward into animal studies, were active to induce ABCA1 and to compete for Cpd K binding to OSBPL7, but had either no (Cpd A; Ki hCB1R = > 10,000 nM) or low (Cpd G; Ki hCB1R = 13,20 nM) binding affinity to the CB1R (Figs. 2d and 3a).

We performed siRNA knockdown experiments. Transfection of OSBPL7 siRNA decreased mRNA by about ~60%, stimulating a significant 50% increase in cholesterol efflux (Fig. 4b, d). In contrast, silencing of CB1R reduced mRNA by ~80%, but had no effect on cholesterol efflux, and did not influence the activity of Cpd G (Fig. 4c–e). These data confirm the role of OSBPL7 and exclude the CB1R in mediating the cholesterol efflux activity of the 5-arylnicotinamides.

### Homology modeling show the compounds occupy the OSBPL7 binding pocket

Oxysterol binding protein-related proteins, including OSBPL7, contain a highly conserved oxysterol regulatory domain (ORD) at their carboxy-terminus^25,26^. In order to predict the ligand-binding pocket, we created a homology model of OSBPL7 (UNIPROT: Q9BZF2) using the SWISSMODEL webserver^27^ (Fig. 3c). The crystal structure of human ORP1-ORD, in complex with cholesterol, has a homology of 42.5%, and was used as a template^28^. The preliminary model was first minimized using the molecular modeling package ALMOST to remove possible atom clashes, and then optimized to perform docking calculations using the “protein preparation wizard” utility within the Schrödinger suite for molecular modeling^29,30^. This approach ensured that the correct number of hydrogen atoms are present, all atoms are identified, that missing parts of the protein, that could not be modeled could be detected, and lastly, that the correct protonation state of all amino acids could be assigned.

Based on the model, we identified key amino acid residues predicted to line the OSBPL7 ligand-binding pocket, and introduced a series of single mutations. Each mutant was transfected into cells and tested for ^3^H-Cpd K binding. Mutation of lysine 636 to alanine reduced the ability of Cpd K to be crosslinked to OSBPL7 by ~75% (Fig. 3b). This indicated that Cpd K binds within the predicted sterol binding pocket, and implicates Lys636 as essential for its covalent interaction with OSBPL7. The homology model also predicts that a hydrophobic beta sheet lines the pocket, defined by amino acids that are conserved in the ORDs of other OSBPs (Ile641, Val616, Val618, and Lys636). Mutation of Ile641 to Ala decreased Cpd K binding, suggesting that Cpd K occupies the entirety of the pocket (Fig. 3b, Supplementary Fig. 3 and Fig. 3c). Substitution of Val616 or Val618 by Ala also reduced Cpd K binding, while conservative substitutions such as Val619Ile and Ile641Val were tolerated (Fig. 3b, c). These data reveal that compound K binds intimately within the predicted sterol binding pocket of OSBPL7.

We then performed dynamic docking simulations of the ligands to the OSBPL7 model using the program Glide (Glide, Schrödinger, LLC, New York, NY, 2019)^30^. A grid was defined with an outer box of 30 Å and an inner box of 10 Å, centered around each ligand, virtually co-crystallized with the template. Since Lys636 plays a key role in binding of Cpd K to OSBPL7, the orientation of this residue was carefully analyzed using the rotamers selection tool in Maestro. Two rotamers were found as most probable; one oriented towards the binding site, and one towards the interior of the protein. Since the second orientation did not allow interactions with the compounds, the first rotameric state of Lys636 was selected for docking studies.

Compound docking was performed by the extra precision procedure and scoring function (GlideScoreXP). For each molecule, the best docking solution was identified using the GlideScore. The calculated binding energies for the individual molecules are shown in Fig. 3a. The average score of the molecules that bind OSBPL7 is −8.2 kcal/mol, while the average for molecules not or poorly binding to the protein is higher; −6.8 kcal/mol. This indicates that the energies of the different poses recapitulate qualitatively the degree of binding of the various molecules. Binding of the active Cpd M, is shown in Fig. 3c (left panel), while the best pose of compound R, which had no activity, is shown in Fig. 3c (right panel). Both compounds make a clear π-cation interaction with Lys636. However, R, which has a higher GlideScore, fits less well due to the methyl group of the 4-pyridine ring which appears too close to the positively charged Arg550. We also compared the OSBPL7 model with the structure of the ORP1-ORD (oxysterol recognition domain) in complex with cholesterol (pdb code 5zm5). We observed that I641 corresponds to V712 and that K636 corresponds to N707 (Fig. 3d). Visual inspection of the ORP1-ORD/cholesterol complex indicates that these two residues contact the substrate and, particularly in the case of N707, form an H-bond with cholesterol, significantly contributing to binding. Considering the similar physicochemical properties of valine and isoleucine, and the conserved ability to form H-bonds of lysine with respect to asparagine, it is reasonable to conclude that I641 and K636 make a similar contribution to the binding of cholesterol to the OSBPL7 ORD.

### The 5-arylnicotinamides increase ABCA1 in cultured human podocytes

**Fig. 5:**
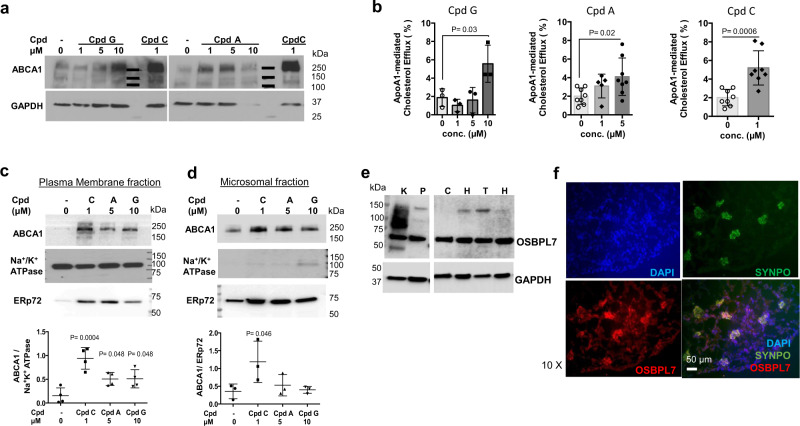
Compound treatment increases ABCA1-mediated cholesterol efflux in human podocytes. **a** Representative ABCA1 and GAPDH Western blots from podocytes treated with Cpd G (left), Cpd A (right), and the LXR agonist Cpd C, at the concentrations indicated (full western blots shown in Supplementary Fig. 4). **b** apoAI-mediated cholesterol efflux in podocytes treated with Cpd G (left), Cpd A (middle) and Cpd C (right) at the concentrations indicated. Data expressed as the mean ± SD. Treatments with Cpd G or Cpd A were compared to vehicle using one-way ANOVA, followed by Dunnet’s test. *P* = 0.03 for Cpd G at 10 µM, *n* = 3, F(3, 8) = 6.9; *P* = 0.02. for Cpd A, *n* = 8, F(2, 18) = 3.9; *P* > 0.05 for other concentrations. Cpd C and vehicle were compared using a two tailed *t* test: *n* = 8, t(14) = 4.4, *P* = 0.0006. **c**, **d** Representative western blots for ABCA1, Na^+^/K^+^ ATPase, and ERp72 in plasma membrane (**c**) and microsomal (**d**) enriched fractions from podocytes treated with Cpd C (1 µM), Cpd A (5 µM) or Cpd G (10 µM). Full western blots shown in Supplementary Fig. 5. Below, relative ABCA1 quantification normalized to the cellular marker. Samples derived from the same experiment and blots were processed in parallel. Data expressed as the mean ± SD. Treatments compared to vehicle using one-way ANOVA, followed by Dunnett’s test. *P* = 0.0004, 0.048 and 0.048 for Cpd C, Cpd A, and Cpd G, respectively, in plasma membrane fractions, *n* = 4, F(3, 12) = 12.3. For microsomal fractions, *P* < 0.05 only for Cpd C (*P* = 0.046), *n* = 3, F(3, 8) = 3.7. **e** Representative western blot showing OSBPL7 and GAPDH expression from human kidney (K) and podocytes (P). Total proteins from Caco2 (C), HepG2 (H), T (THP1), and HEK293 (H) were also analyzed. Uncropped western blots are shown in Supplementary Fig. 7. **f** Immunofluorescence staining of mouse kidney cortex showing OSPBL7 (red) expression in glomeruli and tubules. Nuclei stained with DAPI (blue). Synaptopodin (green) was used as a podocyte marker.

From the 5-arylnicotinamide series, Cpds A and G were explored for effects on ABCA1 expression and cholesterol efflux in renal podocytes, in comparison to the LXR agonist Cpd C. Cpd C is a more potent LXR agonist of the same hexafluoroisopropanol class of molecules as T1317^31^. ABCA1 expression was analyzed by western blot in whole-cell lysates from podocytes after drug treatment. The ABCA1 inducers Cpd G (Fig. 5a left panel, Supplementary Fig. 4a, c) and Cpd A (Fig. 5a right panel, Supplementary Fig. 4b, d) significantly increased ABCA1 protein levels at 10 µM and 5 µM concentrations, respectively, but to a lesser extent than the LXR agonist, Cpd C.

We then measured apoAI-mediated cholesterol efflux from podocytes (Fig. 5b). Cpd G, Cpd A, and Cpd C all significantly increased apoAI-mediated cholesterol efflux at 10 µM, 5 µM, and 1 µM, respectively, to approximately the same extent; 2-fold vs. untreated cells. Interestingly, the increase in efflux induced by Cpd G and Cpd A was similar to the increase induced by the LXR agonist Cpd C, despite the latter having induced higher levels of whole-cell ABCA1 protein in the WB experiment (Fig. 5a). We performed cell fractionation experiments to assess the subcellular distribution of ABCA1 after drug treatment (Fig. 5c, d, Supplementary Fig. 5). These experiments revealed that the 5-arylnicotinamides (Cpds G and A) increased ABCA1 at the plasma membrane (Fig. 5c), but unlike the LXR agonist, they did not increase ABCA1 in microsomal fractions (Fig. 5d). No ABCA1 was detected in the organelle-free cytosolic fraction (Supplementary Fig 5, right panel). These results suggest that Cpd A and Cpd G may stabilize ABCA1 at the plasma membrane.

To address the question of whether ABCA1 and OSBPL7 physically interact, we performed co-immunoprecipitation (coIP) by transfecting HEK293 cells with plasmids that constitutively overexpress OSBPL7-V5 and ABCA1-FLAG tagged proteins. We used anti-FLAG beads to immunoprecipitate ABCA1 and bound proteins, separated by SDS-PAGE and western blotted with antibodies to detect ABCA1 and OSBPL7-V5. While both proteins were overexpressed, and we were able to immunoprecipitate ABCA1-FLAG, we did not detect OSBPL7-V5 under these experimental conditions (Supplementary Fig. 6).

### OSBPL7 is expressed in renal podocytes

Western blot analysis was performed in lysates from human podocytes and kidney tissue. We found that OSBPL7 is present in human kidney and human podocytes, as well as in other human cell lines (Caco2, HepG2, THP1 and HEK293) (Fig. 5e, Supplementary Fig. 7). Immunostaining of renal cross sections revealed that OSBPL7 is expressed in the renal cortex including in glomeruli, where it colocalizes with synaptopodin, a podocyte-specific marker (Fig. 5f).

### Cpds G and A reduce kidney injury in adriamycin-induced nephropathy

In total, about 180 5-arylnicotinamides were synthesized and evaluated in THP1 cellular efflux assays. Of these molecules, Cpds G and A were chosen for in vivo studies. This was based upon ABCA1 cholesterol efflux activity, metabolic stability in rat and human liver microsomes, low cytochrome P450 interaction potential, and low potential for covalent protein binding. These data, and rat single-dose pharmacokinetics for Cpd G, are summarized in Supplementary Information Table S2. Cpd G and Cpd A were tested in the Adriamycin (ADR)-induced renal disease model. ADR nephropathy reflects proteinuric kidney disease, and is the most common experimental model of focal segmental glomerulosclerosis (FSGS). Key hallmarks of the ADR model include profound proteinuria, weight loss, and glomerular histological changes resembling FSGS^32,33^.

**Fig. 6:**
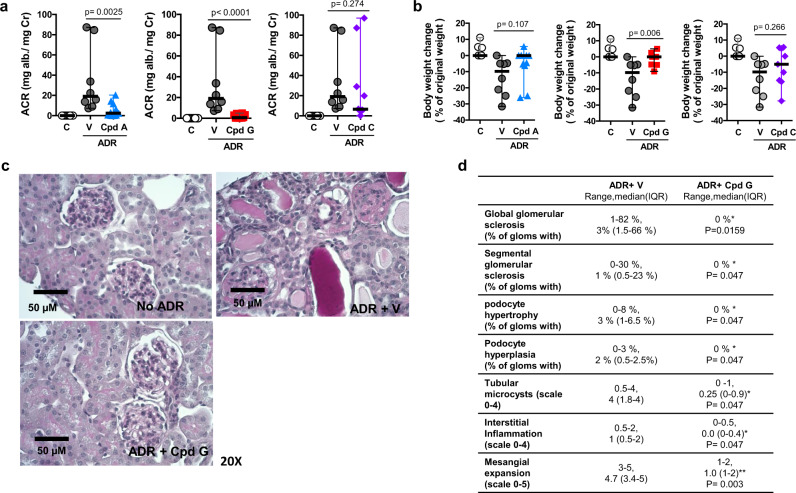
Cpd G protects from kidney injury in a mouse model of adriamycin-induced nephropathy. **a**–**d** Renal phenotype of mice that received vehicle (*n* = 8), Cpd A (30 mg/Kg, *n* = 11), Cpd G (100 mg/Kg, *n* = 10) or Cpd C (10 mg/Kg, *n* = 8) after adriamycin (ADR) injection. A group that did not receive ADR injection (C), is included to show the healthy phenotype. **a** Albumin to creatinine ratio in spot urines collected 4 weeks after treatment with vehicle (V) or with Cpd A (left), Cpd G (middle) or Cpd C (right). Data expressed as median and range. Treatments with vehicle or compound were compared using a two-tailed Mann–Whitney test: Cpd A (*U* = 9, *P* = 0.0025); Cpd G (*U* = 0, *P* < 0.0001); Cpd C (*U* = 24, *P* = 0.286). **b** Weight change, as % of weight at baseline, after 4 weeks of treatment with vehicle or with Cpd A (left), Cpd G (middle) or Cpd C (right). Data expressed as median and range. Groups treated with vehicle or compound were compared using a two-tailed Mann–Whitney test: Cpd A (*U* = 25, *P* = 0.107); Cpd G (*U* = 11, *P* = 0.006); Cpd C (*U* = 24, *P* = 0.274). **c** Representative images of PAS-stained kidney sections (magnification ×20) from mice injected with saline solution (no ADR) and mice injected with ADR that received vehicle (ADR + vehicle) or Cpd G (ADR + Cpd G). **d** Table summarizing the quantification of histological indicators of kidney damage obtained by a blinded pathology analysis of the PAS-stained kidney sections from mice that received vehicle or Cpd G after ADR injection. Glomerular damage was quantified and is expressed as the percentage of glomeruli with the indicated phenotype. The scale used to quantify tubular microcysts and interstitial inflammation represent the following extent of damage: 0: 0%; 0.5: 1–10%; 1: 11–25%; 2: 26–50%; 3: 51–75%; 4: >75%. Data expressed as the range and median (interquartile range). Both groups were compared using a double-tailed Mann–Whitney test (*n* = 5), *P < 0.05, **P < 0.01, the exact *P* values are indicated.

We performed dose-finding studies for preliminary assessment of efficacy and dose selection for subsequent studies. ADR-challenged Balb/c mice were treated with compound A or G via oral gavage at 30 mg/kg or 100 mg/kg once per day starting one-day post-ADR injection. The LXR agonist, Cpd C, at a dose of 10 mg/kg, was used as a comparator (Supplementary Fig. 8). To induce kidney injury, adriamycin was administered at a dose of 12 mg/kg of body weight. Urine albumin to creatinine ratios (ACR) and body weight were determined at days 7, 14, 21, and 28 post injection. ADR-challenged mice demonstrated severe transient proteinuria and body weight loss at all time points. ACR values were significantly improved in animals treated with 30 mg/kg/day or 100 mg/kg/day of Cpd A or 100 mg/kg of Cpd G and, to a lesser extent, by 30 mg/kg of Cpd G. (Supplementary Fig. 8). Immunofluorescence microscopy of renal cortex sections revealed ADR-induced glomerular degeneration with podocyte loss, and loss of ABCA1 expression. All of these effects were prevented by treatment with Cpd G (Supplementary Fig. 9). Based on the pilot study, optimal treatment doses for Cpd A and Cpd G were determined to be 30 mg/kg/day and 100 mg/kg/day, respectively. The second study used a larger number of animals and albumin to creatinine ratios were determined 28 days post treatment (Fig. 6a). Consistent with preliminary results, ADR-challenged mice (ADR+V) developed severe proteinuria. Treatment with Cpd A (ADR+A) (Fig. 6a, left) and, more strikingly, Cpd G (ADR+G) (Fig. 6a, middle), showed 8 and 30 times lower ACR, respectively, with a median IQR of 2.3 (0.3–12 mg albumin/mg creatinine) for Cpd A and 0.6 (0.1–3.6 mg albumin/mg creatinine) for Cpd G. Control ADR-challenged mice had a median IQR of 19 (10–72 mg albumin/mg creatinine). Mice treated with the LXR agonist Cpd C demonstrated more variable reduction in ACR (Fig. 6a, right).

Cpd A (Fig. 6b, left) and Cpd G (Fig. 6b, middle) also substantially reduced weight loss. Over the 28-days after ADR challenge, mice in the vehicle group lost up to 30% of body weight. In contrast, 7 out of 10 mice that received Cpd G showed no weight loss, with only minor loss less than 5% for the remaining 3 mice (Fig. 6b, left). LXR agonist Cpd C had no significant effect to prevent weight loss (Fig. 6b, right).

Since Cpd G had the greatest efficacy to reduce proteinuria and weight loss, we performed histological analysis on kidney sections (Fig. 6c). ADR challenge induced extensive histological changes, including a high degree of focal and segmental glomerulosclerosis, accompanied by tubular microcysts, interstitial inflammation, and mesangial expansion (Fig. 6d). Cpd G significantly reduced global and segmental glomerulosclerosis, reduced podocyte hypertrophy and hyperplasia, and reduced tubular microcysts and interstitial inflammation (Fig. 6d). Cpd G also blunted ADR-induced inflammation, reflected by reduced IL1β and MCP-1 in kidney cortex (Supplementary Fig. 10). These data demonstrate that Cpd G is highly efficacious to reduce hallmarks of FSGS, most notably, by improvement in ACR and prevention of ADR-induced body weight loss (Fig. 6).

### Effects of Cpd G on plasma parameters and renal and liver lipids

Plasma lipids, hematological parameters, and liver transaminase levels were evaluated in all groups after 28 days of treatment. No significant differences in plasma total cholesterol or triglycerides were found. However, sporadically high cholesterol levels (>122 mg/dL, 99.5th percentile of the healthy control group) were detected in 50% of ADR-challenged animals that received vehicle, 10% that received Cpd A, 33% that received Cpd C, and none that received Cpd G (Supplementary Fig. 11a, Supplementary Table 3). Hypercholesterolemia was observed only in mice with particularly severe proteinuria (ACR > 15 mg albumin/mg creatinine). No changes in triglyceride levels were observed (Supplementary Fig. 11b). Hematocrit, hemoglobin, white blood cell count, and ALT and AST values are shown in Extended Data Table 2. No differences were detected in blood cell counts. Adriamycin treatment increased ALT and AST, which was partially attenuated by Cpd G (Supplementary Table 4). Total cholesterol and triglyceride content in kidney cortexes remained unchanged, although higher triglyceride content was observed in about 30% of animals that received the LXR agonist Cpd C (Supplementary Fig. 11c, d). Liver total and esterified cholesterol (CE) levels also remained unchanged in ADR-challenged animals. (Supplementary Fig. 11e,f). The reduction in hepatic triglyceride content due to ADR challenge, was countered by drug treatment. Cpd A and Cpd C, but not Cpd G, increased hepatic triglyceride content vs. vehicle-treated controls (Supplementary Fig. 11g).

### Cpd G reduces renal failure in a mouse model of Alport syndrome

Many forms of familial steroid-resistant FSGS and other forms of CKD are caused by mutations in Col IV genes^34–36^. We recently reported decreased ABCA1 expression in glomeruli from Col4a3 knockout mice (Col4a3 KO), a mouse model of AS^13^. Col4a3 knockout mice do not express Collagen type IV. These mice develop severe albuminuria, high blood urea nitrogen (BUN), and increased serum creatinine levels starting at 4 weeks of age, progressing rapidly toward end stage renal disease (ESRD). Animals die of kidney failure by 8 weeks of age^37^.

**Fig. 7:**
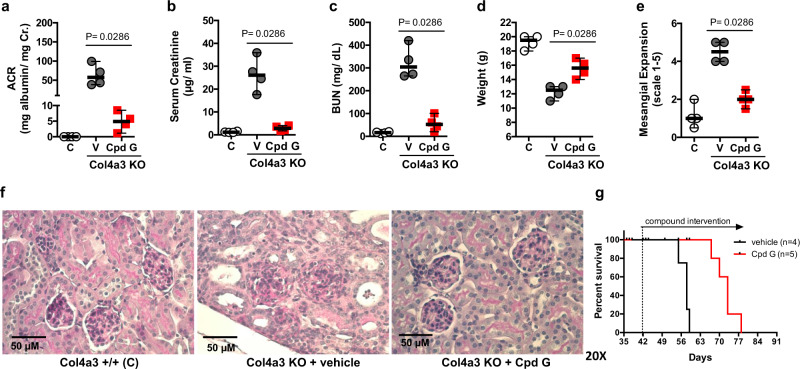
Cpd G protects from renal injury in the mouse model of Alport syndrome. **a**–**f** Treatment outcomes of 8-week-old 129-Col4a3 KO after receiving vehicle (V) or Cpd G (100 mg/Kg/day) for 28 days. A group of Col4a3+/+ littermates (C) is included to show the healthy phenotype. **a** Albumin to creatinine ratio in spot urine samples; **b** serum creatinine levels; **c** blood urea nitrogen (BUN) levels; **d** body weights; **e** mesangial expansion scores determined in a blinded manner by a pathologist in PAS-stained kidney sections; **f** representative pictures of PAS-stained kidney sections from each treatment group. **a**–**e** Data expressed as the median and range. Groups treated with vehicle or Cpd G were compared using a double-tailed Mann–Whitney test (*n* = 4, *U* = 0, *P* = 0.0286); **g** survival curve of Col4a3 KO mice that received vehicle or Cpd G (100 mg/Kg/day) for 4 weeks starting at the age of 6 weeks.

Cpd G (100 mg/kg), or vehicle, were administered to Col4a3 KO mice daily, by oral gavage, starting at 4 weeks of age when mice first present with signs of renal insufficiency. Treatment was continued for 4 weeks, and surviving mice were sacrificed at 8 weeks of age. Albuminuria (Fig. 7a), serum creatinine (Fig. 7b), blood urea nitrogen (Fig. 7c) and the extent of weight loss (Fig. 7d) were significantly reduced in Col4a3 KO mice treated with Cpd G. In comparison with animals that received ramipril from a prior study with equal design^38^, albuminuria was significantly reduced in animals that received Cpd G and the effect of Cpd G on BUN and serum creatinine was not inferior to ramipril (Supplementary Fig. 12). Histological analysis of kidney sections revealed significantly less mesangial expansion in Cpd G treated mice (Fig. 7e, f). Cpd G had no effect on serum cholesterol (Supplementary Fig. 13a), but tended to reduce serum triglycerides (Supplementary Fig. 13b). Cpd G did not affect total cholesterol or triglyceride content in kidney cortexes (Supplementary Fig. 13c, d).

To explore if Cpd G may be of benefit in advanced kidney failure, we performed a mortality study in which treatment with Cpd G or placebo was initiated at 6 weeks of age, a time when significant renal failure has occurred. We found that Cpd G reduced mortality in Col4a3 KO mice, resulting in an increased life span of approximately 15% compared to controls (Fig. 7g).

### Cpd G reduced renal cholesterol ester content in both disease models

**Fig. 8:**
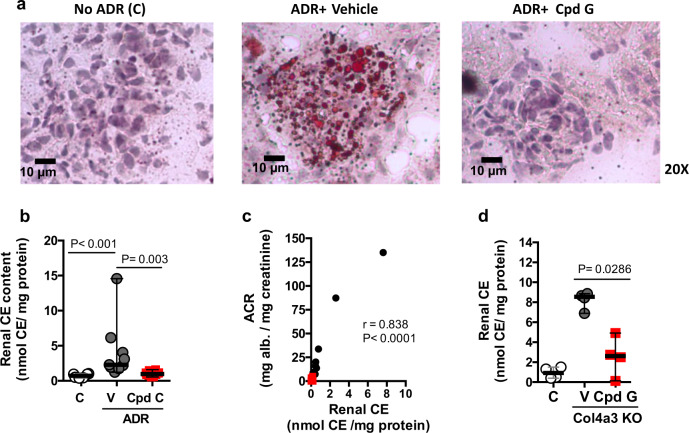
Cpd G reduced accumulation of cholesterol esters in renal cortex in both disease models. **a**–**c** Lipid accumulation in kidneys from mice that received vehicle (V, *n* = 8) or Cpd G (*n* = 10) for 28 days after ADR challenge. A group that did not receive ADR (C) (*n* = 10), is included to show the healthy phenotype. **a** Representative image of Oil-red O (ORO) stained kidney sections; **b** cholesterol ester (CE) content, normalized to total proteins in kidneys. Data expressed as the median and range. Differences with regard to animals injected with ADR that received vehicle were calculated using Kruskal–Wallis, followed by Dunn’s test: no ADR, *P* < 0.001; ADR+ Cpd G, *P* = 0.003; **c** cholesterol ester content in kidneys from individual animals vs the corresponding albuminuria (ACR) values at day 28 of treatment. The data points in red correspond to the group that received Cpd G. A two-tailed Spearman correlation coefficient test was used to assess the relationship between the variables (*n* = 17 pairs, *r* = 0.838, *P* < 0.0001). **d** Cholesterol ester content (CE), normalized to total proteins, in the kidney cortexes from 8-week-old Col4a3 KO mice, that received vehicle or Cpd G for 28 days. A group of Col4a3+/+ littermates of the same age (C), is included to show the healthy phenotype. Data expressed as the median and range. Col4a3 KO mice treated with vehicle or with Cpd G were compared using a double-tailed Mann–Whitney test (*n* = 4, *U* = 0, *P* = 0.0286).

Glomerular cholesterol and lipid accumulation are well known to be associated with the progression of kidney disease^7–10,12–14^ Kidney cortexes from mice that received vehicle after ADR challenge, but not those that received Cpd G, showed deposition of lipid droplets particularly in glomeruli, and a significant increase in cholesterol ester content (Fig. 8a, b). The increased cholesterol ester content strongly correlated with albuminuria (Fig. 8c). Cpd G also significantly reduced cholesterol ester content in kidneys of Col4a3 KO mice (Fig. 8d). Despite the reduction in cholesterol ester in kidney, there was no change in plasma cholesterol in Col4a3 mice and only in about 50% of the ADR mice (Supplementary Fig. 11a and Supplementary Fig. 13a). These data suggest that Cpd G may be protective by reducing cholesterol and lipid content in kidney.

## Discussion

The present study describes the identification of a class of compounds by phenotypic drug discovery (PDD) that upregulate ABCA1-dependent cholesterol efflux. Target deconvolution revealed OSBPL7 to be the molecular target. In animal models of kidney disease, these compounds reduced renal lipid accumulation, prevented podocyte loss, normalized proteinuria, and reduced renal function decline.

Current therapies for chronic kidney disease are limited to ACEi, ARBs and, more recently, sodium-glucose cotransporter-type 2 inhibitors (SLGT2i). Despite the effectiveness of these agents in delaying progression of chronic kidney disease, primarily in patients with proteinuric kidney disease, the unmet medical need in CKD remains immense^3,39,40^. In a randomized trial of 4300 patients, SGLT2i were unexpectedly found to be efficacious regardless of the presence of diabetes^41^, suggesting that mechanisms of renoprotection other than glucose lowering are involved. In fact, a recent study demonstrated that SGLT2i can increase plasma HDL and may reduce cardiac fat accumulation^42^. This recent emergence of SGLT2i underscores the benefit of targeting multiple CKD driving mechanisms to develop more effective therapies.

We, and others, have described glomerular cholesterol accumulation in renal diseases arising from both metabolic and nonmetabolic origins. Glomerular lipid accumulation, in association with decreased ABCA1-mediated cholesterol efflux, occurs in DKD^7–11^, FSGS^12–14^, and AS^13^. These data suggest that restoring ABCA1 function may be effective in treating these types of renal disease. We recently demonstrated that renal cholesterol depletion with cyclodextrin, or genetic ABCA1 overexpression, protect from renal dysfunction in models of DKD^9^, FSGS^13,14^, and AS^13^. We also demonstrated that podocyte-specific ABCA1 deficiency worsens the renal phenotype in DKD^7^. Studies with LXR agonists, such as T1317, have also provided strong support for a key role of ABCA1, which has remained an attractive therapeutic target despite the limitation of these agents^22,43^.

We searched for agents by phenotypic drug discovery and optimization of candidate small molecule drugs^44^. We identified a series of 5-arylnicotinamides, several representatives of which increased ABCA1 protein levels and cholesterol efflux from cholesterol-loaded cells. The absence of efflux activity in fibroblasts from a patient with Tangier disease, and the absence of an increase in ABCA1 mRNA, revealed a new mechanism of action.

Target deconvolution efforts identified OSBPL7 as a high interest target. The molecular probe (^3^H-Cpd K) efficiently bound OSBPL7 in living cells, and could be effectively competed with unlabeled compound. More importantly, we demonstrated a correlative “activity-activity” relationship for a larger set of compounds between their ability to increase ABCA1 cholesterol efflux and their ability to compete for binding of the probe to OSBPL7. Mutational analysis, combined with molecular modeling and docking studies, indicate direct interaction of the compounds with the predicted OSBPL7 sterol binding pocket. Lastly, siRNA studies confirmed the involvement of OSBPL7 in ABCA1 function.

We also identified CB1R as a potential target. Several reports suggest a role for endocannabinoid signaling in cellular cholesterol regulation. Rimonabant was shown to modestly upregulate scavenger receptor B1 (SR-B1) and ABCG1, but not ABCA1, consistent with our data^45^. The synthetic cannabinoid WIN55,212-2 was shown to reduce expression of ABCA1, while increasing scavenger receptor CD36 in oxLDL-loaded RAW264.7 macrophages^46^. Together these data do not support a strong role for CB1R in regulation of ABCA1. In our studies, rimonabant and other CB1R agonists/antagonists including AM251, WIN55-212-2, and arachidonyl choloroethylamine (ACEA), were inactive to induce ABCA1 cholesterol efflux (Figs. 3a,  4a).

Mammalian oxysterol binding proteins (OSBPs) were originally implicated in the biosynthesis of cholesterol and sphingomyelin^47–49^. Several studies have linked specific OSBPs with regulation of ABCA1 protein stability and ABCA1-mediated cholesterol efflux^50,51^. No data prior to the present report, has implicated OSBPL7 as a regulator of ABCA1. Our data suggest that OSBPL7 may provide a chaperone or substrate transfer function affecting ABCA1. Preliminary co-immunoprecipitation experiments in transfected cells did not identify a direct interaction between the two proteins. However, a meta-analyses of genome-wide association studies (GWAS) identified OSBPL7 among 60 genetic loci influencing plasma cholesterol levels in humans^52^.

Our long-term interest in the role of ABCA1 in kidney disease led us to evaluate the 5-arylnicotinimides in in vitro and in vivo models of renal disease. We found that OSBPL7 is expressed in kidney, including in glomeruli and podocytes, and that Cpd A and Cpd G upregulate apoAI-mediated cholesterol efflux from cholesterol-loaded podocytes. Cpd A and Cpd G promote an increase of ABCA1 at the plasma membrane without affecting mRNA expression. This mechanism contrasts with LXR agonists, which upregulate ABCA1 mRNA leading to increase in ABCA1 in both plasma and intracellular membranes. We evaluated the therapeutic efficacy of Cpd A and Cpd G in vivo in a mouse model of FSGS (ADR-induced nephropathy). Both agents were found to reduce renal cholesterol content, correlating with reduced proteinuria, weight loss, and renal pathological changes. Cpd G was highly effective to prevent ADR-induced podocyte loss and glomerular structural changes. The significant reduction of ABCA1 in kidney, accompanied by an increase in inflammatory proteins, was prevented by Cpd G, supporting a key role for ABCA1. Cpd G effectively reduced albuminuria, BUN and serum creatinine levels in AS mice. More importantly, Cpd G prolonged lifespan in older Col4a3 animals with advanced renal failure.

These data support the therapeutic potential of compounds targeting ABCA1 and strongly suggest that this new mode of action will offer additional, or complementary efficacy to drugs targeting RAAS, SGLT2, NRF2, and cholesterol synthesis or absorption. Notably, in contrast to the effect of Cpd G, RAAS blockade does not prolong lifespan in older Col4a3 mice, despite being the most effective therapy for early intervention in this model^53,54^. The antiproteinuric activity of Cpd G was superior to the effects of ramipril we previously reported in a syngeneic model of AS^38^. Cpd G may therefore better target the population of patients with AS and proteinuria when compared to bardoxolone, an NRF-2 agonist that was shown to increase GFR but had no effect on proteinuria, the most powerful predictor of 10-year ESRD risk in the general population^55,56^. Statins were found to be protective in experimental AS^57^, and are recommended in all CKD patients to reduce cardiovascular risk, but have proven ineffective to slow progression of kidney disease^58^. Lastly, we recently reported a potential benefit of ezetimibe in AS by reduction of fatty acid and triglyceride absorption. However, ezetimibe did not affect kidney cholesterol content, and its efficacy to improve GFR and reduce proteinuria was inferior to the effects we report here for Cpd G^38^.

In summary, the present report describes the identification by phenotypic drug discovery of first in class drug-like small molecules that induce ABCA1-mediated cholesterol efflux. Chemical biology identified OSBPL7 as the molecular target and demonstrated that the molecules interact intimately within its predicted oxysterol binding domain. To our knowledge, this is one of only a few examples of drug-like compounds initially discovered by phenotypic approaches, whose target was subsequently successfully identified^44^. Compounds G and A were highly efficacious to improve renal function in mouse models reflecting two diverse etiologies of progressive renal disease. They appear to be safe and well-tolerated, as reflected by their ability to reduce AST levels in ADR-challenged mice and, more compellingly, to prolong the lifespan of Col4A3 KO mice. These compounds may offer a potential new therapy for renal disease by targeting cellular cholesterol metabolism through a new mode of action. These agents, along with current drugs targeting blood pressure or glucose control, may address the significant unmet need in CKD.

## Methods

### Compounds

The compounds used in this study are summarized in Supplementary Table 5 and were synthesized at F. Hoffmann-La Roche Ltd., Basel, Switzerland, unless otherwise indicated. For in vitro experiments, lyophilized compounds were reconstituted in DMSO (Sigma) and aliquots were stored at −20 °C. For in vivo experiments, lyophilized compounds were resuspended in vehicle containing 1.25% hydroxypropyl methyl cellulose, 0.10% docusate sodium salt, 0.18% propyl paraben sodium, and 0.02% citric acid monohydrate, at pH 6. A fine particle suspension was generated by three brief pulse sonications performed on ice.

### Synthetic chemistry procedures and analytical data

Please refer to Supplementary Data 1 file.

### Chemical biology/candidate target screening

Briefly, a plasmid expressing a candidate target of interest fused with a FLAG tag was transfected into HEK293 cells. After 24 h, tritiated compound K at a concentration capable of inducing ABCA1-mediated cholesterol efflux (1.0 μM) was added for 3 h, then the live cells were exposed to UV-light (ʎ = 336 nm) to query potential compound/target cross-linking in situ. Cell lysates were prepared, the expressed protein was then selectively immunoprecipitated using an anti-FLAG antibody coupled to sepharose beads, bound proteins were separated by SDS-PAGE, and two identical blots were prepared and probed by western blot and by autoradiography to detect protein expression and presence or absence of compound crosslinking, respectively. The flow chart of this screen is illustrated in Fig. 2. Figure 2d illustrates the ability of Cpd A and Cpd G to displace tritiated Cpd K from overexpressed and immunoprecipitated OSBPL7. Figure 3b illustrates the use of this assay to detect binding of tritiated Cpd K to overexpressed and immunoprecipitated variants of OSBPL7 containing point mutations in the predicted oxysterol binging pocket of OSBPL7. The advantages of this method are: i. the use of plasmid transfection leads to high expression of each candidate which reduces the likelihood to miss an authentic target for those which may be expressed at low endogenous levels, ii. the interaction of the chemical probe with its target occurs in a relevant biological context (live cells) at a pharmacologically active concentration, and iii. immunoprecipitation allows the expressed protein of interest to be purified from background proteins, substantially improving the signal/noise ratio.

### Cell culture

Cell lines were purchased from ATCC and subcultured for no more than passage 15, except for human podocytes, which were originally from Dr Jochen Reiser. Conditionally immortalized human podocytes were cultured under permissive conditions at 33 °C, in RPMI medium (Corning) containing 10% FBS (GIBCO), 1% penicillin/streptomycin, 0.01 mg/ml recombinant human insulin, 0.0055 mg/ml human transferrin (substantially iron-free), and 0.005 μg/ml sodium selenite (ITS, Corning). To induce differentiation, podocytes were cultured at 37 °C for 14 days in RPMI medium containing 10% FBS (GIBCO) and 1% penicillin/streptomycin. Differentiated podocytes were starved in RPMI-0.2% FBS for 18 h and then treated with 1 μM, 5 μM, and 10 μM of compounds freshly prepared in DMSO for 18 h at 37 °C as indicated.

### Cholesterol efflux in macrophages and fibroblasts

The ability of compounds to induced ABCA1-mediated cholesterol efflux was determined in replicate cultures of THP1 cells or fibroblasts in 96-well microplates. Cells were plated at a density of 150,000 cells/well. THP1 monocytes were differentiated to macrophages by the addition of PMA (100 nM) for 72 h in RPMI medium containing 10% fetal bovine serum and 3 µl of 2-mercaptoethanol. Cells were washed once with RPMI-1640 and then loaded with 50 µg/ml acetylated LDL, and 10 µCi/ml [^3^H]-cholesterol in RPMI-1640 medium containing 2% FCS, for 48 h at 37 °C. After loading, cells were washed once with RPMI-1640 and incubated with test compounds for an additional 24 h in RPMI-1640 medium containing 1 mg/ml fatty acid free-bovine serum albumin (BSA). Cells were then washed once, and cholesterol efflux induced by the addition of 10 µg/ml of human apoAI or 30 µg/ml of HDL2, HDL3, or LDL in RPMI-1640 containing 1 mg/ml BSA for an additional 6 h. Radioactivity in culture supernatants was determined by scintillation counting. Cholesterol efflux was expressed as a relative value vs. controls incubated in the absence of cholesterol acceptors. Dose-response curves were calculated using XLfit3 (ID Business Solutions Ltd. UK).

### Cholesterol efflux in podocytes

Human podocytes differentiated for 13 days were labeled for 24 h at 37 °C in RPMI medium containing 2% FBS and [^3^H]-cholesterol (1 μCi/ml, American Radiolabeled Chemicals). Cells were then washed 3 times with PBS and incubated with RPMI supplemented with 0.2% fat free-BSA (Sigma) with or without compounds for 18-h at 37 °C. The compounds were used at the following concentrations: Cpd C (1 μM), Cpd A (1 μM, 5 μM) and Cpd G (1 μM, 5 μM, 10 μM). Following the 18-h incubation, human apoAI (Calbiochem) was added to the media, (20 μg/mL final concentration) and the cells were incubated for another 18 h at 37 °C. Aliquots of medium collected before (*T*  = 0 h) and after (*T* = 18 h) the addition of apoAI were centrifuged at 12,000 × *g* for 5 min and the radioactivity in the supernatant was counted by liquid scintillation. Cells were then washed with PBS, lysed 0.1% SDS, 0.1M NaOH and the radioactivity in the lysates was quantified by liquid scintillation. apoAI-mediated cholesterol efflux, expressed as %, was calculated as the amount of label released to the medium after adding apoAI (the difference in radioactivity in the medium before and after adding ApoA1) divided by the amount of total label in each well (radioactivity released to the media plus radioactivity in the lysed cells).

### siRNA studies

siRNA pools specific for human OSBPL7, human CB1R and non target siRNA negative control are summarized in Supplementary Table 6. THP1 monocytes were seeded at a density of 80,000 cells/well in 96-well plates and differentiated with PMA as described above. After 72 h, medium was replaced by 50 µl of fresh medium per well. Cells were then transfected using Viromer Blue reagent (Origene) following the manufacturer’s directions. Briefly, 10 µl of siRNA (5 µM) was mixed with 90 µl of Viromer Blue (diluted 1% v/v in supplied buffer). After 15 min incubation, siRNA-transfection reagent complexes were diluted 1:6.5 in fresh medium and added in a volume of 50 µl per well. After 24 h, cells were then loaded with cholesterol by incubation with acetylated LDL (25 µg/ml) in RPMI medium, 1% FBS, 100 nM PMA, 2 µCi ^3^H-cholesterol for 24 h. A set of wells containing transfected cells was set aside to verify transfection efficiency by RT-PCR. The cholesterol-loaded macrophages were then incubated in the presence of Cpd G 5 μM, or vehicle (0.1% DMSO) in RPMI medium w/o Phenol Red, 0.2% BSA) for an additional 24 h. Cholesterol efflux was then measured as described above.

### RT-PCR

RNA was isolated using RNEasy Plus Mini kit (Qiagen). Reverse transcription was performed using qScript cDNA Super Mix (Quantabio) according to the manufacturer’s instructions. Quantitative real-time PCR was performed using TaqMan Fast Universal PCR Master Mix and Taqman gene expression assays (Supplementary Table 7).

### Western blot

Lysates, or cell fractions were incubated in Laemmli buffer at 55 °C for 10 min under reducing conditions. A total of 30 μg of protein from cell lysates or one-third of the volume collected in cell fraction preparations was separated by SDS-PAGE in 4–20% gradient gels (Biorad) and proteins were then transferred to PVDF membranes. After blocking with 5% TBS-milk, membranes were probed with primary antibodies overnight at 4 °C. The following primary antibodies were used: mouse anti-ABCA1 (Abcam; 1:1000), rabbit anti-GAPDH antibody (Millipore; 1:10,000), mouse anti-actin (CP01, Millipore 1:10,000), anti-MEK, anti-ERp72, anti-V5 and anti-Na^+^/K^+^ ATPase (Cell Signaling; all 1:1000), and rabbit anti-OSBPL7 (Sigma; 1:1000). After washing, membranes were incubated with anti-rabbit or and anti-mouse IgG-HRP antibodies (Promega, 1:10,000). Signals were detected after incubation with Westernbright ECL HRP substrate (Advansta) and luminescence signals were captured with Azure C600 Gel Imaging workstation (Azure Biosystems Inc, USA) or X-ray films.

### Cell fractionation

Differentiated podocytes were treated with compounds Cpd C (1 μM), Cpd A (5 μM), or Cpd G (10 μM) for 18 h, washed and then harvested in ice-cold PBS. Cells were centrifuged at 1000 × *g* for 5 min, the supernatant was carefully removed, and the pellet resuspended in hypotonic buffer (15 mM KCl, 1.5 mM MgCl_2_, 10 mM HEPES and 1 mM DTT) supplemented with a protease inhibitor cocktail (Roche). Cells were disrupted with a glass douncer, and sucrose was added to achieve final concentration of 227 mM. Cell lysates were centrifuged at 1000 × *g* for 30 min at 4 °C. The supernatant was then transferred to a new tube and centrifuged at 10,000 × *g* for 15 min at 4 °C. The pellet, containing the microsomal fraction was collected and the supernatant was transferred to a new tube and centrifuged at 100,000 × *g* for 1 h at 4 °C. The pelleted plasma membrane fraction was collected and the supernatant, containing the organelle-free cytosolic fraction, was concentrated in Vivaspin 500 filter columns. Western blot was performed using antibodies for ABCA1 and specific markers for each membrane fraction as described above. Na^+^/K^+^ ATPase was used to identify the plasma membrane fraction, ERp72 as a marker for the microsomal-enriched fraction, and MEK as the control for the non-membranous cytosolic fraction.

### ABCA1 co-immunoprecipitation

ABCA1-Flag construct was kindly provided by Dr. Michael Fitzgerald, Harvard University. OSBPL7-V5 construct (pcDNA3.1-V5-ORP7) was purchased from Addgene. Mammalian expression vectors PCMV6-AN-GFP and pCMV6-AC-3DDK, from Origene, were used as negative controls. HEK293 cells were transiently co-transfected with either one of the following plasmid combinations: (1) ABCA1-Flag + OSBPL7-V5; (2) ABCA1-Flag + PCMV6-AN-GFP; or (3) OSBPL7-V5 + pCMV6-AC-3DDK using Fugene 6 transfection reagent (Promega) following the manufacturer’s instructions. 48 h after transfection, cells were lysed with 1 ml immunoprecipitation buffer (1% Triton X-100, 50 mM Tris-Cl pH 7.0, 150 mM NaCl) supplemented with protease inhibitor cocktail (Roche). The lysates were cleared by centrifugation and equal amounts of total proteins from each supernatant were incubated overnight at 4 °C with 30 µl of anti-Flag M2 Affinity gel (Sigma Aldrich). The beads were pelleted by centrifugation and washed 5 times with 1 ml of immunoprecipitation buffer. Immunoprecipitated proteins were eluted by incubating the beads with 50 µl of 2× sample buffer for 15 min at 56 °C. The presence of ABCA1, OSBPL7-V5 in the lysates and eluates was verified by western blot as described above.

### Animal studies

Animals were housed on 12 h/12 h light/dark cycles and under controlled temperature (18–23 °C) and humidity (40–60%) in a pathogen-free animal facility of the Division of Veterinary Resources at the University of Miami, Miller School of Medicine. Mice were provided a standard 18% protein rodent chow diet and water *ad libitum*. The study protocol was approved by the Animal Care and Use Committee (IACUC) of the University of Miami, which is accredited by the Association for Assessment and Accreditation of Laboratory Animal Care International (AAALAC). All procedures were performed in compliance with all federal and state ethical regulations, including the Animal Welfare Act (AWA) regulations overseen by the United States Department of Agriculture (USDA) and the Public Health Service Policy on Humane Care and Use of Laboratory Animals (PHS Policy) administered by the National Institutes of Health (NIH), Office of Laboratory Animal Welfare (OLAW). Female Balb/c mice were purchased from the Jackson Laboratories for Adriamycin-induced nephropathy studies. At 8 weeks of age, mice received a single dose of Adriamycin (Sigma-Aldrich, 12 mg/Kg) or vehicle (0.9% NaCl) via tail vein injection. Mice were randomly separated into six experimental groups and, starting at 24 h post-ADR injection, treated with either vehicle or different doses of compounds by daily via oral gavage for 28 days. The following doses were used: Cpd C (LXR agonist) 10 mg/Kg; Cpd A 30 mg/Kg; Cpd G 100 mg/Kg. All animals were sacrificed 28 days post-ADR injection. Col4a3 KO mice (Col4a3^tm1Dec^ strain 129X1/SvJ) were purchased from Jackson Labs. Mice were randomly separated into 2 groups. Starting at 4 weeks of age, the mice were dosed by oral gavage once daily with either vehicle or Cpd G (100 mg/Kg) for 4 weeks followed by sacrifice at 8 weeks of age. A second study was performed in Col4a3 KO mice with treatment initiated at 6 weeks of age. This study was performed in order to assess if Cpd G treatment of mice with established renal failure might delay end stage renal disease and therefore prolong survival. For ramipril treatment, Col4a3 KO mice started receiving ramipril at the age of 4 weeks. Ramipril was added to the drinking water at a concentration that would lead to a dose of 10 mg/Kg/day^38^.

### Phenotypic analysis of mice

Weekly spot urine samples were collected. Urinary albumin was measured by ELISA (Bethyl laboratories) and creatinine by a colorimetric assay (Stanbio). Albuminuria was expressed as the albumin/creatinine ratio. Mice were anesthetized with Ketamine (90 mg/Kg)/Xylazine (10 mg/Kg), blood was collected in heparinized tubes, and animals were then perfused via the left ventricle with 0.9% NaCl solution. Kidney cortexes were carefully excised and separated into samples that were either snap frozen, embedded in OCT, or fixed in 10% formalin for subsequent assessments.

### Determination of lipid deposition in kidney cortex sections

OCT embedded kidney cortex samples were cryosectioned at 4 μm thickness and stained with Oil-Red O-isopropanol solution (Electron Microscopy Science, PA) diluted 6:4 in water, and then counterstained with Hematoxylin Harris Hg Free (VWR, PA). Sections were then evaluated using a light microscope (Olympus BX 41, Tokyo, Japan).

### Determination of cholesterol and triglyceride content

Snap frozen kidney cortex samples were homogenized in 2 mM potassium phosphate buffer, pH 7, (10 µl buffer/mg tissue) using a glass douncer on ice. 100 μl of the homogenate were mixed thoroughly with 1 ml of hexane:isopropanol (3:2;v:v) per volume of tissue homogenate. The aqueous (pellet) and organic (supernatant) phases were separated by centrifugation at 12,000 × *g* for 5 min. Pellets (aqueous phase) were dried, reconstituted in 8 M urea, 0.1% SDS, 0.1 M NaOH, and total proteins were quantified by the BCA method (Thermofisher). The organic phase was dried by exposure to nitrogen. Lipids were then reconstituted in 100 µl isopropanol:NP-40 (9:1;v:v). Triglyceride content was assayed using a colorimetric kit according to the manufacturer’s instructions (Cayman Chemical USA). Total cholesterol and CE content were assayed using an enzymatic fluorometric method^59^. For total cholesterol measurement, extracts were diluted 1:100 in assay buffer (100 mM potassium phosphate, 50 mM NaCl, 5 mM cholic acid, 0.1% Triton X-10, pH 7.4) containing cholesterol oxidase (1 U/ml), cholesterol esterase (1 U/ml), horseradish peroxidase (1 U/ml), and Amplex Red (75 µM). The reactions were incubated at 37 °C for 30 min in a black opaque 96-well plate (Greiner) and fluorescence was measured in a microplate reader (Spectramax i3X, Molecular Devices) at 530 nm excitation and 580 emission.

For CE determination, 150 µl of the assay buffer described above, supplemented with bovine liver catalase (45 U/ml) and cholesterol oxidase (1 U/ml), was added to 25 μl of sample and incubated overnight at 37 °C to deplete free cholesterol leaving only cholesterol esters. Then, 75 µl of cholesterol ester detection reagent (1 U/ml cholesterol oxidase, 4 U/ml cholesterol esterase, 24 U/ml horseradish peroxidase, 300 µM Amplex Red) was added. The reaction was incubated at 37 °C for 30 min and the fluorescence signal was measured as described above. Internal standards included samples containing 1 mM of cholesterol and 5 μM of cholesterol oleate in order to verify the completeness of the enzymatic decomposition of free cholesterol as well as assay sensitivity and specificity.

### Histological evaluation

Formalin-fixed paraffin-embedded kidney cortex sections 4 μm thick were stained with Periodic acid-Schiff (PAS). The slides were analyzed by a renal pathologist in a double-blind design. Glomerular sclerosis, (global and segmental), podocyte hypertrophy and podocyte hyperplasia were expressed as the percentage of glomeruli found with these abnormalities. Tubular microcysts and interstitial inflammation were scored on a 0–4 scale where 0 = 0%, 0.5 = 1–10%, 1 = 11–25%, 2: 26–50 %, 3: 51–75%, and 4 = more than 75% damage. Mesangial expansion was scored based on semi-quantitative analysis (scale 0–5) or percent of glomeruli exhibiting mesangial expansion (%).

### Immunofluorescence microscopy

For the detection of ABCA1 expression, 4 μm paraffin kidney cortex sections were deparaffinized and rehydrated. Antigens were retrieved by boiling for 30 min in citrate buffer pH 6.0 (Target Retrieval Solution, Citrate pH 6, Sigma, USA). The sections were permeabilized for 30 min with 0.1% Triton X-100 in TBS. Tissue sections were incubated with 10% goat serum (cat # G9023, Millipore Sigma) and 1% BSA (cat # SP-5050-500, Vector, USA) in TBS-T for 1hr at room temperature to block any non-specific binding. Samples were then incubated overnight at 4 °C with Rat anti-ABCA1 (Novus, 1:200) and Rabbit anti-WT1 (Abcam, 1:200) antibodies in a humidified chamber. After washing extensively, samples were then incubated for 1 h at RT with fluorochrome-conjugated secondary antibodies (Invitrogen, 1:500). Antifade mounting media containing DAPI (Vectashield Plus) was added to the sections before being analyzed.

Images were acquired using a FluoView FV1000 Confocal Microscope (Olympus) with a ×63 oil objective lens in different planes using a Z-series pattern with a step size of 0.18 μm. During analysis, individual planes were deconvoluted and stacked to produce a maximum projected image using fluview software version 4.3b (Olympus). A minimum of 5 images was captured from 5 different fields per sample. Quantification of ABCA1 glomerular mean fluorescence intensity (MFI) was done using Fiji—ImageJ software.

For the detection of OSBPL7, 4 μm frozen kidney cortex sections embedded in OCT, were fixed with PFA-sucrose (2%, 4%) in PBS for 20 min at RT and permeabilized with 0.3% Triton-PBS for 15 min and blocked with blocking buffer (5% FBS in PBS) for 1 h. Tissue sections were then incubated with rabbit anti-OSBPL7 (Sigma, 1:50) and Goat anti-Synaptopodin (Santa Cruz, 1:800) antibodies in a humidified chamber overnight at 4 °C. After washing extensively, samples were then incubated with fluorochrome-conjugated secondary antibodies, and covered with mounting media as described above. Images were captured using a Leica DMI 6000B fluorescent microscope.

### Statistical analysis

GraphPad Prism ver 6 or 7 was used to perform all statistical analysis. Groups were compared using two-tailed *t* test or One-way ANOVA followed by Dunnett’s or Tukey’s test. F, Brown-Forsythe, or Bartlett’s test were used to determine significant variances between the groups, if *P* < 0.05, then groups were compared using two-tailed Mann–Whitney, or Kruskal–Wallis followed by Dunn’s tests. Correlation between albuminuria and accumulation of cholesterol ester in kidney cortexes was assessed using Spearman coefficient test. *P* values < 0.05 were considered statistically significant. Only data from independent experiments were analyzed.

All animals were randomly assigned to treatment groups.

### Reporting summary

Further information on research design is available in the Nature Research Reporting Summary linked to this article.

## Supplementary information

Supplementary Information

Description of Additional Supplementary Files

Supplementary Data 1

Reporting Summary

## Data Availability

The authors declare that the data supporting the findings of this study are available within the paper and its Supplementary information files. The data generated in this study are provided in the Supplementary Information and the Source Data file. Source data are provided with this paper.

## Source data

Source data
