# Supplementary material for: Compounds targeting OSBPL7 increase ABCA1-dependent cholesterol efflux preserving kidney function in two models of kidney disease
Source: Nat Commun. 2021 Aug 2;12:4662. doi: 10.1038/s41467-021-24890-3 (PMC8329197; doi:10.1038/s41467-021-24890-3)
Supplement: Supplementary file 1 — Supplementary Information [file 41467_2021_24890_MOESM1_ESM.pdf]

SUPPLEMENTARY INFORMATION.

- **Supplementary Fig. 1. Structures of compounds used in this study**
- **Supplementary Table 1. Potency for binding to the CB1R *in vitro***
- **Supplementary Fig. 2. Full Western blot for OSBPL7 and autoradiography for Cpd A and Cpd G shown in Fig 2d.**
- **Supplementary Fig. 3. Full Western blot for OSBPL7 and autoradiography for compound K binding to OSBPL mutants shown in Fig 3b.**
- **Supplementary Fig. 4. Full Wb for ABCA1 and GAPDH expression in podocytes after treatment with Cpd A and Cpd G shown in Fig. 4a.**
- **Supplementary Fig. 5. Full Western blots for cell fractions shown in Fig. 4c,d.**
- **Supplementary Fig. 6. Full Western blot to detect ABCA1-Flag and V5-OSBPL7 proteins in co-immuno precipitation experiments.**
- **Supplementary Table 2. DMPK studies**
- **Supplementary Fig. 7. Full Western blot showing endogenous OSBPL7 expression in human kidney and cells shown in Fig 4e.**
- **Supplementary Fig. 8. Cpd A and Cpd G attenuate nephropathy induced by ADR challenge.**
- **Supplementary Fig. 9. Cpd G prevented ADR-induced podocyte loss and downregulation of ABCA1 protein expression in glomeruli**.
- **Supplementary Fig. 10. Effect of Cpd G on IL-1β and MCP-1 expression in kidney cortexes after ADR challenge**.

- **Supplementary Fig. 11. Effect of compound treatment on cholesterol and triglycerides in serum, kidney and liver after ADR challenge.**
- **Supplementary Table 3. Serum cholesterol and triglycerides in animals treated with Cpd A, Cpd G and Cpd C after ADR injection.**
- **Supplementary Table 4. Effect of CpdA and Cpd G treatment after ADR injection on Hemoglobin, White blood cell count, AST and ALT transaminases**
- **Supplementary Fig. 12. Cpd G protective effect in Col4a3 KO mice was not inferior to pre-emptive treatment with ramipril.**
- **Supplementary Fig. 13. Effect of Cpd G on cholesterol and Triglycerides in sera and kidney from Col4a3KO mice**
- **Supplementary Table 5. List of compounds used in this study.**
- **Supplementary Table 6. siRNA pools used for silencing OSBPL7 and CB1R in THP1 cells.**
- **Supplementary Table 7. Probes used for RT-PCR to quantify mRNA expression.**

Supplementary Fig. 1

Structures of compounds used in this study

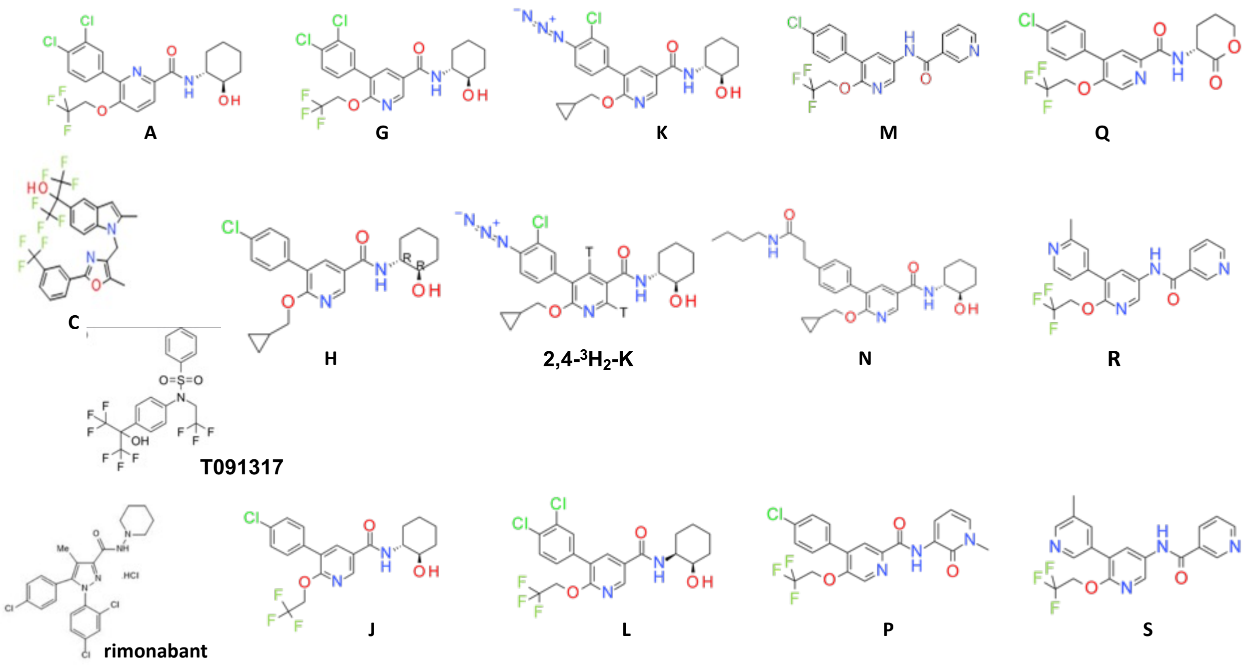

Supplementary Table 1

Potency for binding to the CB1R *in vitro*

Compound	hCB1-R *K*i [nM]
Rimonabant	2.7
A	>10000
G	1320
H	11.4 ± 4.5
J	12.4 ± 6.7
K	2570
L	7000
M	>10000

Table summarizing Ki binding of Compounds to human cannabinoid 1 receptor (hCB1-R).

Supplementary Fig. 2

Full Western blot for OSBPL7 and autoradiography for Cpd A and Cpd G shown in Fig 2d.

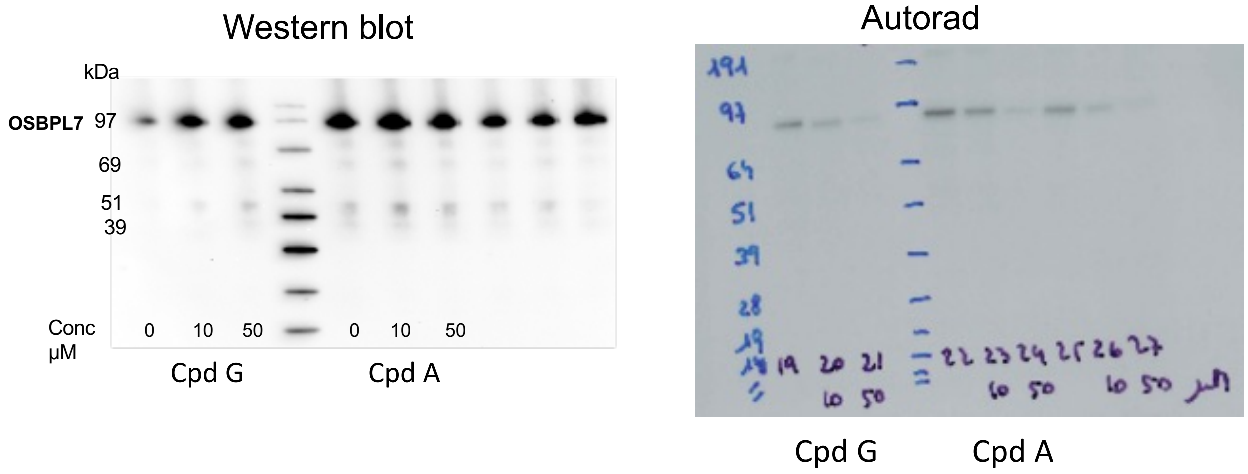

Competition binding activities of Cpd G and Cpd A that were selected for studies in podocytes and kidney disease models. Data representative from 3 independent experiments.

OSBPL7 antibody used (rabbit polyclonal antibody Sigma, Cat # HPA036076. 1: 1,000 dilution)

Supplementary Fig. 3.

Full Western blot for OSBPL7 and autoradiography for compound K binding to OSBPL mutants shown in Fig 3b.

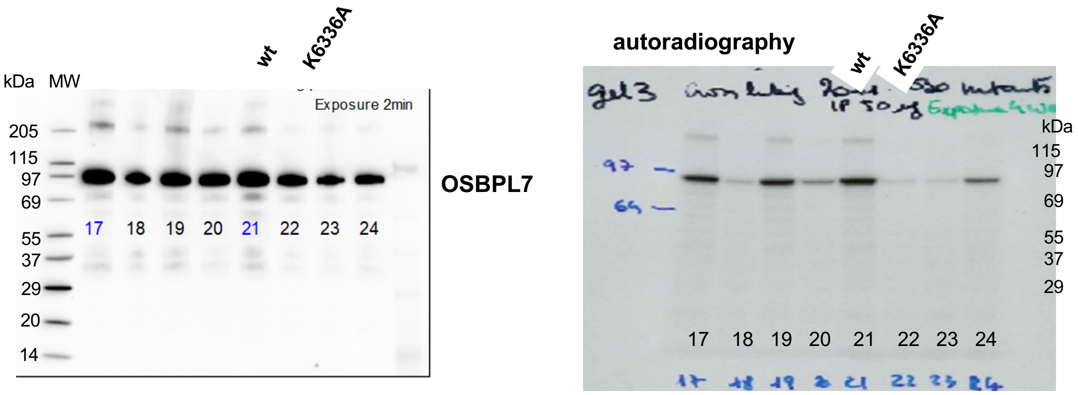

Evaluation of ^{3}H Cpd K binding to wildtype OSBPL7 (wt) and various single mutations introduced into the putative ORD.

OSBPL7 antibody used (rabbit polyclonal antibody Sigma, Cat # HPA036076. 1: 1,000 dilution)

Supplementary Fig. 4

Fig. S4. Full Wb for ABCA1 and GAPDH expression in podocytes after treatment with Cpd A and Cpd G shown in Fig. 4a.

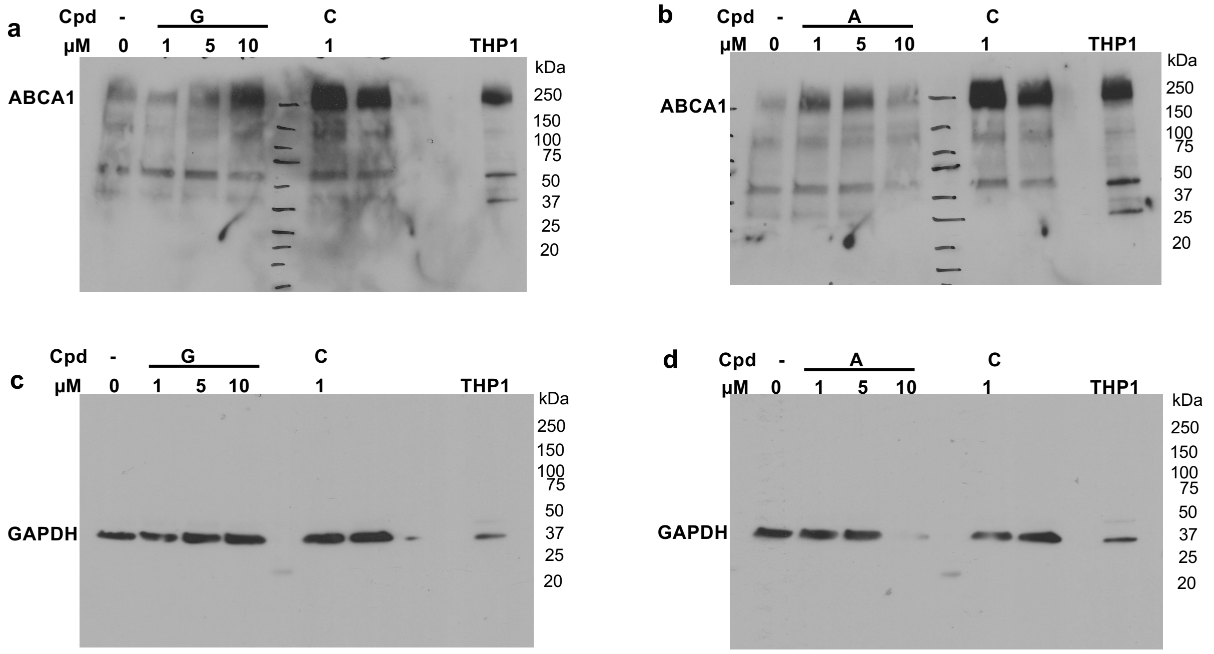

Representative ABCA1 and GAPDH Western blots from podocytes treated with Cpd G (**left**), Cpd A (**right**) and the LXR agonist Cpd C, at the concentrations indicated.

ABCA1 mouse monoclonal antibody (clone AB.H10, ABCAM Cat # ab18180). 1: 1,000

GAPDH antibody used (mouse monoclonal antibody 6C5, Millipore Cat # CB1001 diluted 1: 10,000

Supplementary Fig. 5

Full Western blots for cell fractions shown in Fig. 4c,d.

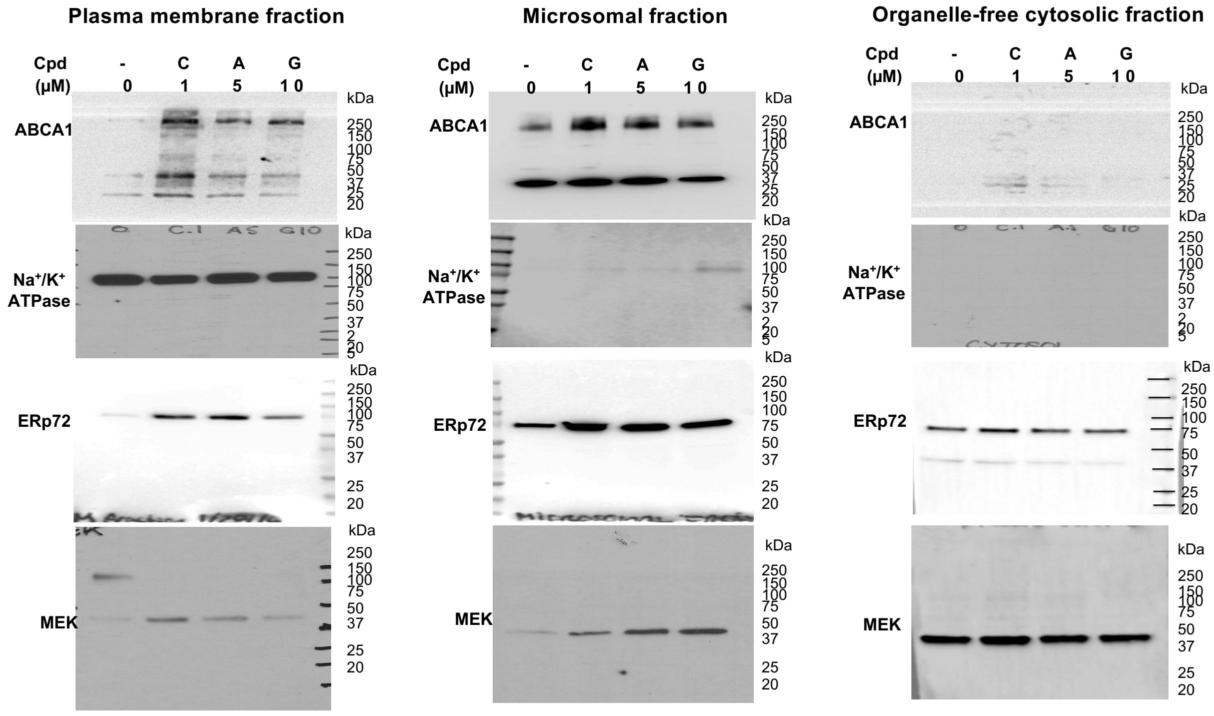

Representative Western blots for ABCA1, Na^+/K^+ ATPase and ERp72 in plasma membrane and microsomal enriched fractions from podocytes treated with Cpd C (1 μM), Cpd A (5 μM) or Cpd G (10 μM).

ABCA1 mouse monoclonal antibody (clone AB.H10, ABCAM Cat # ab18180).

Na+/K+ ATPase (rabbit mAb D4Y7E, Cat#23565), ERp72 (rabbit mAb D70D12, Cat# 5033) and MEK (L38C12, rabbit polyclonal antibody Cat# 4694). Cell Signaling. All antibodies used 1:1,000.

Supplementary Fig. 6

Full Western blot to detect ABCA1-Flag and V5-OSBPL7 proteins in co-inmmuno precipitation experiments.

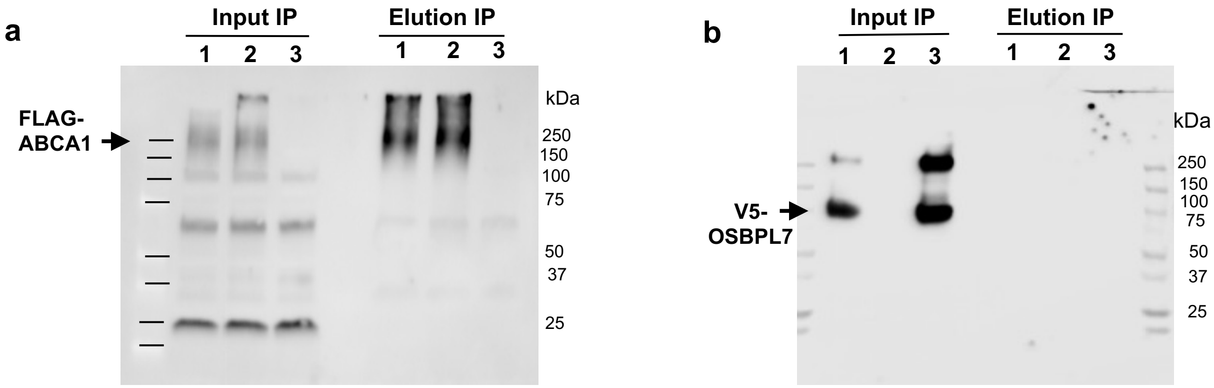

HEK293 cells were co-transfected with either one of following plasmid pair combinations: 1) FLAG-ABCA1+ OSBPL7-V5; 2) FLAG-ABCA1 + empty vector; or 3) FLAG empty vector + OSBPL7-V5. FLAG-tagged proteins were pulled down and the presence for ABCA1 or the V5 epitope fused to OSBPL7, was verified before (input IP) and after immunoprecipitation (elution IP). **a,b** Representative Western blots from 3 independent experiments. **a** Western blot for ABCA1, **b** Western blot for V5 epitope.

Supplementary Fig. 7

Full Western blot showing endogenous OSBPL7 expression in human kidney and cells shown in Fig 4e.

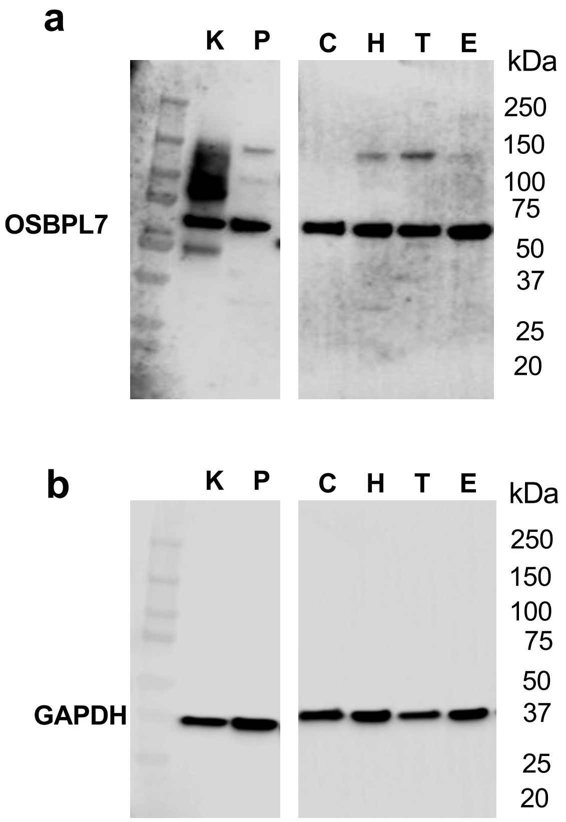

Representative Western blot showing OSBPL7 and GAPDH expression from human kidney (K) and podocytes (P). Total proteins from Caco2 (C), HepG2 (H), T (THP1) and HEK293 (H) were also analyzed.

OSBPL7 antibody used (rabbit polyclonal antibody Sigma, Cat # HPA036076. 1: 1,000 dilution). GAPDH antibody used (mouse monoclonal antibody 6C5, Millipore Cat # CB1001. 1: 10,000 dilution).

Supplementary Table 2

Supplementary Table S2. DMPK studies

DMPK in vitro	Cpd G	DMPK microsomal metabolism and PK *in vivo* - rat	Cpd G
CYPs (HLM.) [µM]*	>25 µM**	Microsomes (MAB%) (r/h)	70 / 82
TDI (3A4) time dependent inhib	Negative	Hepatocytes Clint (Fh%) (r/h)	70 / >80
CYP Induction	Negative	free fraction rat / man [%]	<1% both
free fraction rat / man [%]	<1% all	Cl [mL/min/kg]	2.5
PgP inhibition	Negative	Vss [L/kg]	1.5
GSH adduct	Negative	$t_{1/2}$ [h]	8.9
Covalent Binding	No flag	F [%]	105

Supplementary Fig. 8

Cpd A and Cpd G attenuate nephropathy induced by ADR challenge.

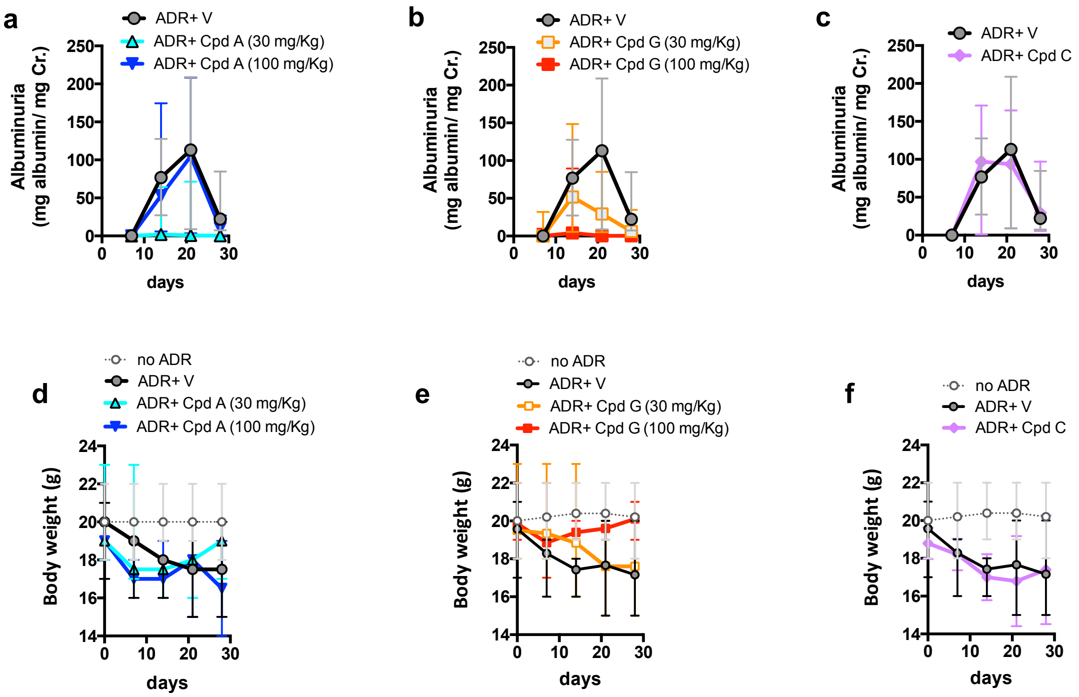

Treatment outcomes from mice that received vehicle (V, n=5); LXR agonist Cpd C (10 mg/Kg, n=5); Cpd A (30 mg/Kg, n=5 or 100 mg/Kg, n=5) or Cpd G (30 mg/Kg, or 100 mg/Kg, n=5) once daily starting a day after ADR injection. A group that did not receive ADR (no ADR, n=5) was included as reference of healthy phenotype. **a-c** Time course albumin to creatinine ratios (ACR) in spot urines from untreated mice (no ADR), ADR-injected mice that received V, Cpd A (**a**), Cpd G (**b)**, or Cpd C (**c**). Data expressed as the median and range of each treatment group. **d-f** Time course body weight of untreated mice (no ADR), ADR injected mice that received V, Cpd A (**d**), Cpd G (**e**) or Cpd C (**f**). Data expressed as the median and range of each treatment group.

Supplementary Fig. 9.

Cpd G prevented ADR-induced podocyte loss and downregulation of ABCA1 protein expression in glomeruli.

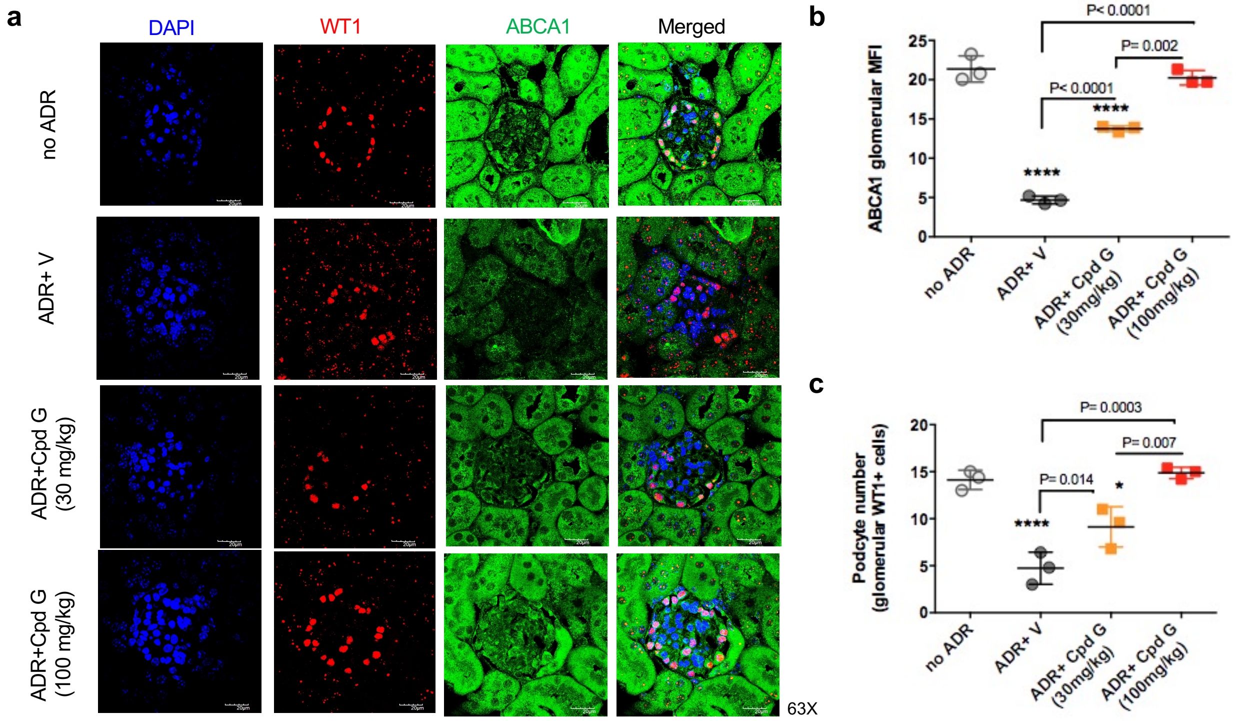

Kidney sections from animals that received vehicle or two doses of Cpd G (30 mg/Kg/day and 100 mg/Kg/day) after ADR injection were stained for ABCA1 and WT1, (a nuclear factor mostly expressed in podocytes). Sections from animals that did not receive ADR injection (no ADR) were also included as a reference of the healthy phenotype. Images from 5 different fields were captured per slide (1 slide per mouse, n= 3 mice per group). **b** ABCA1 glomerular mean florescence intensity. Data expressed as the mean ± SD. The groups were compared one to another using a one-way ANOVA followed by Tukey's test: $F(3, 8)=176.5$. * Differences vs (no

ADR): ADR+ V and ADR+ Cpd G (30 mg/Kg), ****P< 0.0001; ADR+ Cpd G (100 mg/Kg), P=0.55. Differences vs (ADR+ V): ADR+ Cpd G, both doses, P< 0.0001. ADR+ Cpd G (30 mg/Kg) vs ADR+ CpdG (100 mg/Kg): P= 0.0002. **c** Number of podocytes (WT1+ cells in the glomeruli) in the images captured. Data expressed as the mean ± SD. The groups that received ADR injection were compared one to another using one-way ANOVA followed by Tukey's test, F (3, 8)= 30.2. Differences with (no ADR): ADR+ V, *** P= 0.0003; ADR+ Cpd G (30 mg/Kg), *P=0.014; ADR+ Cpd G (100 mg/Kg), P= 0.93. Differences with (ADR+ V): ADR+ Cpd G (30 mg/Kg), P= 0.03; ADR+ Cpd 1(00 mg/Kg), P= 0.0002. ADR+ Cpd G (30 mg/Kg) vs ADR+ Cpd (100 mg/Kg), P= 0.007.

Supplementary Fig. 10.

Effect of Cpd G on IL-1β and MCP-1 expression in kidney cortexes after ADR challenge.

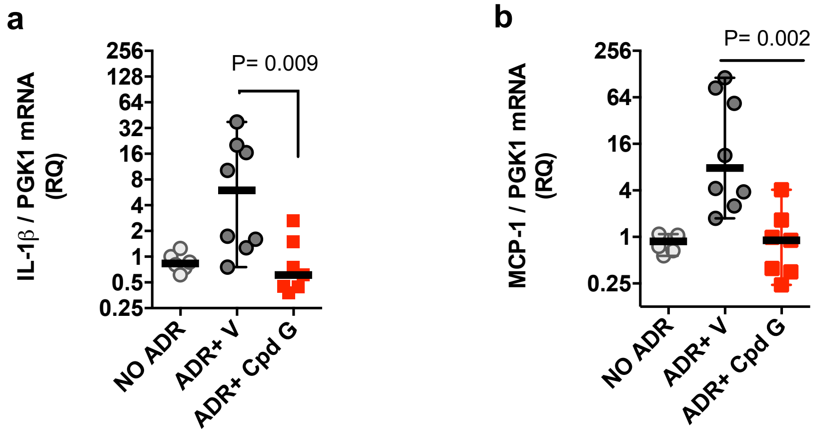

Quantification of IL-1β and MCP-1 mRNA expression in kidney cortexes from mice injected with ADR that received vehicle (ADR+ V, n=8) or Cpd G (ADR+ Cpd G, n= 7). The group that did not receive ADR (no ADR, n=6) was used as a reference basal control. Data expressed as median (horizontal lines) and range of the expression relative to the basal control (no ADR). Differences between the groups that received vehicle or Cpd G after ADR injection were calculated using two-tailed Mann Whitney test. **a** IL-1β /PGK1 mRNA relative expression (U=6, P= 0.009). **b** MCP-1/ PGK1 MRNA relative expression (U= 3, P= 0.002)

Supplementary Fig. 13.

Effect of compound treatment on cholesterol and triglycerides in serum, kidney and liver after ADR challenge.

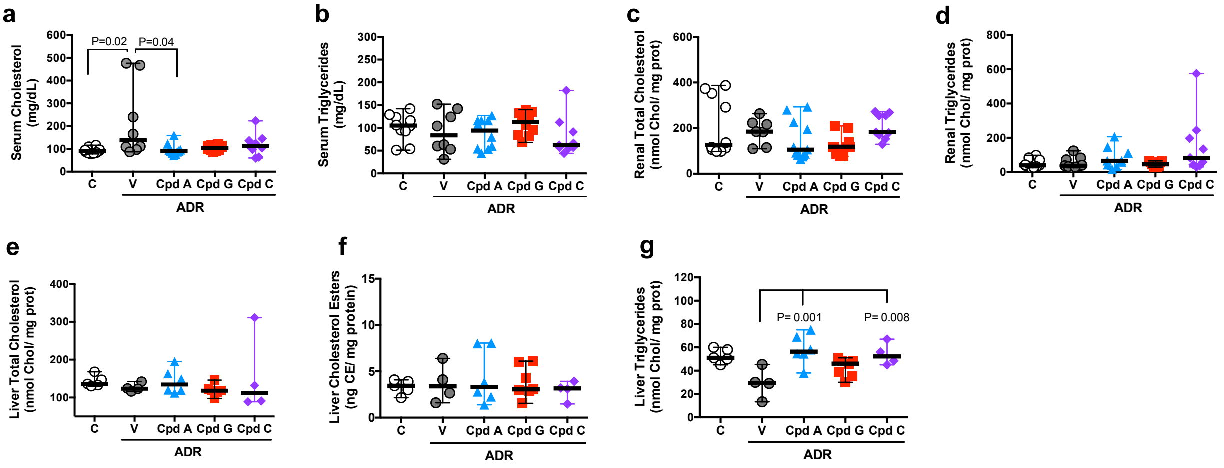

a-g Lipid levels in serum, kidneys and livers from animals treated with vehicle (V), Cpd A, Cpd G or Cpd C after ADR injection. A group that did not receive ADR injection (C, n=5) is included to reflect the healthy phenotype. **a** Serum cholesterol; **b** Serum triglycerides; **c** Total cholesterol in kidney; **d** Triglycerides in kidney; **e** Total cholesterol in liver; **f** Cholesterol esters (CE) in liver, **g** Triglycerides in liver. Data expressed as median and range. **a-d** Sample size: ADR+ V (n=8); ADR+ Cpd A (n=10); ADR+ Cpd G (n=10); ADR+ Cpd C (n=8). All groups were compared using a Kruskal-Wallis test ($P< 0.05$ only for plasma cholesterol). Differences with (ADR+ V) were calculated with Dunn's post hoc test: (no ADR , $P= 0.028$); (ADR+ Cpd A, $P= 0.004$); (ADR+ Cpd G, $P> 0.5$); (ADR+ Cpd C, $P> 0.5$) **e-g** Sample size: ADR+ V (n=4); ADR+ Cpd A (n=6); ADR+ Cpd G (n=6); ADR+ Cpd C (n=4). Differences between groups were calculated using one-way ANOVA: $F(3, 17)= 6.6$. $P< 0.05$ only for liver triglycerides.

Differences vs (ADR+ V) calculated using Dunn's test: ** (no ADR, $P = 0.01$); (ADR+ Cpd A, $P=0.001$); (ADR+ Cpd G, $P = 0.19$); (ADR+ Cpd C, $P = 0.01$). For total liver cholesterol, treatments compared using one-way ANOVA: $F(4, 20) = 0.5$, $P = 0.73$. For esterified cholesterol, treatments compared using one-way ANOVA: $F(4, 20) = 0.4$, $P = 0.79$.

Supplementary Table 3

Effect of compound treatment on cholesterol and triglyceride levels after ADR challenge

	NO ADR	ADR+ V	ADR+ Cpd A	ADR+ Cpd G	ADR+ Cpd C
**Cholesterol (mg/dL)**					
Range,<br>Median(IQR)	78-116,<br>90(82-99)	87-476,<br>138(100-410)[a]	71-159,<br>91(80-159)[b]	85-121,<br>104(93-115)	60-223,<br>112(81-141)
% of the group> 112 mg/dL	0 %	50 %	10 %	0 %	30 %
**Triglycerides (mg/dL)**					
Range,<br>Median(IQR)	51-142,<br>105(83-125)	31-152,<br>84(55-138)	44-127,<br>94(54-116)	68-140,<br>113(84-133)	44-182,<br>62(56-102)
% of the group> 112 mg/dL	0 %	50 %	10 %	0 %	30 %

Data expressed as range, median and interquartile range (IQR) within each group. Data in italics represent the percentage of animals within each group with values higher than the 95th percentile range of the healthy group, (112 mg/dl for cholesterol and 150 mg/dL for triglycerides). Groups were compared using Kruskal-Wallis test. $P < 0.05$ for cholesterol levels and $P > 0.05$ for triglycerides. Differences in blood cholesterol vs (ADR+ V) were calculated using Dunn's post hoc test: no ADR, ([a] P= 0.02); ADR+ Cpd A, ([b] P= 0.04); ADR+ Cpd G (P= 0.74); ADR+ Cpd C, (P> 0.99).

Supplementary Table 4.

Effects of compound treatment on white blood cells counts (WBC), hemoglobin (Hgb), hematocrit (HTC), ALT, and AST transaminase levels in ADR-induced nephropathy.

	WBC ($x10^3$ cells/μL)	Hgb (mg/dL)	HTC (%)	ALT (U/L)	AST (U/L)
**No ADR**					
Range	3.9-9.1,	12.2-14.9,	45-59,	31-158,	96-176,
Median (IQR)	6.5 (4.1-8.5)	12.8 (12.4-13.7)	50 (48-53)	61 (41-114	108 (98-147)
**ADR+ V**					
Range	3.5-12.3,	7.6-15.3,	30-61,	120-656,	282-478,
Median (IQR)	7.0 (3.5-12.3)[a]	13.8 (12.2-14.8)	52 (47-59)	407 (183-602)[a]	372 (284-472)[a]
**ADR+ Cpd A**					
Range	3.7-7.0,	12.1-15.5,	47-62,	88-766,	200-685
Median (IQR)	4.0 (3.8-5.3	13.6 (13-14.1)	54 (50-56)	256 (90.3-625)	326 (213-500)
**ADR+ Cpd G**					
Range	2.5-6.5,	12.2-13.7,	46-57,	90-317,	124-401,
Median (IQR)	5.2 (3.4-6.5)	13 (12.5-13.5)	51 (48-52)	213 (105-239)	213 (156-220)[b]
**ADR+ Cpd C**					
Range	5.4-8.3,	11-15.9,	43-65,	68-838,	90-433,
Median (IQR)	6.2 (5.5-7.9)	13.3 (12.2-15.5)	55 (45-64)	146 (71-682)	165 (104-371)

Data expressed as range (1st line), and median (interquartile range) (2nd line), of each parameter within each group. Sample size for WBC, Hgb and HTC: no ADR (n=10); ADR+ V (n=8); ADR+ Cpd A (n=10); ADR+ Cpd G (n=10); ADR+ Cpd (n=8). Sample size for ALT and AST: no ADR (n=5); ADR+ V (n=4); ADR+ Cpd A (n=6); ADR+ Cpd G (n=7); ADR+ Cpd C (n=4). Differences between (no ADR) and (ADR+ V) were calculated using with a two-tailed Mann-Whitney test. [a] $P< 0.05$ only for ALT ($P= 0.032$) and AST ($P=0.03$). Groups that received compound after ADR injection were compared to those that received vehicle (ADR+V) using a two-tailed Mann-Whitney test. For AST, [b] $P< 0.05$ only for (ADR+V) vs (ADR+ Cpd G), $U=2$, $P= 0.02$). For ALT, any group vs ADR+V, $P> 0.05$.

Supplementary Fig. 14.

Cpd G protective effect in Col4a3 KO mice was not inferior to pre-emptive treatment with ramipril.

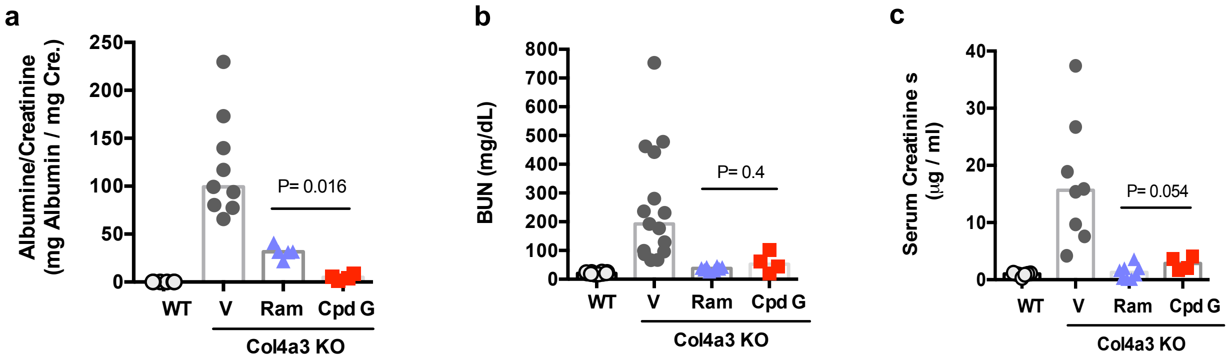

Treatment outcomes in 8-week-old Col4a3KO mice after receiving vehicle (V), ramipril (Ram) or CpdG for 28 days starting at the age of 4 weeks. Col4a3+/+ (WT) group included as reference of the healthy phenotype. Data expressed as median and range. **a** Albumin to creatinine ratio in spot urine samples. Ram (n= 5) vs Cpd G (n=4) treatments were compared using double-tailed Mann Whitney test: U=0, P= 0.016. **b** Blood urea nitrogen (BUN) levels. Ram (n=8) vs CpdG (n=4) treatments compared using double-tailed Mann Whitney test: U=9, P= 0.4. **c** Serum creatinine levels. Ram (n=8) vs CpdG (n=4) treatments compared using double-tailed Mann Whitney test: U=4.5, P= 0.054

Supplementary Fig. 15.

Effect of Cpd G on cholesterol and Triglycerides in sera and kidney from Col4a3KO mice

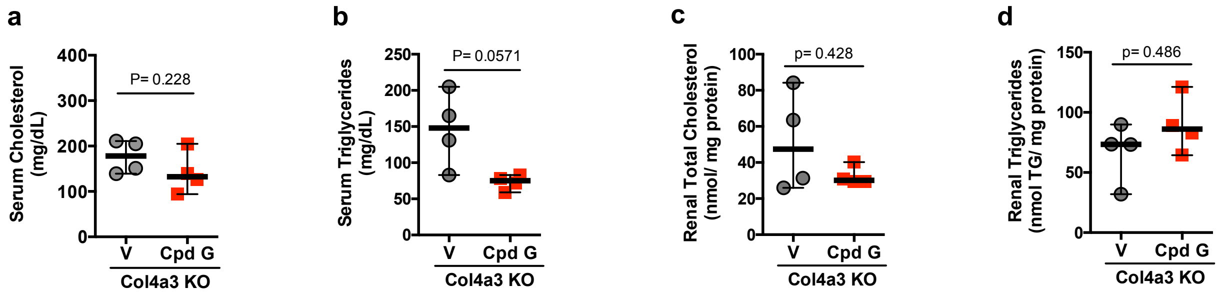

a-d Cholesterol and triglyceride levels in serum (**a,b**) and kidney (**c,d)** from 8-week old Col4A3 KO mice that received vehicle (n=4) or Cpd G (n=4) for 28 days. Data expressed as the median and range. Differences between the groups were calculated using a double tailed Mann Whitney test. **a** Serum cholesterol, P=0.228; **b** Serum triglycerides, P=0.057; **c** Total cholesterol in kidney cortex, P=0.428; **d** Triglycerides in kidney cortex, P= 0.486.

Supplementary Table 5.

List of compounds used in. this study.

LXR agonist	C	
LXR agonist	T1317	
ABCA1 inducer	A	
ABCA1 inducer	G	
ABCA1 Inducer	H	
ABCA1 Inducer	J	
ABCA1 Inducer (Azide)	K	
ABCA1 Inducer	L	
ABCA1 Inducer	M	
Inactive 5-arylnicotinamide	N	
Rimonabant		
Inactive 5-arylnicotinamide	P	
Inactive 5-arylnicotinamide	Q	
Inactive 5-arylnicotinamide	R	
Inactive 5-arylnicotinamide	S	
AM 251		Cayman. No. 71670
WIN55 212-2 mesylate		Cayman No. 10009023
ACEA		Cayman No. 91054

All compounds were synthesized at F. Hoffmann-La Roche Ltd., Basel, Switzerland, except for AM 251, WIN55 212-2 mesylate and ACEA, which were purchased from Cayman Chemical USA.

Supplementary Table 6.

siRNA pools used for silencing OSBPL7 and CB1R in THP1 cells

Gene	siRNA pool
Human OSBPL7	ON TARGETplus siRNA plus, L009234-01-0005, Dharmacon
Human CB1R	ON TARGETplus siRNA plus, L-004711-00-005, Dharmacon
Negative control	Mission siRNA Universal Negative Control # 1, Sigma, SICO01

Supplementary Table 7.

Probes used for RT-PCR to quantify mRNA expression

Gene	Taqman Expression Assay	Sequence
Human ABCA1	Hs01059101_m1	not provided
Human OSBPL7	Hs01555054_m1	not provided
Human CB1R	Hs01038522_s1	not provided
Human GAPDH (endogenous control)	Hs99999905_m1	not provided
Mouse MCP-1	Mm00441242_m1	not provided
Mouse IL-1β	Mm00434228_m1	not provided
Mouse Phosphoglycerate Kinase (PGK1) (endogenous control)	Mm00435617_m1	not provided
