# Supplementary material for: Compounds targeting OSBPL7 increase ABCA1-dependent cholesterol efflux preserving kidney function in two models of kidney disease
Source: Nat Commun. 2021 Aug 2;12:4662. doi: 10.1038/s41467-021-24890-3 (PMC8329197; doi:10.1038/s41467-021-24890-3)
Supplement: Supplementary file 2 — Description of Additional Supplementary Files [file 41467_2021_24890_MOESM2_ESM.pdf]

Description of Additional Supplementary Files

File Name: Supplementary Data 1
Description: Synthetic Chemistry Procedures and Analytical Data
