# Supplementary material for: Compounds targeting OSBPL7 increase ABCA1-dependent cholesterol efflux preserving kidney function in two models of kidney disease
Source: Nat Commun. 2021 Aug 2;12:4662. doi: 10.1038/s41467-021-24890-3 (PMC8329197; doi:10.1038/s41467-021-24890-3)
Supplement: Supplementary file 3 — Supplementary Data 1 [file 41467_2021_24890_MOESM3_ESM.pdf]

# Compounds targeting OSBPL7 increase ABCA1-dependent cholesterol efflux preserving kidney function in FSGS and Alport Syndrome

## Supplementary Data: Synthetic Chemistry Procedures and Analytical Data

**General Information.** Reactions were conducted under argon atmosphere. Unless otherwise mentioned, all reagents and chemicals were obtained from commercial suppliers and used without further purification. Flash column chromatography was carried out on a Jones Chromatography Flashmaster II system using Telos Flash silica or Isolute FlashSi prepacked columns. Compounds were characterized by a combination of TLC, NMR, and MS analytical techniques as described below. $^{1}$H and $^{13}$C NMR spectra were recorded on a Bruker DRX 400, Bruker AVANCE II 400, or on a Bruker AVANCE III 600 spectrometer and were determined in $CHCl_3$-d or DMSO-d6 with tetramethylsilane (TMS) (0.00 ppm) as the internal reference unless otherwise noted. Chemical shifts are reported in ppm relative to TMS and coupling constants (J) are reported in hertz (Hz). Thin-layer chromatography (TLC) was performed on EMD precoated silica gel 60 F254 plates, and spots were visualized with UV light or chemical detection. Low-resolution mass spectra were obtained using an Applied Biosystem API300, and high-resolution LC-MS data were obtained using an Agilent Q-TOF 6520 system.

Actual synthesis of all compounds was done between 2006 and 2012 and compounds were originally characterized with MS and $^{1}$H NMR data only. Re-analysis for 13C NMR and some HRMS was done in 2018/19 with stored samples. Four compounds were out of stock and consequently only $^{1}$H NMR data are reported. All four were intermediates and all final compounds are characterized completely.

**5-(4-Chlorophenyl)-6-(cyclopropylmethoxy)-*N*-[(1*R*,2*R*)-2-hydroxycyclohexyl]- 3-pyridinecarboxamide (Cpd H)**

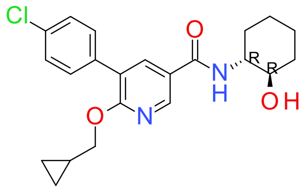

Synthesis and characterization as described in the literature.[1]

**5-(4-Chlorophenyl)-*N*-[(1*R*,2*R*)-2-hydroxycyclohexyl]-6-(2,2,2-trifluoroethoxy)- 3-pyridinecarboxamide (Cpd J)**

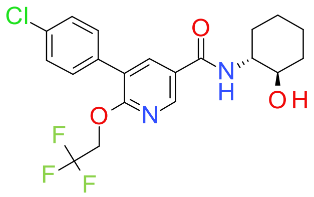

**Rimonabant**

Synthesis and characterization as described in the literature.[1]

Synthesis and characterization as described in the literature.[3]

**Synthesis of 5-[4-[3-(butylamino)-3-oxopropyl]phenyl]-6-(cyclopropylmethoxy)-*N*-[(1*R*,2*R*)-2-hydroxycyclohexyl]-3-pyridinecarboxamide (Cpd N)**

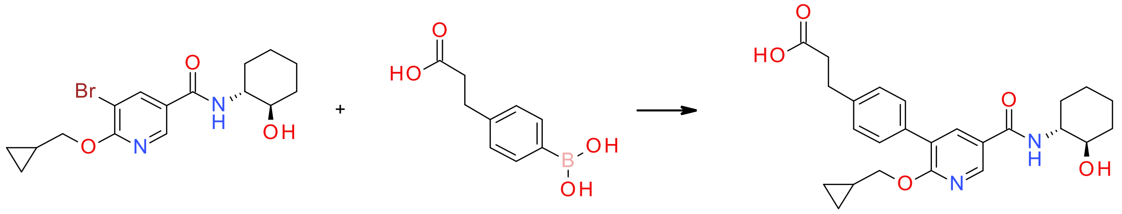

1 2 3

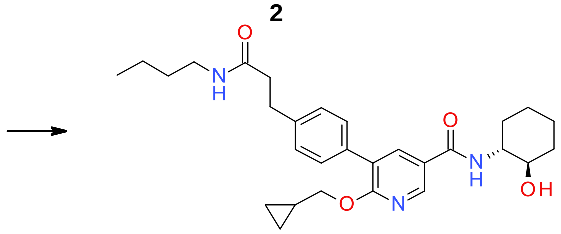

**Cpd N**

**5-Bromo-6-(cyclopropylmethoxy)-*N*-[(1*R*,2*R*)-2-hydroxycyclohexyl]-3-pyridinecarboxamide (1)**

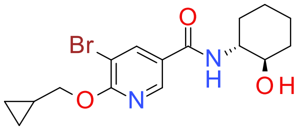

Synthesis and characterization as described in the literature.[1]

**3-{4-[2-Cyclopropylmethoxy-5-((1*R*,2*R*)-2-hydroxy-cyclohexylcarbamoyl)-pyridin-3-yl]-phenyl}-propionic acid (3)**

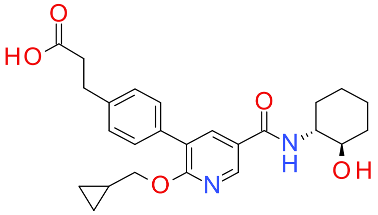

4-(2-Carboxyethyl)benzeneboronic acid (**2**, 158 mg, 0.8 mmol, ABCR L17485), [1,1'-bis(diphenylphosphino)ferrocene]dichloropalladium(II) x $CH_2Cl_2$ complex (1:1) (33 mg, 0.04 mmol) and sodium carbonate solution (820 µL, 2M, 1.6 mmol) was added to a solution of 5-bromo-6-(cyclopropylmethoxy)-*N*-[(1*R*,2*R*)-2-hydroxycyclohexyl]-3-pyridinecarboxamide (**1**, 300mg, 0.8 mmol) in toluene (12 mL)/DMF (1 mL) and stirred for 6 h at 85°C. Water (10 mL) and hydrochloric acid (5 mL, 1 N) was added and organic material was extracted into ethyl acetate. After drying with sodium sulfate and evaporation of solvent, the brown residue (0.56 g)

was purified by flash chromatography ($CH_2Cl_2$/MeOH gradient), followed by an additional extraction-wash cycle and flash chromatography to remove excess DMF, to finally afford the title compound as a white solid (0.24 g, 67%). $^{1}$H NMR (600 MHz, DMSO-d6): δ 12.14 (s, 1H), 8.58 (d, *J* = 2.4 Hz, 1H), 8.18 (d, *J* = 2.4 Hz, 1H), 8.16 (d, *J* = 8.2 Hz, 1H), 7.56 - 7.59 (m, 2H), 7.32 - 7.35 (m, 2H), 4.54 - 4.73 (m, 1H), 4.23 (d, *J* = 7.1 Hz, 2H), 3.56 - 3.69 (m, 1H), 3.37 - 3.45 (m, 1H), 2.88 (t, *J* = 7.6 Hz, 2H), 2.59 (t, *J* = 7.6 Hz, 2H), 1.90 (br d, *J* = 10.8 Hz, 1H), 1.81 - 1.86 (m, 1H), 1.60 - 1.69 (m, 2H), 1.17 - 1.29 (m, 5H), 0.50 - 0.54 (m, 2H), 0.31 - 0.35 (m, 2H). $^{13}$C NMR (151 MHz, DMSO-d6): δ 173.7, 164.2, 161.4, 145.7, 140.5, 137.5, 133.6, 129.0, 128.2, 124.4, 122.4, 71.1, 70.6, 55.2, 35.0, 34.4, 31.4, 30.0, 24.5, 24.2, 9.9, 3.1. HRMS calculated for $C_{25}H_{30}N_2O_5$ $[M+H]^+$ 439.2232, found 439.2226.

**$^{1}$H NMR of compound 3**

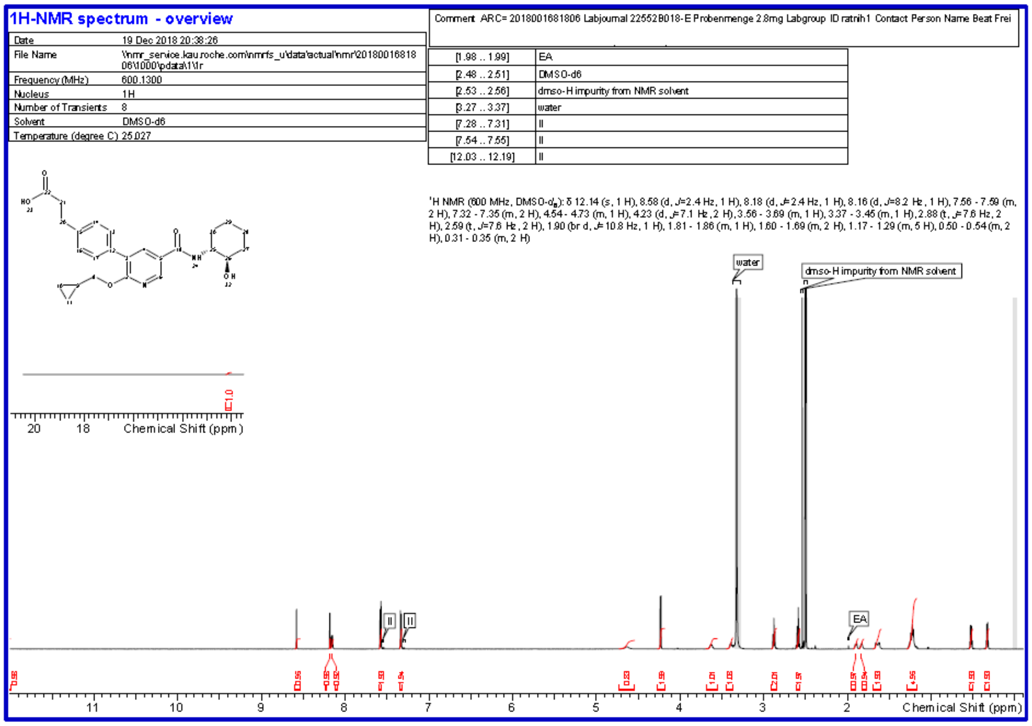


**$^{13}$C NMR of compound 3**

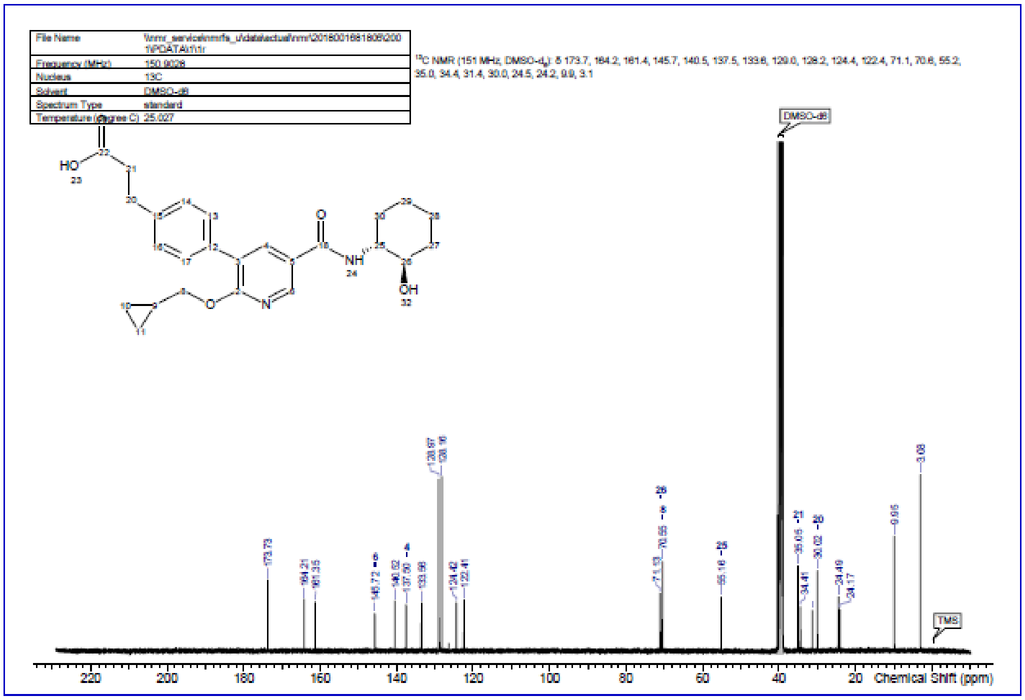


**Synthesis of 5-[4-[3-(butylamino)-3-oxopropyl]phenyl]-6-(cyclopropylmethoxy)-*N*-[(1*R*,2*R*)-2-hydroxycyclohexyl]-3-pyridinecarboxamide (Cpd N)**

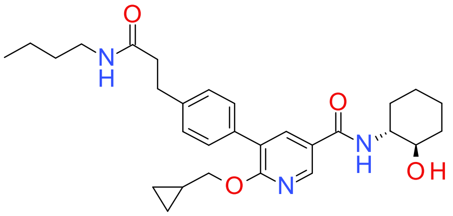

2-(1*H*-Benzotriazole-1-yl)-1,1,3,3-tetramethyluronium tetrafluoroborate (TBTU) (70 mg, 0.2 mmol), *N*,*N*-diisopropyl ethyl amine (185 µL, 1.1 mmol) and *N*-butylamine (21 µL, 0.2 mmol) was added to a solution of 3-{4-[2-cyclopropylmethoxy-5-((1*R*,2*R*)-2-hydroxy-cyclohexylcarbamoyl)-pyridin-3-yl]-phenyl}-propionic acid (**3**, 95 mg, 0.2 mmol) in DMF (3 mL) and stirred for 16 h at room temperature. The solvent was removed in high vacuum and the residue (0.28 g) was purified by flash chromatography ($CH_2Cl_2$/MeOH gradient) to give the title compound as a white foam (69 mg, 64%).$^{1}$H NMR (600 MHz, DMSO-*d*6) δ ppm 8.58 (d, *J* = 2.3 Hz, 1H), 8.17 (d, *J* = 2.3 Hz, 1H), 8.16 (d, *J* = 8.1 Hz, 1H), 7.79 (t, *J* = 5.8 Hz, 1H), 7.51 - 7.61 (m, 2H), 7.26 - 7.33 (m, 2H), 4.52 - 4.73 (m, 1H), 4.23 (d, *J* = 7.1 Hz, 2H), 3.58 - 3.68 (m, 1H), 3.37 - 3.42 (m, 1H), 3.03 (m, 2H), 2.86 (t, *J* = 8.3 Hz, 2H), 2.40 (t, *J* = 8.3 Hz, 2H), 1.87 - 1.93 (m, 1H), 1.79 - 1.86 (m, 1H), 1.57 - 1.73 (m, 2H), 1.30 - 1.39 (m, 2H), 1.17 - 1.28 (m, 7H), 0.84 (t, *J* = 7.4 Hz, 3H), 0.50 - 0.54 (m, 2H), 0.31 - 0.35 (m, 2H). $^{13}$C NMR (151 MHz, DMSO-d6): δ

171.0, 164.2, 161.3, 145.6, 141.0, 137.5, 133.4, 128.9, 128.1, 124.4, 122.4, 71.1, 70.5, 55.2, 38.1, 36.8, 34.4, 31.4, 31.2, 30.8, 24.5, 24.2, 19.5, 13.6, 9.9, 3.1. HRMS calculated for $C_{29}H_{39}N_3O_4$ $[M+H]^+$ 494.3019, found 494.3010.

### $^1$H NMR of compound N

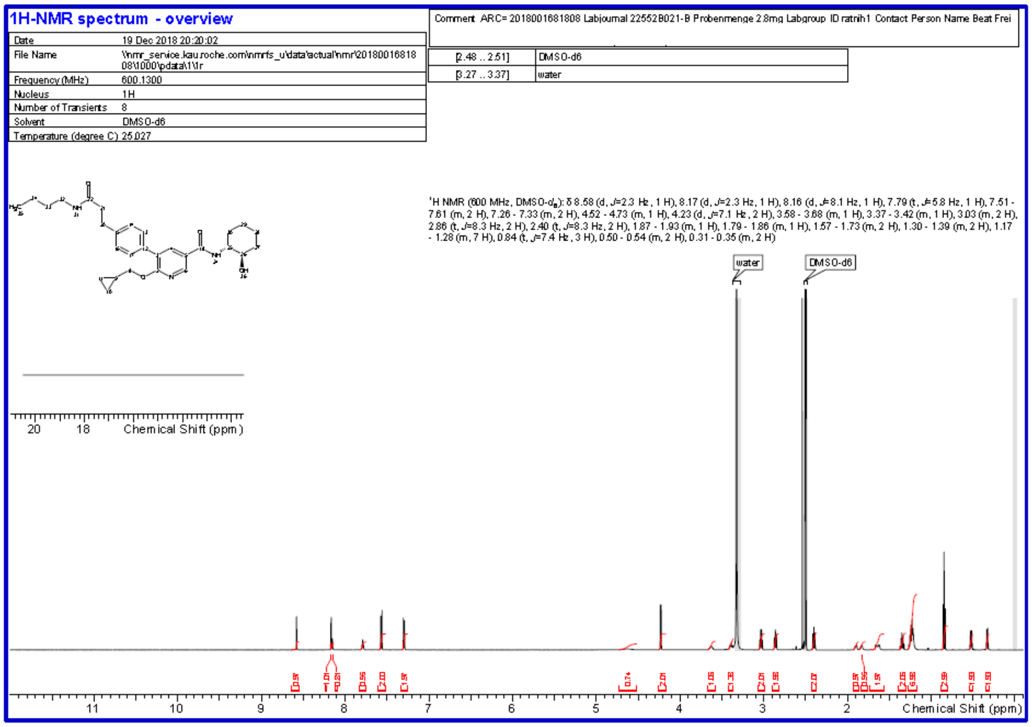

## $^{13}$C NMR of compound N

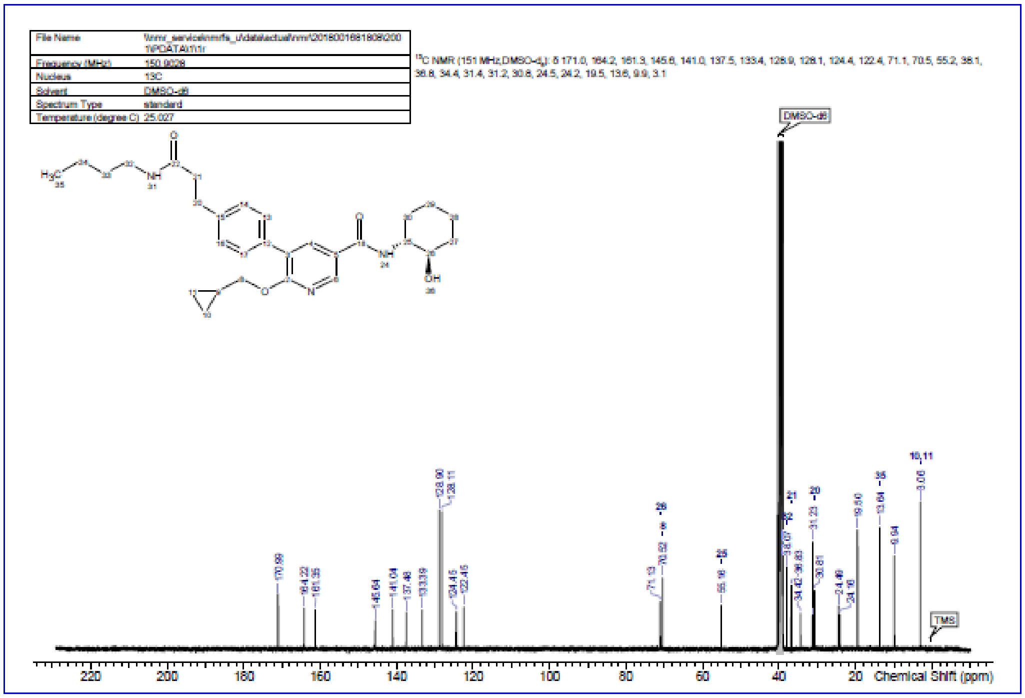

**Synthesis of 5-(4-azido-3-chlorophenyl)-6-(cyclopropylmethoxy)-*N*-[(1*R*,2*R*)-2-hydroxycyclohexyl]- 3-pyridine-2,4-$^{3}H_2$-carboxamide (2,4-$^{3}H_2$ Cpd K)**

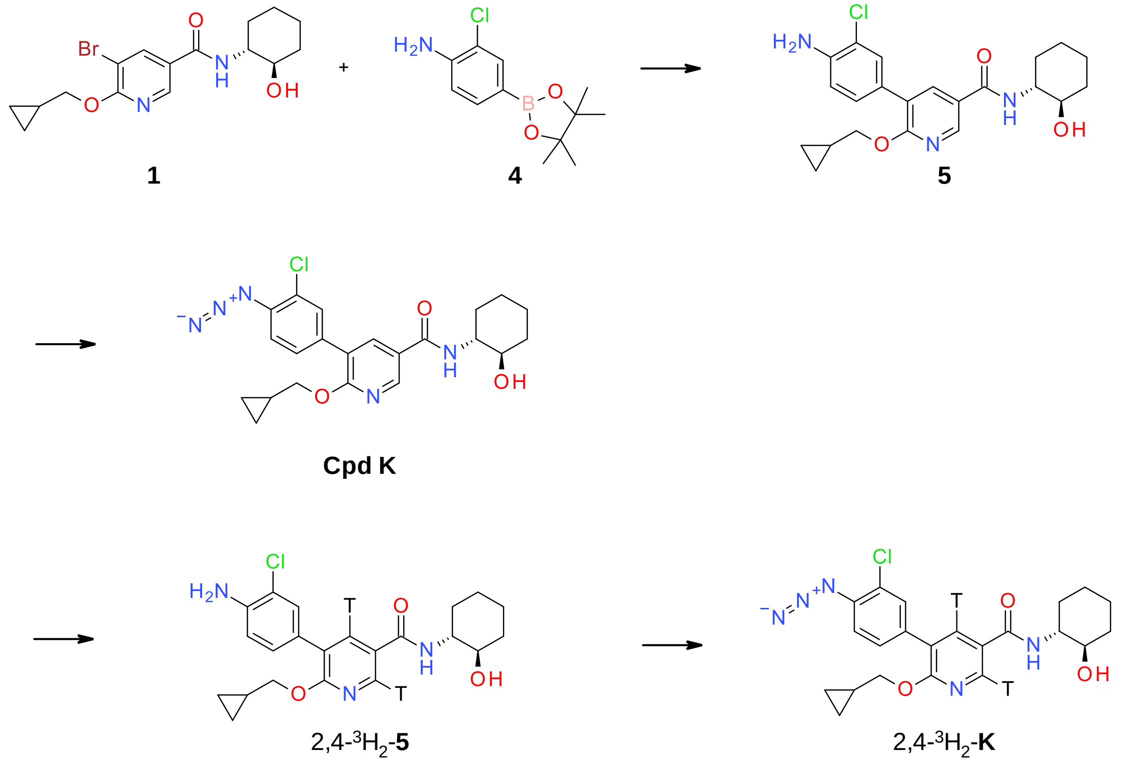


**5-(4-Amino-3-chloro-phenyl)-6-cyclopropylmethoxy-*N*-((1*R*,2*R*)-2-hydroxy-cyclohexyl)-3-pyridinecarboxamide (5)**

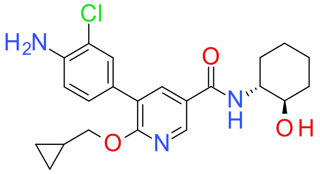

[1,1'-Bis(diphenylphosphino)ferrocene]dichloropalladium(II) x $CH_2Cl_2$ complex (1:1) (276 mg, 0.3 mmol), 2-chloro-4-(4,4,5,5-tetramethyl-1,3,2-dioxaborolan-2-yl)aniline (**4**, 3.3.g, 13 mmol), and sodium carbonate solution (6.5 mL, 2M, 13 mmol) was added to a solution of 5-bromo-6-(cyclopropylmethoxy)-*N*-[(1*R*,2*R*)-2-hydroxycyclohexyl]-3-pyridinecarboxamide (**1**, 2.4 g, 6 mmol) in toluene (50 mL)/DMF (5 mL) and stirred for 16 h at 90°C. Water (100 mL) was added and organic material was extracted into ethyl acetate. After drying with sodium sulfate and evaporation of solvent, the brown oily residue (6.1 g) was purified by flash chromatography (n-heptane, ethyl acetate (1:1)), to afford the title compound as a beige solid (2.5 g, 92%). $^{1}H$ NMR (600 MHz, DMSO-d6) $\delta$ ppm 8.51 (d, *J* = 2.4 Hz, 1H), 8.14 (d, *J* = 8.3 Hz, 1H), 8.13 (d, *J* = 2.4

Hz, 1H), 7.58 (d, $J$ = 2.1 Hz, 1H), 7.38 (dd, $J$ = 8.5, 2.1 Hz, 1H), 6.87 (d, $J$ = 8.5 Hz, 1H), 5.55 (m, 2H), 4.51 - 4.76 (m, 1H), 4.22 (d, $J$ = 7.1 Hz, 2H), 3.56 - 3.66 (m, 1H), 3.37 - 3.46 (m, 1H), 1.88 - 1.94 (m, 1H), 1.80 - 1.86 (m, 1H), 1.59 - 1.68 (m, 2H), 1.18 - 1.29 (m, 5H), 0.52 (m, 2H), 0.34 - 0.36 (m, 2H). $^{13}$C NMR (151 MHz,DMSO-d6): δ 164.3, 161.1, 144.9, 144.3, 136.3, 129.4, 128.5, 124.4, 123.9, 121.6, 116.7, 114.9, 71.1, 70.4, 55.2, 34.4, 31.4, 24.5, 24.2, 9.9, 3.0. HRMS calculated for $C_{22}H_{26}ClN_3O_3$ $[M+H]^+$ 416.1741, found 416.1735.

**$^1$H NMR of compound 5**

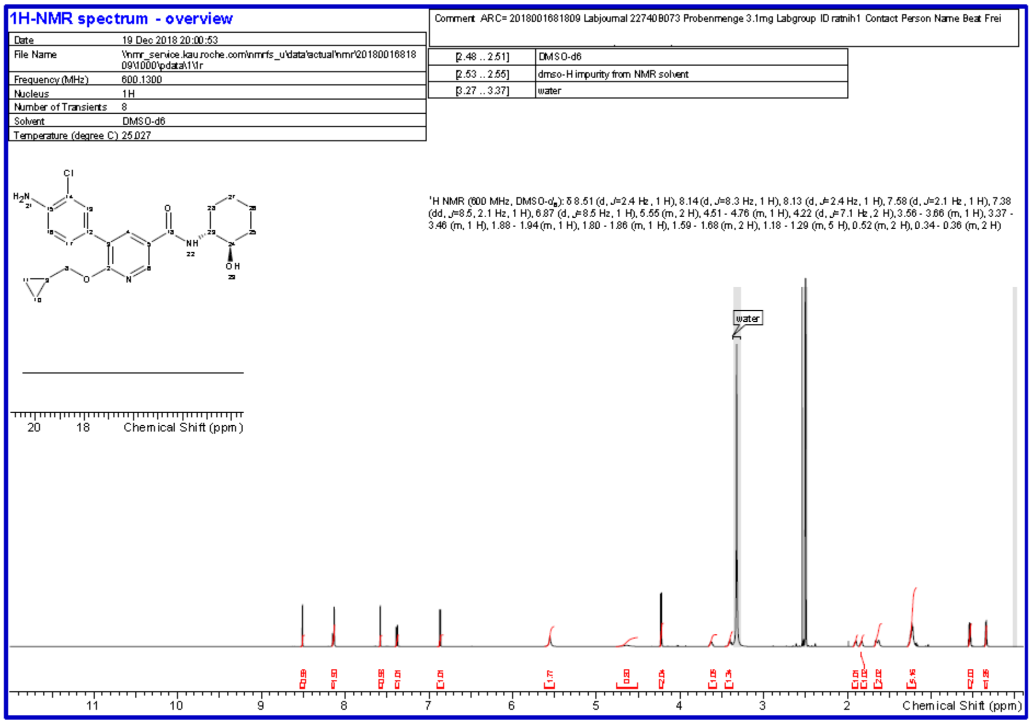


**$^{13}$C NMR of compound 5**

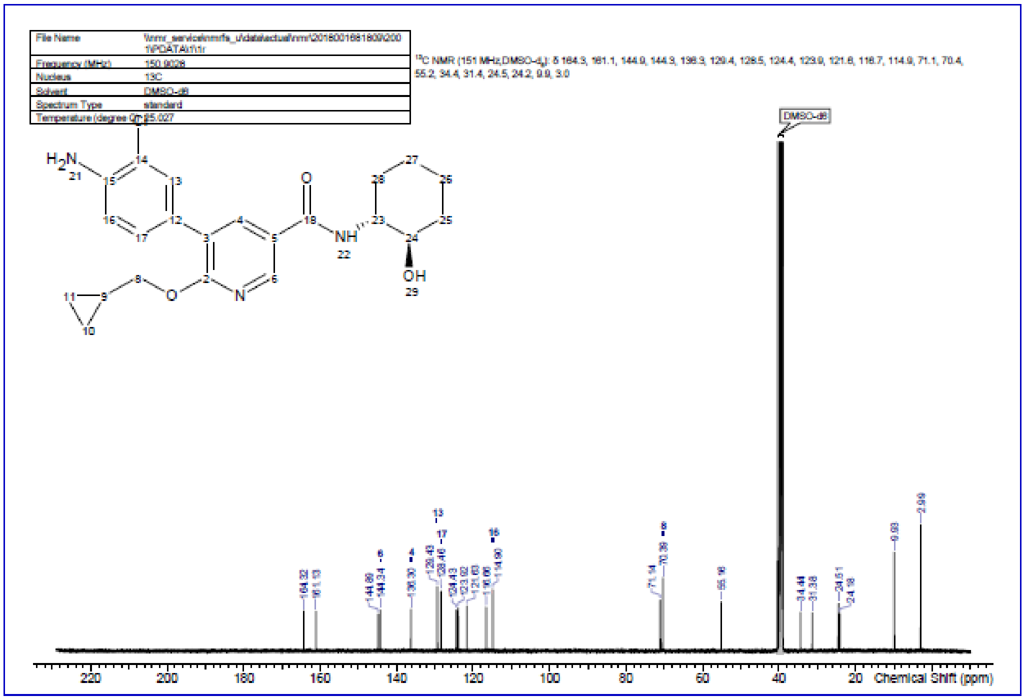


**1,1,1-Trifluoro-methanesulfonyl azide**

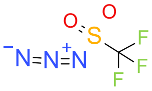


Synthesis and characterization as described in the literature.[4]

**Warning: Unsolvated triflyl azide is a detonation hazard and though we prepared it in a $CH_2Cl_2$ water mixture, supposedly avoiding the formation of explosive azido-chloromethane and diazidomethane, we discourage the use of the procedure for personnel without experience with explosive materials, without extensive safety considerations, as well as the upscaling of the procedure.** And frankly the yield in the step below is not conducive to a repeat.

**5-(4-Azido-3-chlorophenyl)-6-(cyclopropylmethoxy)-*N*-[(1*R*,2*R*)-2-hydroxycyclohexyl]-3-pyridinecarboxamide (Cpd K)**

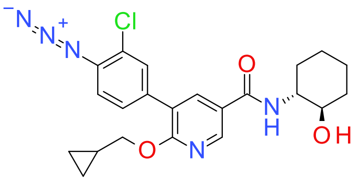

Triethylamine (1 mL, 7 mmol) and copper sulfate (4 mg, 0.02 mmol) was added to a suspension of 5-(4-amino-3-chloro-phenyl)-6-cyclopropylmethoxy-*N*-((1*R*,2*R*)-2-hydroxy-cyclohexyl)-3-pyridinecarboxamide (1.0 g, 2 mmol) in $CH_2Cl_2$ (5 mL). To enable dissolution methanol (1 mL) was added. To this mixture was added dropwise over 30 min a cloudy solution of 1,1,1-trifluoro-methanesulfonyl azide in $CH_2Cl_2$ (10 mL, ~ 7.2 mmol). TLC after stirring for 3 h at room temperature revealed poor conversion. Additional copper sulfate (4 mg, 0.02 mmol) and 1,1,1-trifluoro-methanesulfonyl azide in $CH_2Cl_2$ (10 mL, ~ 2.4 mmol) was added and stirring at room temperature commenced for 20 h. A total of 90 mL sodiumbicarbonate solution (1 N) was added dropwise with stirring and the mixture was extracted with $CH_2Cl_2$.The organic phases were combined washed with sodiumbicarbonate solution and brine and dried with $MgSO_4$. Solvent was removed **(not to dryness and with a protective shield)** till ~20 mL remained and purified by flash chromatography (n-heptane, ethyl acetate gradient, twice), to afford the title compound as a brownish solid (96 mg, 9%). $^1$H NMR (600 MHz, DMSO-d6): δ 8.61 (d, *J* = 2.4 Hz, 1H), 8.22 (d, *J* = 2.4 Hz, 1H), 8.19 (d, *J* = 8.3 Hz, 1H), 7.84 (d, *J* = 2.0 Hz, 1H), 7.74 (dd, *J* = 8.5, 2.0 Hz, 1H), 7.54 (d, *J* = 8.5 Hz, 1H), 4.43 - 4.81 (m, 1H), 4.24 - 4.29 (m, 2H), 3.57 - 3.69 (m, 1H), 3.38 - 3.46 (m, 2H), 1.88 - 1.94 (m, 1H), 1.81 - 1.87 (m, 1H), 1.60 - 1.68 (m, 2H), 1.18 - 1.29 (m, 4H), 0.52 - 0.56 (m, 2H), 0.34 - 0.37 (m, 2H). $^{13}$C NMR (DMSO-d6): δ 164.1, 161.2, 146.5, 137.5, 136.0, 133.6, 130.7, 129.2, 124.5, 123.1, 120.5, 120.3, 71.1, 70.7, 55.2, 34.4, 31.4, 24.5, 24.2, 9.9, 3.0. HRMS calculated for $C_{22}H_{24}ClN_5O_3$ $[M+H]^+$ 442.1646, found 442.1643.

**$^1$H NMR of compound K**

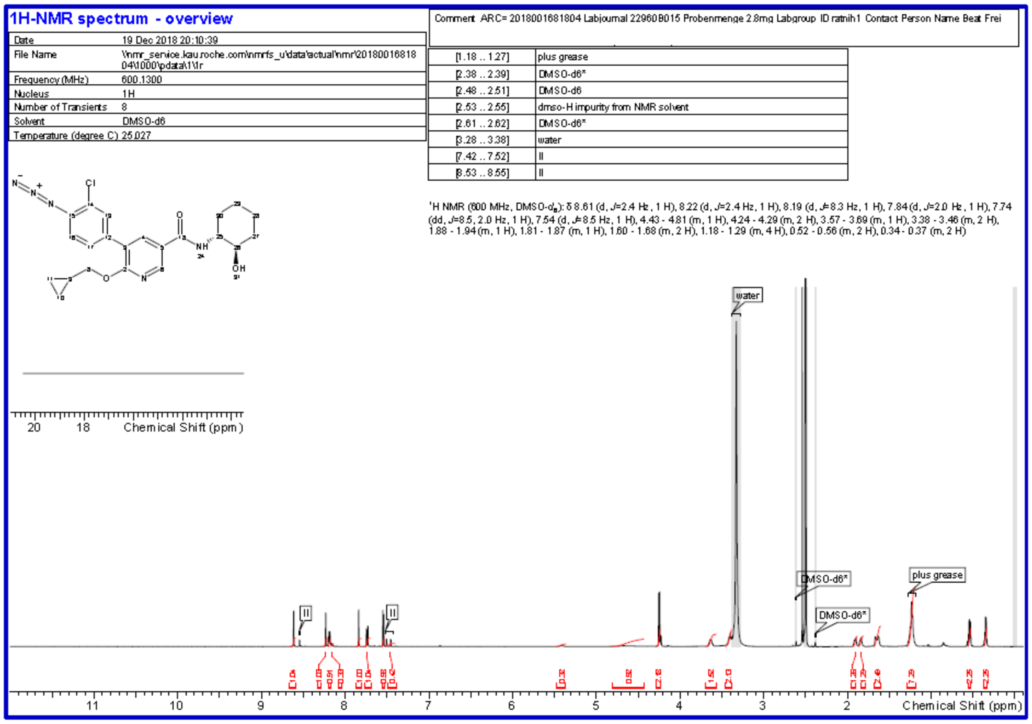


### $^{13}$C NMR of compound K

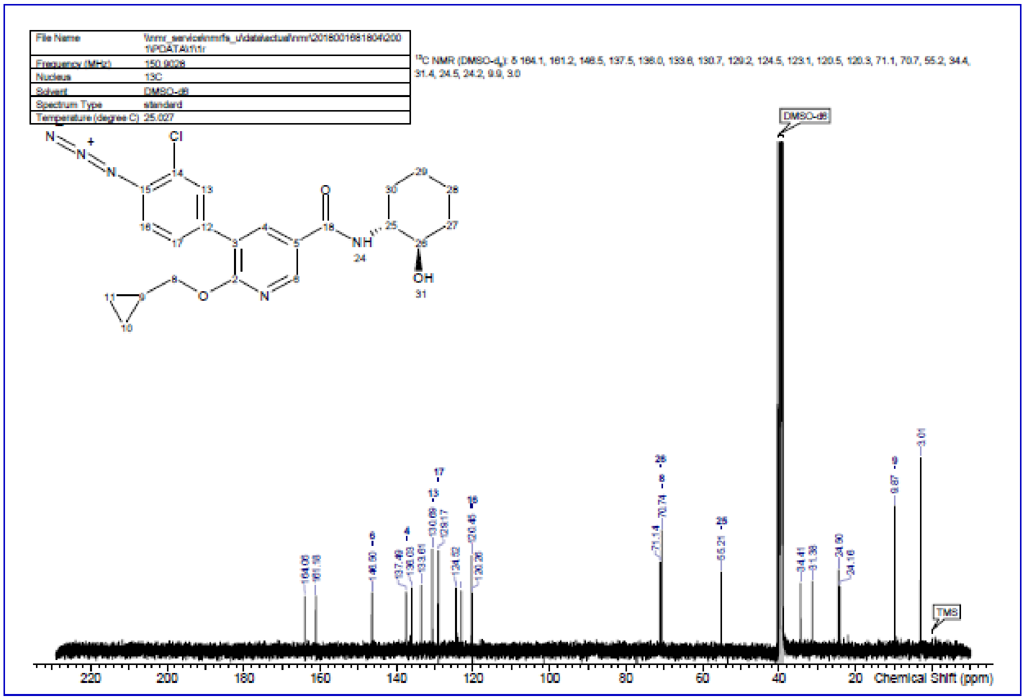


**5-(4-Amino-3-chlorophenyl)-6-(cyclopropylmethoxy)-*N*-[(1*R*,2*R*)-2-hydroxycyclohexyl]-3-pyridine-2,4-ditritio-carboxamide (2,4-$^{3}H_2$-5)**

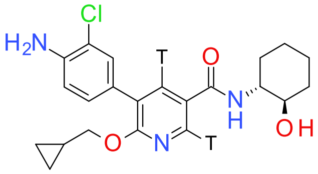


A solution of 5-(4-amino-3-chloro-phenyl)-6-cyclopropylmethoxy-*N*-((1*R*,2*R*)-2-hydroxy-cyclohexyl)-3-pyridinecarboxamide (**5**, 8.5 mg, 20.4 µmol) and tris(dimethylphenylphosphine)(1,5-cyclooctadiene)[1,3-bis(2,4,6-trimethylphenyl)imidazol-2-ylidene]iridium(I) hexafluorophosphate (STREM 77-1830, 2.7 mg, 3 µmol) in $CH_2Cl_2$ (1 mL) was stirred in a $T_2$ atmosphere (1100-621 mbar) for 1.5 h. Volatiles were removed, the residue was dissolved in ethanol and purified by preparative HPLC (LiChroPrepSI60 25-40µm; toluene acetone 3:1) to give the title compound as mixture of tritiated species. HPLC purity 99.5%, 608.11 mCi and according to MS: 5% $t_0$, 3% $t_1$, 20% $t_2$, 59% $t_3$, 14% $t_4$ specific activity 79.4 Ci/mmol.

**5-(4-Azido-3-chlorophenyl)-6-(cyclopropylmethoxy)-*N*-[(1*R*,2*R*)-2-hydroxycyclohexyl]-3-pyridine-2,4-ditritio-carboxamide (2,4-$^3H_2$-K)**

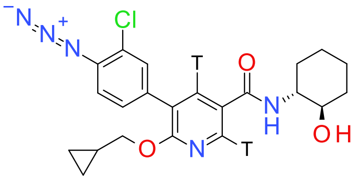


Trifluoroacetic acid (50 µL, 0.65 mmol) was added to a solution of 5-(4-amino-3-chloro-phenyl)-6-cyclopropylmethoxy-*N*-((1*R*,2*R*)-2-hydroxy-cyclohexyl)-2,4-$t_2$-3-pyridinecarboxamide (1.6 mg, 3.8 µmol, 304 mCi, spec. act. 79.4 Ci/ mmol) in THF (0.6 mL) at -10°C. To this mixture sodium nitrite (1 mg, 15 µmol) at -10°C was added and after 30 min of stirring sodium azide (1 mg, 15 µmol) at -5°C was added. Stirring was continued for 1 h at room temperature, THF was removed *in vacuo*, the residue was partitioned between ethyl acetate and sodium bicarbonate, organic phases were combined, dried, concentrated and purified by silica filtration ($CH_2Cl_2$, MeOH 95:5). Preparative HPLC afforded the title compound as mixture of tritiated species (104 mCi, 2.7 T/mol by MS, spec. act. 78 Ci/ mmol).

**Synthesis of 5-(3,4-dichlorophenyl)-*N*-[(1*R*,2*R*)-2-hydroxycyclohexyl]-6-(2,2,2-trifluoroethoxy)-3-pyridinecarboxamide (Cpd G)**

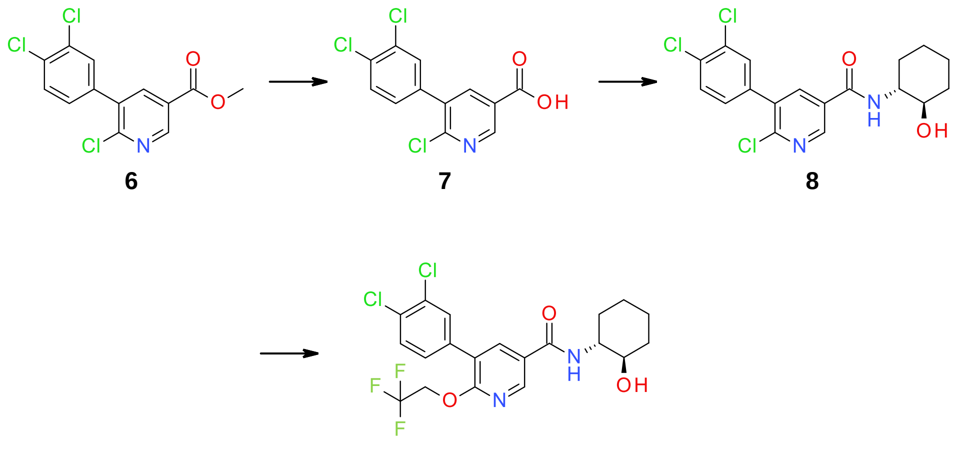

**6** **7** **8**

**Cpd G**

**6-Chloro-5-(3,4-dichlorophenyl)-3-pyridinecarboxylic acid (7)**

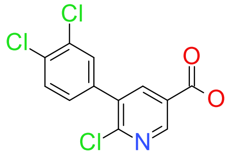

Water (10 mL) and Lithium hydroxide monohydrate (795 mg, 19.0 mmol) was added to a solution of 6-chloro-5-(3,4-dichlorophenyl)-3-pyridinecarboxylic acid methylester (**6**, 3.0 g, 9.5 mmol) in THF (30 mL). The reaction mixture was stirred at reflux for 1h, cooled in an ice-bath and acidified with hydrochloric acid (20 mL, 2N). The mixture was extracted with ethyl acetate, organic layers were combined, dried with $Na_2SO_4$ and concentrated. The title compound precipitated from ethyl acetate/n-heptane to afford a white solid (2.85 g, 99%). $^1$H NMR (DMSO-d6, 600 MHz) δ 13.77 (m, 1H), 8.92 (d, *J* = 2.3 Hz, 1H), 8.26 (d, *J* = 2.3 Hz, 1H), 7.87 (d, *J* = 2.1 Hz, 1H), 7.78 (d, *J* = 8.3 Hz, 1H), 7.55 (dd, *J* = 8.3, 2.1 Hz, 1H). $^{13}$C NMR (151 MHz, DMSO-d6): δ 165.2, 152.0, 149.8, 140.6, 136.8, 133.9, 131.7, 131.3, 131.2, 130.6, 129.8, 126.5. HRMS calculated for $C_{12}H_6Cl_3NO_2$ [M-H]$^-$ 299.9386, found 299.9399.

**$^1$H NMR of compound 7**

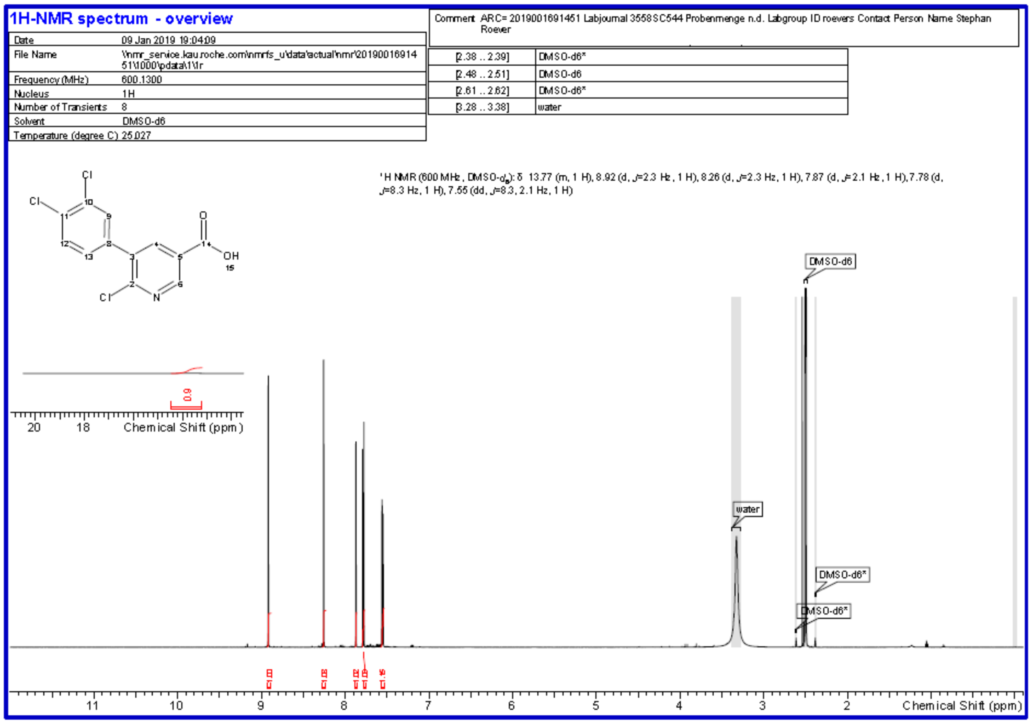


## $^{13}C$ NMR of compound 7

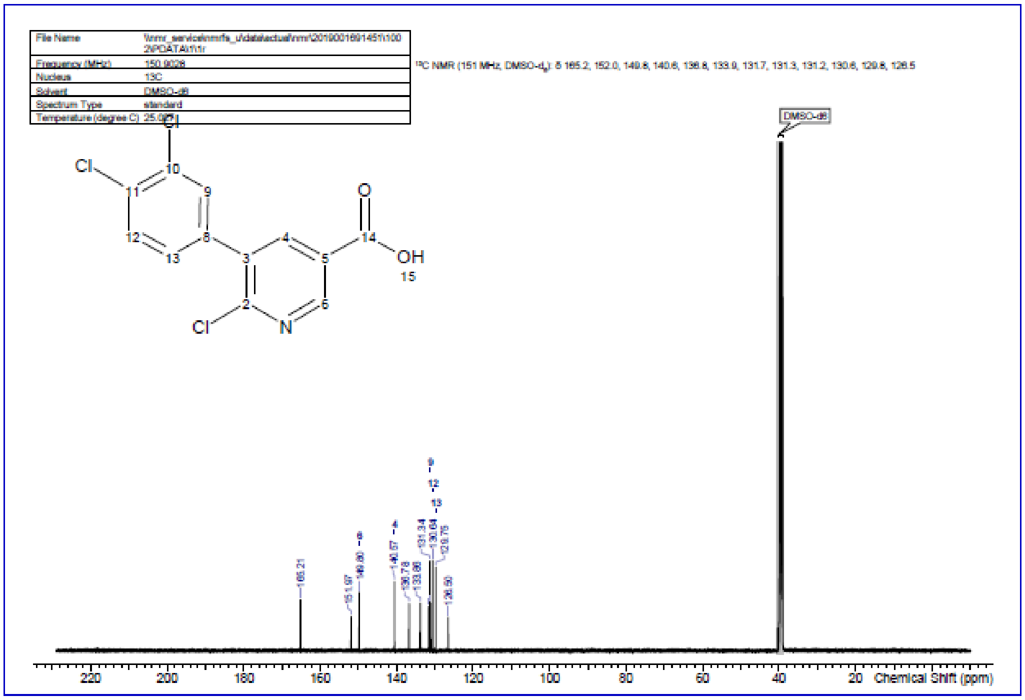


**6-Chloro-5-(3,4-dichlorophenyl)-*N*-((1*R*,2*R*)-2-hydroxycyclohexyl)-3-pyridinecarboxamide (8)**

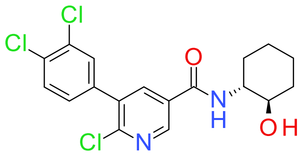


TBTU (2.97 g, 9.25 mmol), *N*,*N*-diisopropylethylamine (7.92 ml, 46.3 mmol) and (1*R*,2*R*)-2-aminocyclohexanol (1.07 g, 9.25 mmol) was added to a solution of 6-chloro-5-(3,4-dichlorophenyl)-3-pyridinecarboxylic acid (**7**, 2.8 g, 9.25 mmol) in DMF (50 mL). The mixture was stirred overnight at room temperature and concentrated (high vacuum) to give a brown residue (8.8 g) which was partitioned between ethyl acetate and sodium hydroxide solution ( 50 mL, 2N). Organic phases were combined, dried with Na2SO4 and concentrated (4.2 g, brown foam). Purification by flash chromatography (n-heptane, ethyl acetate gradient) afforded the title compound as off-white solid (3.1 g, 84%). $^1$H NMR (DMSO-d6, 600 MHz) $\delta$ 8.86 (d, 1H, *J* = 2.3 Hz), 8.4-8.5 (m, 1H), 8.32 (d, 1H, *J* = 2.4 Hz), 7.88 (d, 1H, *J* = 2.1 Hz), 7.82 (d, 1H, *J* = 8.3 Hz), 7.58 (dd, 1H, *J* = 2.1, 8.4 Hz), 4.73 (d, 1H, *J* = 4.9 Hz), 3.6-3.7 (m, 1H), 3.4-3.4 (m, 1H), 1.8-1.9 (m, 2H), 1.6-1.7 (m, 2H), 1.2-1.3 (m, 4H). HRMS calculated for $C_{18}H_{17}Cl_3N_2O_2$ $[M+HCOO]^-$ 443.0332, found 443.0353.

## $^1$H NMR of compound 8

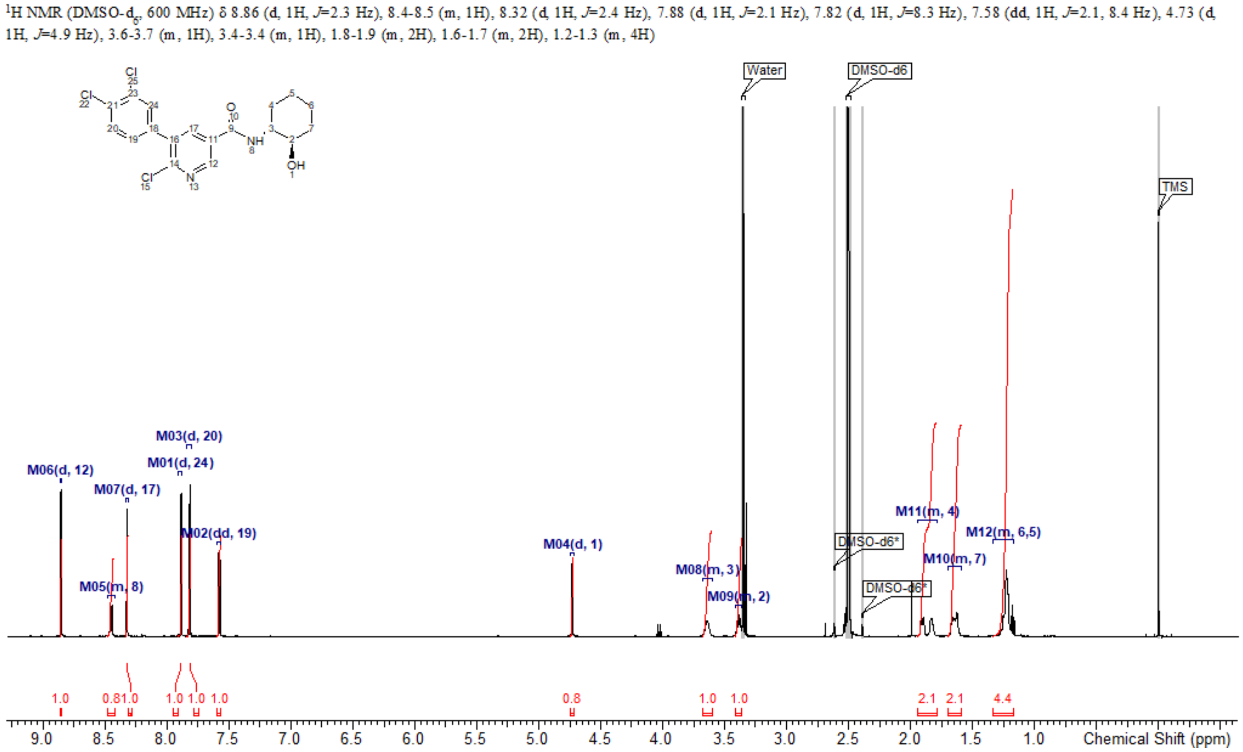

**5-(3,4-Dichlorophenyl)-*N*-[(1*R*,2*R*)-2-hydroxycyclohexyl]-6-(2,2,2-trifluoroethoxy)-3-pyridinecarboxamide (Cpd G)**

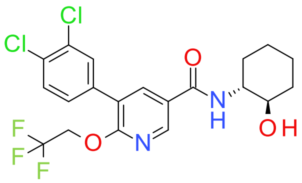


Potassium hydroxide (42.1 mg, 751 µmol) was added to a solution of 6-chloro-5-(3,4-dichlorophenyl)-*N*-((1*R*,2*R*)-2-hydroxycyclohexyl)-3-pyridinecarboxamide (**8**, 100 mg, 250 µmol) in DMSO (1 mL) and stirred for 15 min at room temperature before 2,2,2-trifluoroethanol (23.4 µl, 325 µmol) was added. The reaction mixture was stirred for 1.5 h at room temperature and poured into cold water (30 mL) and hydrochloric acid (1.5 mL, 1 N). The resulting suspension was extracted with ethyl acetate, organic layers were washed with water, combined, dried with $Na_2SO_4$ and concentrated to give a yellow oil (240 mg) that was purified by flash chromatography (n-heptane, ethyl acetate gradient) to give the title compound as white solid (83 mg, 72%). Mp: 175°C. $[\alpha]_D$ = -3.45° ($CHCl_3$, conc: 1.015, 20°C). $^1$H NMR (600 MHz, $CDCl_3$): δ 8.54 (d, *J* = 2.4 Hz, 1H), 8.10 (d, *J* = 2.3 Hz, 1H), 7.67 (d, *J* = 2.1 Hz, 1H), 7.52 (d, *J* = 8.3 Hz, 1H), 7.42 (dd, *J* = 8.3, 2.1 Hz, 1H), 6.13 (br d, *J* = 7.0 Hz, 1H), 4.86 (q, *J* = 8.4 Hz, 2H), 3.90 – 3.83 (m, 1H), 3.49 – 3.43 (m, 1H), 3.28 – 2.69 (m, 1H), 2.17 – 2.09 (m, 2H), 1.82 – 1.75 (m, 2H), 1.45 – 1.24 (m, 4H). $^{13}$C NMR (151 MHz, CDCl3): δ 166.3, 159.9, 145.0, 138.5, 134.4, 132.7, 132.7, 130.9, 130.4, 128.3, 125.7, 122.4, 123.0 (q, *J* = 278.1 Hz), 75.3, 62.7 (q, *J* = 36.3 Hz), 56.4, 34.8, 31.7, 24.6, 24.1. HRMS calculated for $C_{20}H_{19}Cl_2F_3N_2O_3$ $[M+H]^+$ 463.0803, found 463.0808.

**$^1$H NMR of Cpd G**

ARC= 2011000217071 Labjournal A09001689 Labgroup ID zeebd
Contact Person Name Charlotte Haenggi Contact Person Address

$^{1}$H NMR (600 MHz, $CDCl_3$): δ 8.54 (d, $J$ = 2.4 Hz, 1H), 8.10 (d, $J$ = 2.3 Hz, 1H), 7.67 (d, $J$ = 2.1 Hz, 1H), 7.52 (d, $J$ = 8.3 Hz, 1H), 7.42 (dd, $J$ = 8.3, 2.1 Hz, 1H), 6.13 (br d, $J$ = 7.0 Hz, 1H), 4.86 (q, $J$ = 8.4 Hz, 2H), 3.90 – 3.83 (m, 1H), 3.49 – 3.43 (m, 1H), 3.28 – 2.69 (m, 1H), 2.17 – 2.09 (m, 2H), 1.82 – 1.75 (m, 2H), 1.45 – 1.24 (m, 4H)

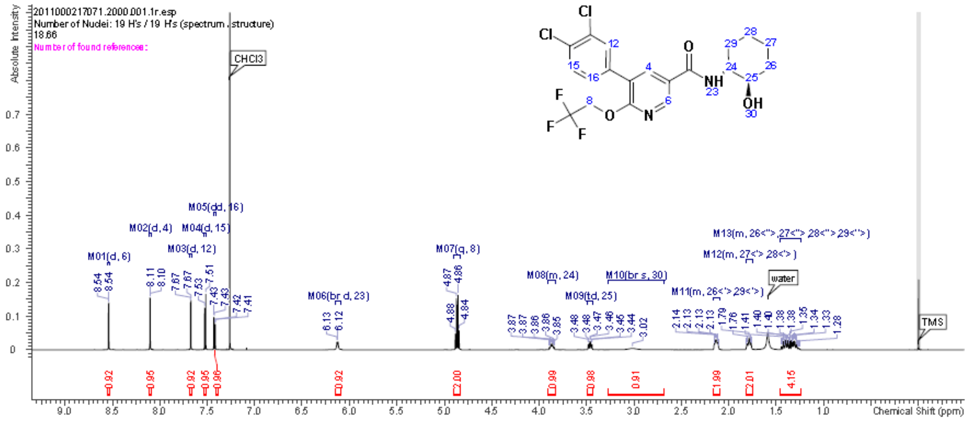


W:\data\archive_back\nmr\2011000217071\2011000217071.2000.001.1r.esp

## $^{13}$C NMR of Cpd G

ARC= 2011000217071 Labjournal A09001689 Labgroup ID zeebd
Contact Person Name Charlotte Haenggi Contact Person Address

$^{13}$C NMR (151 MHz, $CDCl_3$): δ 166.3, 159.9, 145.0, 138.5, 134.4, 132.7, 132.7, 130.9, 130.4, 128.3, 125.7, 122.4, 123.0 (q, $J$=278.1 Hz), 75.3, 62.7 (q, $J$=36.3 Hz), 56.4, 34.8, 31.7, 24.6, 24.1

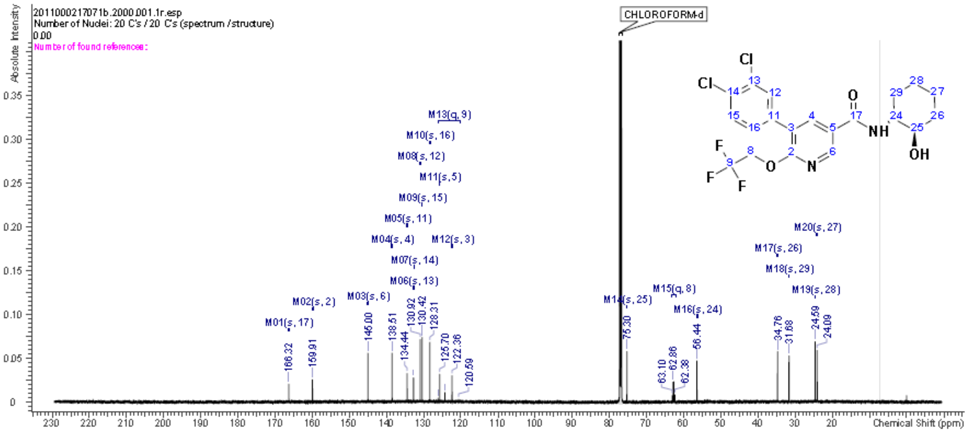


W:\data\archive_back\nmr\2011000217071\2011000217071b.2000.001.1r.esp

**Synthesis of 5-(3,4-dichlorophenyl)-*N*-[(1*S*,2*R*)-2-hydroxycyclohexyl]-6-(2,2,2-trifluoroethoxy)-3-pyridinecarboxamide (Cpd L)**

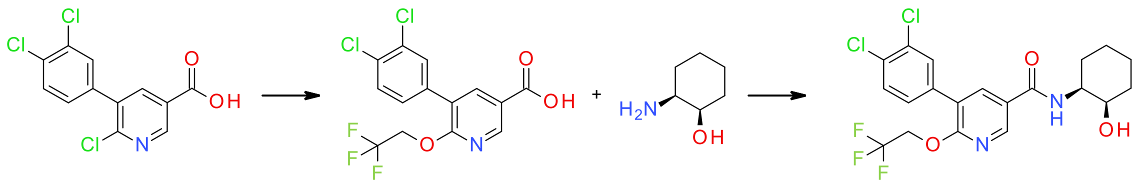

**7** **9** **10** **Cpd L**

**5-(3,4-Dichloro-phenyl)-6-(2,2,2-trifluoro-ethoxy)-3-pyridinecarboxylic acid (9)**

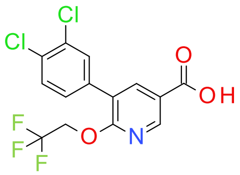

Lithium hydroxide (37.2 g, 1.55 mol, with cooling) and 2,2,2-trifluoroethanol (90 mL, 1.24 mol, dropwise) was added to a solution of 6-chloro-5-(3,4-dichloro-phenyl)-3-pyridinecarboxylic acid (**7**, 187.85 g, 0.62 mol) in DMSO (1250 mL). The reaction mixture was stirred at 75°C for 3.5 h and cooled to room temperature. Water (1300 mL) and hydrochloric acid (202 mL, 25%, dropwise with cooling). The product precipitated, was washed with water, re-dissolved in ethyl acetate (3 L) and THF (500 mL), dried with $Na_2SO_4$ and concentrated *in vacuo* to a volume of ~1300 mL n-Heptane (1500 mL, dropwise) was fully precipitate the title compound as white solid (210 g, 92%). $^{1}$H NMR (DMSO-d6, 600 MHz) δ 13.38 (br d, *J* = 0.7 Hz, 1H), 8.76 (d, *J* = 2.2 Hz, 1H), 8.29 (d, *J* = 2.2 Hz, 1H), 7.91 (d, *J* = 2.1 Hz, 1H), 7.76 (d, *J* = 8.4 Hz, 1H), 7.62 (dd, *J* = 8.4, 2.1 Hz, 1H), 5.11 - 5.15 (m, 2H). $^{13}$C NMR (151 MHz,DMSO-d6): δ 165.6, 160.1, 148.3, 140.3, 135.0, 131.2, 131.1, 130.9, 130.6, 129.2, 123.9 (q, *J* = 277.6 Hz), 122.6, 121.1, 62.3 (q, *J* = 34.9 Hz). HRMS calculated for $C_{14}H_8Cl_2F_3NO_3$ $[M-H]^-$ 363.9755, found 363.9740.

**$^{1}$H NMR of compound 9**

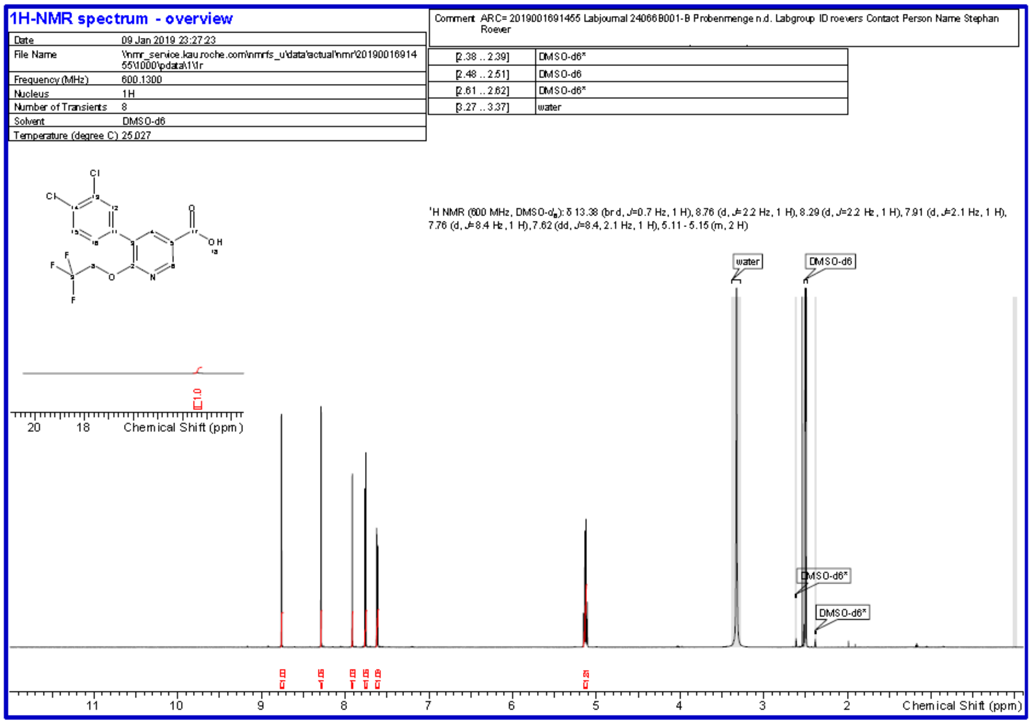


## $^{13}$C NMR of compound 9

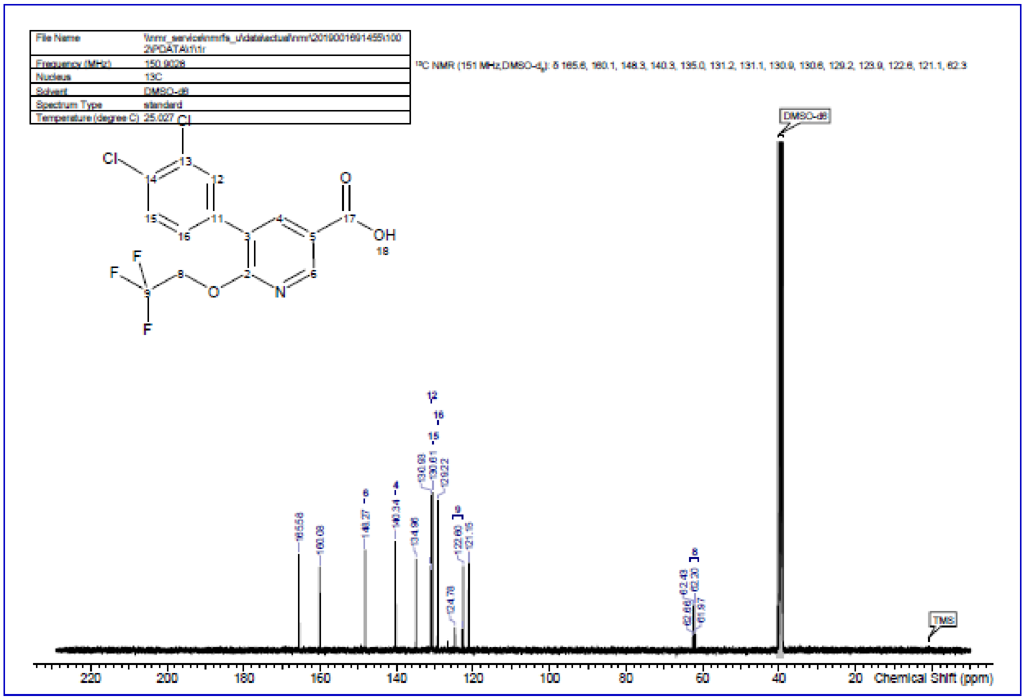


**5-(3,4-Dichlorophenyl)-*N*-[(1*S*,2*R*)-2-hydroxycyclohexyl]-6-(2,2,2-trifluoroethoxy)-3-pyridinecarboxamide (Cpd L)**

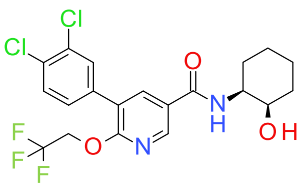

DMF (1.6 mL, 21 mmol) and oxalyl chloride (58.2 mL, 0.677 mol, dropwise, cooling, below 25°C) was added to a solution of 5-(3,4-dichloro-phenyl)-6-(2,2,2-trifluoro-ethoxy)-3-pyridinecarboxylic acid (155 g, 0.42 mol) in THF (1500 mL). After stirring for 1h at room temperature a mixture of (1*R*,2*S*)-2-aminocyclohexanol (89.9 g, 0.59 mol) and concentrated sodium hydroxide solution (196 mL, 32%, 2.12 mol) was added dropwise with cooling (22-29°C).

Stirring continued for 1 h at room temperature and the mixture was poured with stirring into cold water (20 L). Sodium chloride (1 kg) was added and the mixture was extracted with ethyl acetate. Organic layers were combined, dried and concentrated. The residue was purified by silica chromatography (ethyl acetate/heptane (1:1)) to afford the title compound as white solid (183 g, 93%). Mp: 132°C. $[\alpha]_D$ = -18.1° ($CHCl_3$, conc: 1.0, 20°C). $^1H$ NMR (DMSO-d6, 600 MHz) δ 8.68 (d, *J* = 2.3 Hz, 1H), 8.37 (d, *J* = 2.3 Hz, 1H), 8.16 (d, *J* = 7.9 Hz, 1H), 7.94 (d, *J* = 2.2 Hz, 1H), 7.79 (d, *J* = 8.4 Hz, 1H), 7.66 (dd, *J* = 8.4, 2.2 Hz, 1H), 5.11 (q, *J* = 9.0 Hz, 2H), 4.67 (m, 1H), 3.84 - 3.91 (m, 2H), 1.71 - 1.82 (m, 2H), 1.64 - 1.70 (m, 1H), 1.51 - 1.61 (m, 1H), 1.43 - 1.51 (m, 2H), 1.26 - 1.37 (m, 2H). $^{13}C$ NMR (151 MHz,DMSO-d6): δ 163.3, 158.9, 146.6, 138.9, 135.4, 131.1, 130.9, 130.9, 130.5, 129.3, 126.2, 124.0 (q, *J* = 277.9 Hz), 120.1, 66.4, 62.0 (q, *J* = 34.9 Hz), 51.9, 31.8, 26.1, 24.3, 19.2. HRMS calculated for $C_{20}H_{19}Cl_2F_3N_2O_3$ $[M+H]^+$ 463.0803, found 463.0795.

**$^1$H NMR of Cpd L**

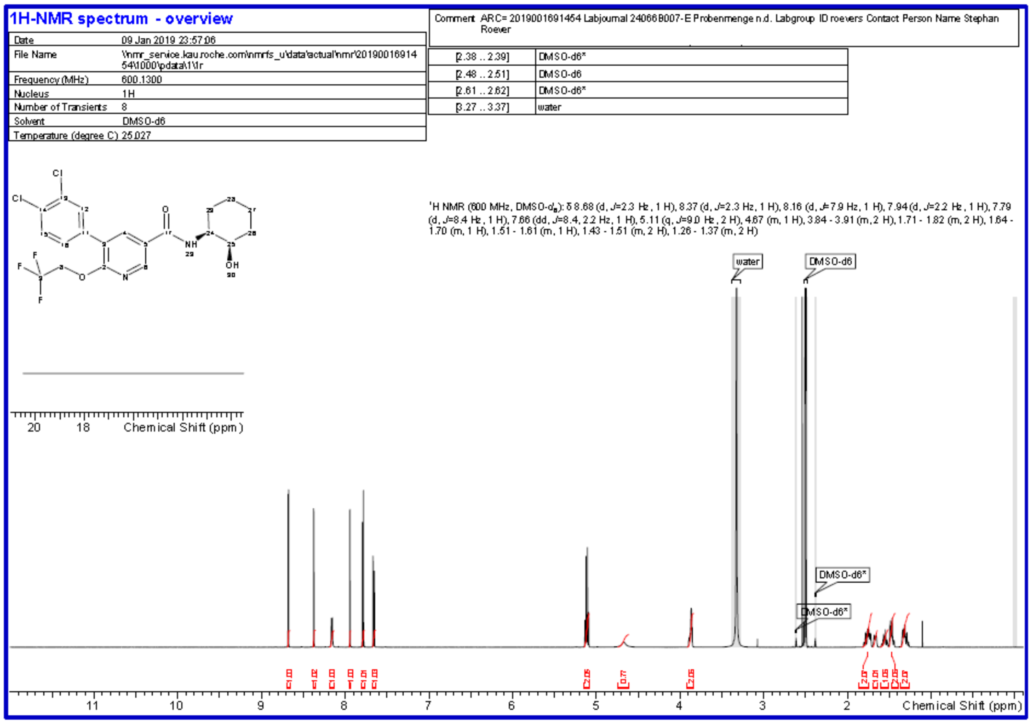


## $^{13}C$ NMR of Cpd L

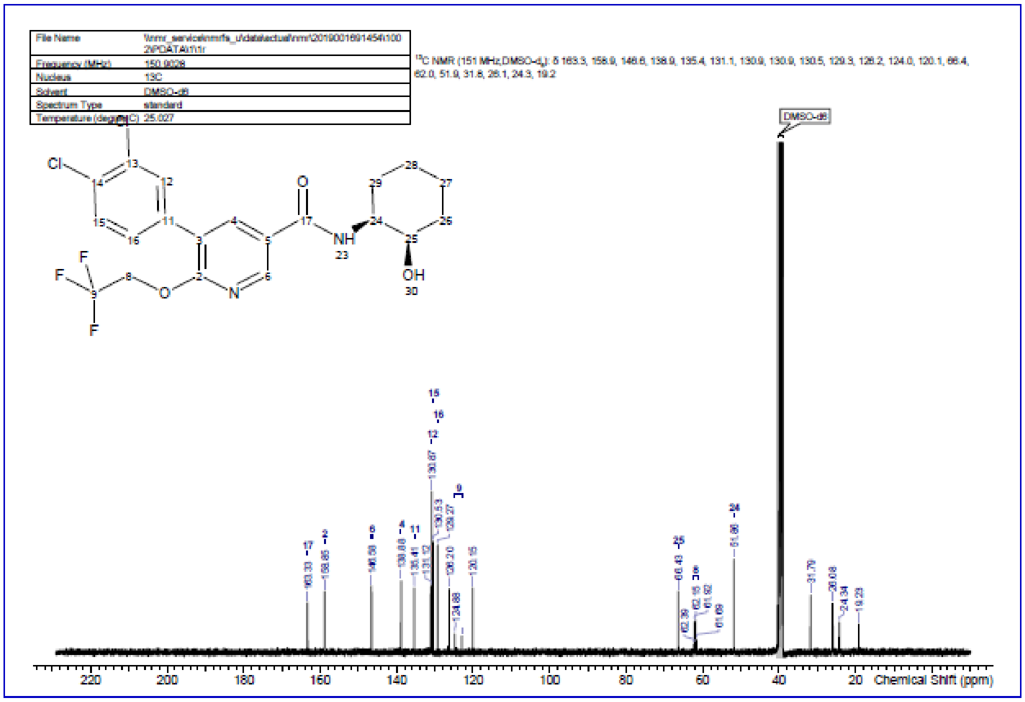

**Synthesis of 4-(4-chlorophenyl)-*N*-(1-methyl-2-oxo-3-pyridyl)-5-(2,2,2-trifluoroethoxy)pyridine-2-carboxamide (Cpd P)**

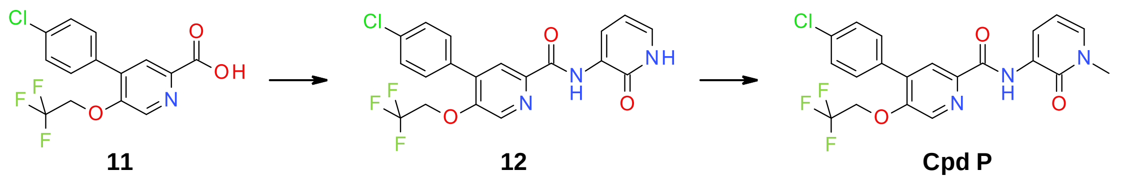

**4-(4-Chloro-phenyl)-5-(2,2,2-trifluoro-ethoxy)-pyridine-2-carboxylic acid (11)**

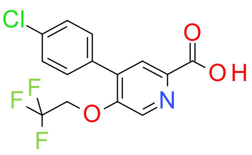

Synthesis and characterization as described in the literature.[1]

**4-(4-Chloro-phenyl)-5-(2,2,2-trifluoro-ethoxy)-pyridine-2-carboxylic acid (2-hydroxy-pyridin-3-yl)-amide (12)**

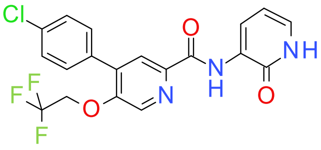

2-(1H-Benzotriazole-1-yl)-1,1,3,3-tetramethyluronium tetrafluoroborate (TBTU, 106 mg, 0.3 mmol), *N*,*N*-diisopropyl ethyl amine (260 µL, 1.5 mmol) and 3-amino-2-hydroxypyridine (36 mg, 0.3 mmol) was added to a solution of 4-(4-chloro-phenyl)-5-(2,2,2-trifluoro-ethoxy)-pyridine-2-carboxylic acid (**11**, 100 mg, 0.3 mmol) in DMF (4 mL) and stirred for 16 h at room temperature. The mixture was concentrated in high vacuum and the residue was partitioned between CH2Cl2/ MeOH (9:1) and water (20 mL) with sodium hydroxide solution (3 mL, 2 N) added. The combined organic layers were combined, dried with $Na_2SO_4$ and concentrated *in vacuo* (135 mg, yellow solid). The solid was stirred for 30 min in ethyl acetate (3 mL), filtered, washed with ethyl acetate and dried to afford the title compound as white solid (111 mg, 87%). %). In DMSO the compound is present as its tautomer. $^{1}$H NMR (DMSO-d6, 600 MHz) δ 12.14 (m, 1H), 10.59 (m, 1H), 8.70 (m, 1H), 8.43 (dd, *J* = 7.3, 1.9 Hz, 1H), 8.11 (m, 1H), 7.67 - 7.70 (m, 2H), 7.58 - 7.61 (m, 2H), 7.16 - 7.19 (m, 1H), 6.31 (dd, *J* = 6.9 Hz, 1H), 5.11 (q, *J* = 8.7 Hz, 2H). $^{13}$C NMR (151 MHz, DMSO-d6): δ 161.4, 157.3, 152.3, 143.6, 136.8, 135.4, 133.9, 132.8, 130.9, 128.6,

128.5, 128.1, 122.9, 123.6(q, $J$ = 277.9 Hz), 122.3, 105.5, 65.6 (q, $J$ = 34.7 Hz). HRMS calculated for $C_{19}H_{13}ClF_3N_3O_3$ $[M+H]^+$ 424.0676, found 424.0682.

### $^1$H NMR of compound 12

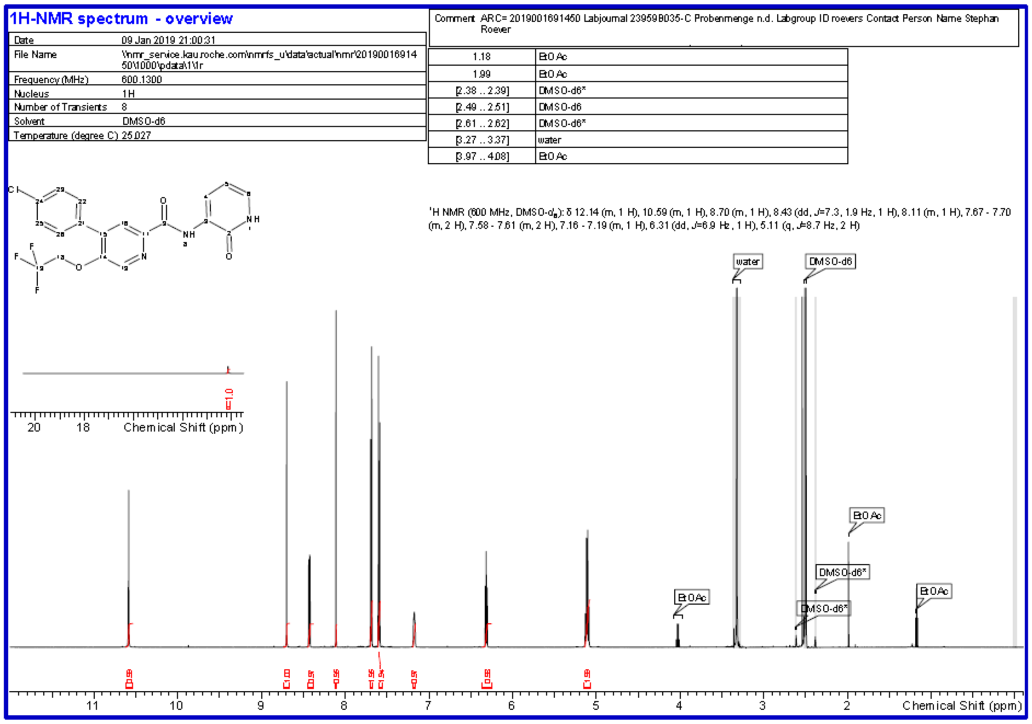


### $^{13}$C NMR of compound 12

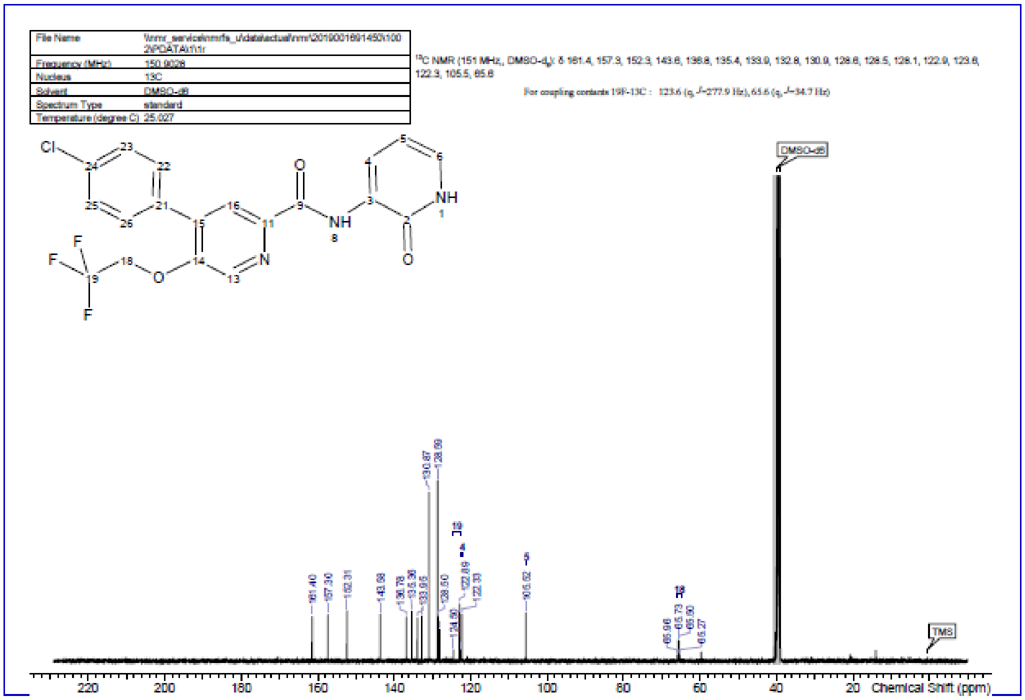


**4-(4-Chlorophenyl)-*N*-(1-methyl-2-oxo-3-pyridyl)-5-(2,2,2-trifluoroethoxy)pyridine-2-carboxamide (Cpd P)**

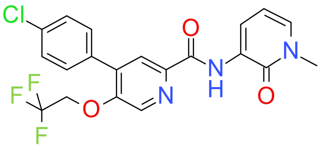

NaH dispersion in oil (60%, 5 mg) was added to a solution of 4-(4-chloro-phenyl)-5-(2,2,2-trifluoro-ethoxy)-pyridine-2-carboxylic acid (2-hydroxy-pyridin-3-yl)-amide (**12**, 50 mg, 0.1 mmol) in THF (2 mL). The mixture was stirred for 1 h at room temperature and then methyl iodide (7.5 µL, 0.1 mmol). Stirring commenced for 20 h at room temperature, water (2 mL) was added and the mixture was extracted with ethyl acetate. Organic layers were combined, dried with $MgSO_4$ and concentrated in vacuo to give a light-yellow solid (52 mg). This material was purified by silica chromatography (ethyl acetate/heptane gradient) to afford the title compound as white solid (37 mg, 71%). $^{1}$H NMR (DMSO-d6, 600 MHz) δ 10.64 (m, 1H), 8.71 (m, 1H), 8.42 (dd, *J* = 7.4, 1.8 Hz, 1H), 8.11 (m, 1H), 7.69 (d, *J* = 8.8 Hz, 2H), 7.59 (d, *J* = 8.8 Hz, 2H), 7.49 (dd, *J* = 6.9, 1.8 Hz, 1H), 6.35 (t, *J* = 7.1 Hz, 1H), 5.12 (q, *J* = 8.7 Hz, 2H), 3.56 (m, 3H). $^{13}$C NMR (151 MHz,DMSO-d6): δ 161.5, 157.1, 152.3, 143.5, 136.8, 135.4, 133.9, 132.8, 132.8, 130.9, 128.6,

127.8, 122.9, 123.6 (q, $J$ = 277.9 Hz), 121.4, 105.4, 65.6 (q, $J$ = 34.8 Hz), 37.2. HRMS calculated for $C_{20}H_{15}ClF_3N_3O_3$ $[M+H]^+$ 438.0832, found 438.0829.

### $^{1}$H NMR of compound P

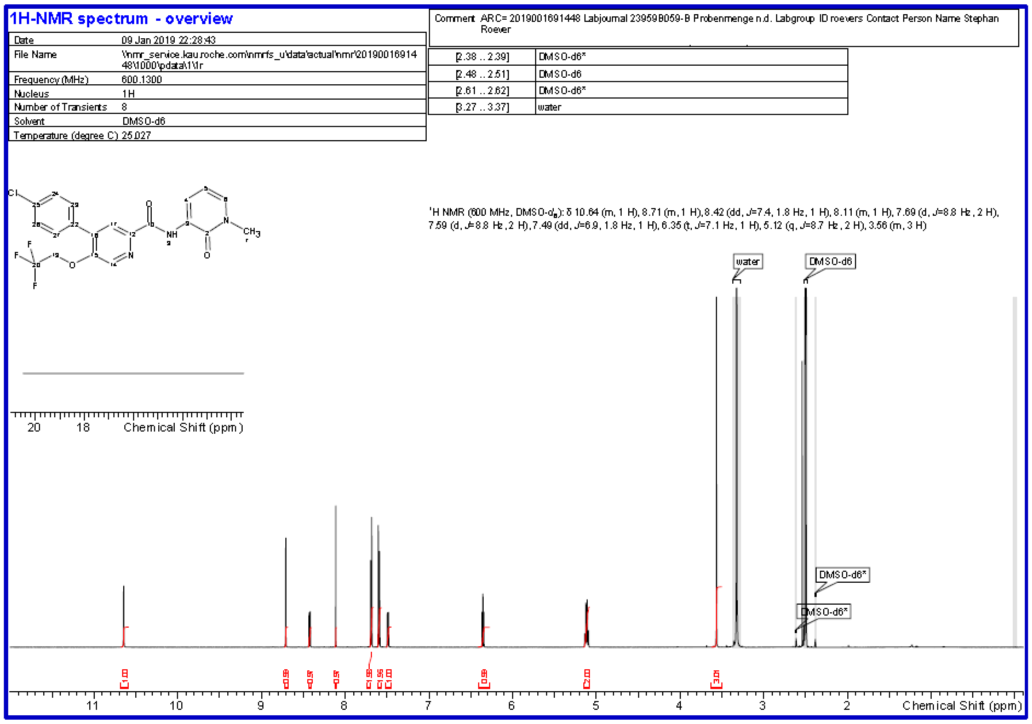


### $^{13}$C NMR of compound P

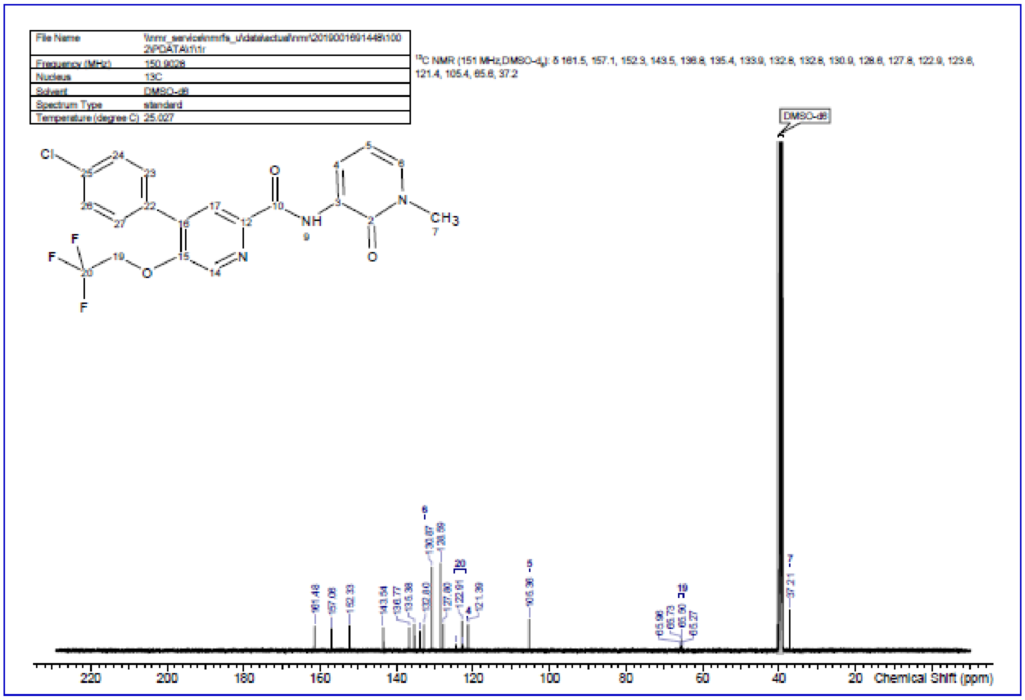

Frequency (MHz) 150.9028
Nucleus 13C
Solvent DMSO-d6
Spectrum Type standard
Temperature (degree C) 25.027
13C NMR (151 MHz,DMSO-d6): δ 161.5, 157.1, 152.3, 143.5, 136.8, 135.4, 133.9, 132.8, 132.6, 130.9, 128.6, 127.8, 122.9, 123.6, 121.4, 105.4, 65.6, 37.2
DMSO-d6
Chemical Shift (ppm)

**Synthesis of *N*-[5-(4-chlorophenyl)-6-(2,2,2-trifluoroethoxy)-3-pyridinyl]-3-pyridinecarboxamide (Cpd M)**

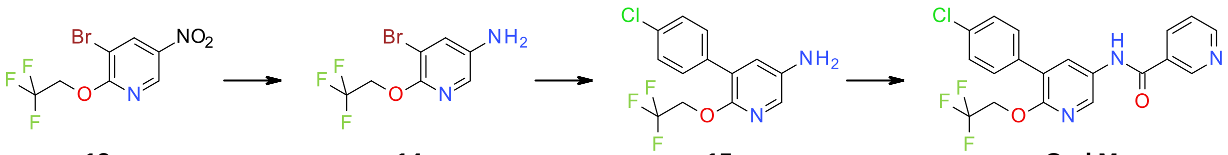

13 14 15 Cpd M

**3-Bromo-5-nitro-2-(2,2,2-trifluoroethoxy)pyridine (13)**

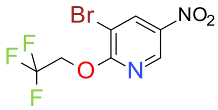

Synthesis and characterization as described in the literature.[2]

**5-Bromo-6-(2,2,2-trifluoroethoxy)-3-pyridin-3-amine (14)**

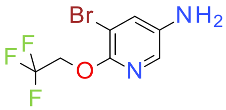

Concentrated hydrochloric acid (4.68 mL, 56. mmol, cooling) and tin (II) chloride (49.6 mg, 262 mmol, portion wise within 15 min at a maximum temperature of 25°C) was added to a solution of 3-bromo-5-nitro-2-(2,2,2-trifluoroethoxy)pyridine (**13**, 11.25 g, 37.4 mmol) in ethanol ( 450 mL). The mixture was stirred for 3 h at room temperature and concentrated *in vacuo*. The residue was taken up in ethyl acetate (300 mL) and ice water (100 mL). Sodium carbonate solution (350 mL, 2 N) was added slowly (gas evolution) and the mixture was filtered through Celite®. Phases were separated and additional material was extracted into ethyl acetate. Organic phases were washed with water and brine, combined, dried with $Na_2SO_4$ and concentrated *in vacuo* to give the title compound as dark oil (10 g) that was used in subsequent steps without further purification. $^1$H NMR (600 MHz, $CDCl_3$): δ 7.55 (d, *J* = 2.6 Hz, 1H), 7.31 (d, *J* = 2.6 Hz, 1H), 4.70 (q, *J* = 8.6 Hz, 2H), 3.47 (br s, 2H). $^{13}$C NMR (151 MHz, $CDCl_3$): δ 151.3, 139.0, 131.0, 130.1, 123.6 (q, *J* = 277.8 Hz), 106.5, 62.9 (q, *J* = 36.0 Hz). HRMS calculated for $C_7H_6BrF_3N_2O$ $[M+H]^+$ 270.9694, found 270.9685.

**$^1$H NMR of compound 14**

User Notes ARC= 2010000176023
Labjournal ELN003048-070-P1
Labgroup ID roevers
Contact Person Name Beat Frei

$^1$H NMR (600 MHz, $CDCl_3$): δ 7.55 (d, $J$ = 2.6 Hz, 1H), 7.31 (d, $J$ = 2.6 Hz, 1H), 4.70 (q, $J$ = 8.6 Hz, 2H), 3.47 (br s, 2H)

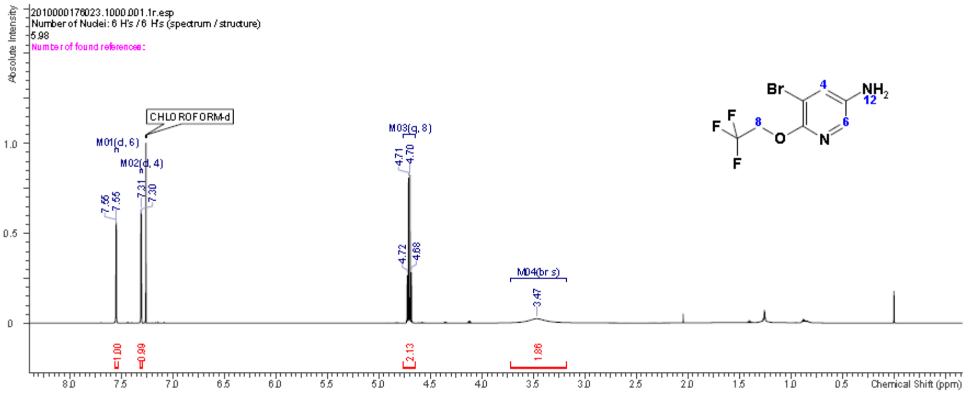


W:\data\archive_back\nmr\2010000176023\2010000176023.1000.001.1r.esp

## $^{13}$C NMR of compound 14

User Notes ARC= 2010000176023
Labjournal ELN003048-070-P1
Labgroup ID roevers
Contact Person Name Beat Frei

$^{13}$C NMR (151 MHz, $CDCl_3$): δ 151.3, 139.0, 131.0, 130.1, 123.6 (q, $J$=277.8 Hz), 106.5, 62.9 (q, $J$=36.0 Hz)

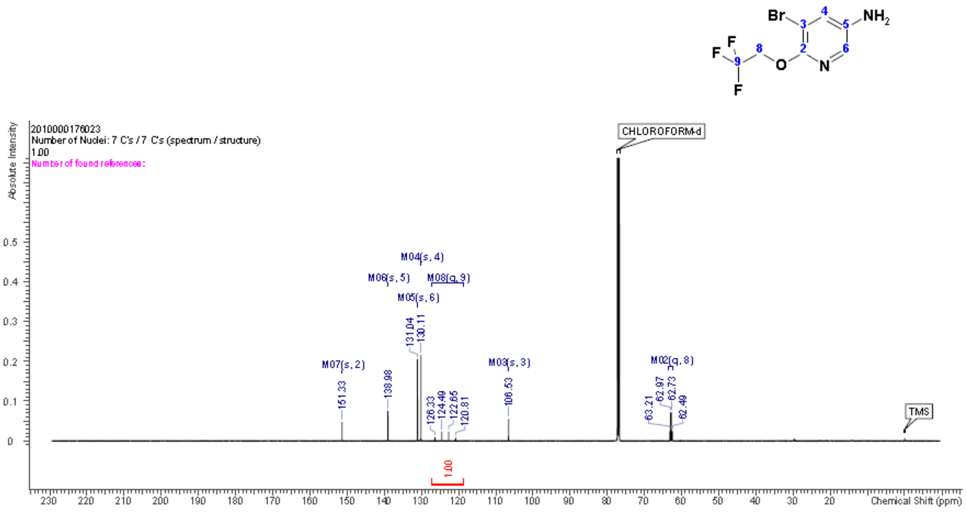


2010000176023

## 5-(4-Chlorophenyl)-6-(2,2,2-trifluoroethoxy)-3-pyridinamine (15)

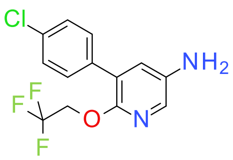


[1,1'-Bis(diphenylphosphino)ferrocene]dichloropalladium (II) x $CH_2Cl_2$(1:1)] (810 mg, 1.11 mmol), 4-chlorophenylboronic acid (5.77 g, 36.9 mmol) and sodium bicarbonate solution (18.4 mL, 36.9 mmol) was added to a solution of 5-bromo-6-(2,2,2-trifluoroethoxy)pyridin-3-amine (**14**, 10 g, 36.9 mmol) in toluene (150 mL). The mixture was stirred for 1.5 h at 90°C, cooled to room temperature and partitioned between water and ethyl acetate. The organic layers were combined, dried with $Na_2SO_4$, and concentrated *in vacuo* to give a brown oil (13 g). The crude material was purified by silica chromatography (ethyl acetate/heptane gradient) to afford, after stirring the product fraction with heptane, filtration and drying the title compound as white solid (10.2 g, 91%). $^1$H NMR (DMSO-d6, 600 MHz) $\delta$ 7.54 (d, *J* = 2.7 Hz, 1H), 7.48 - 7.53 (m, 4H), 7.13 (d, *J* = 2.7 Hz, 1H), 5.07 (m, 2H), 4.86 (q, *J* = 9.2 Hz, 2H). $^{13}$C NMR (151 MHz, DMSO-d6): $\delta$ 149.3, 141.4, 134.9, 132.3, 130.5, 130.2, 128.3, 125.9, 124.3 (q, *J* = 278.1 Hz), 122.0, 61.3 (q, *J* = 34.3 Hz). HRMS calculated for $C_{13}H_{10}ClF_3N_2O$ $[M+H]^+$ 303.0512, found 303.0505.

**$^1$H NMR of compound 15**

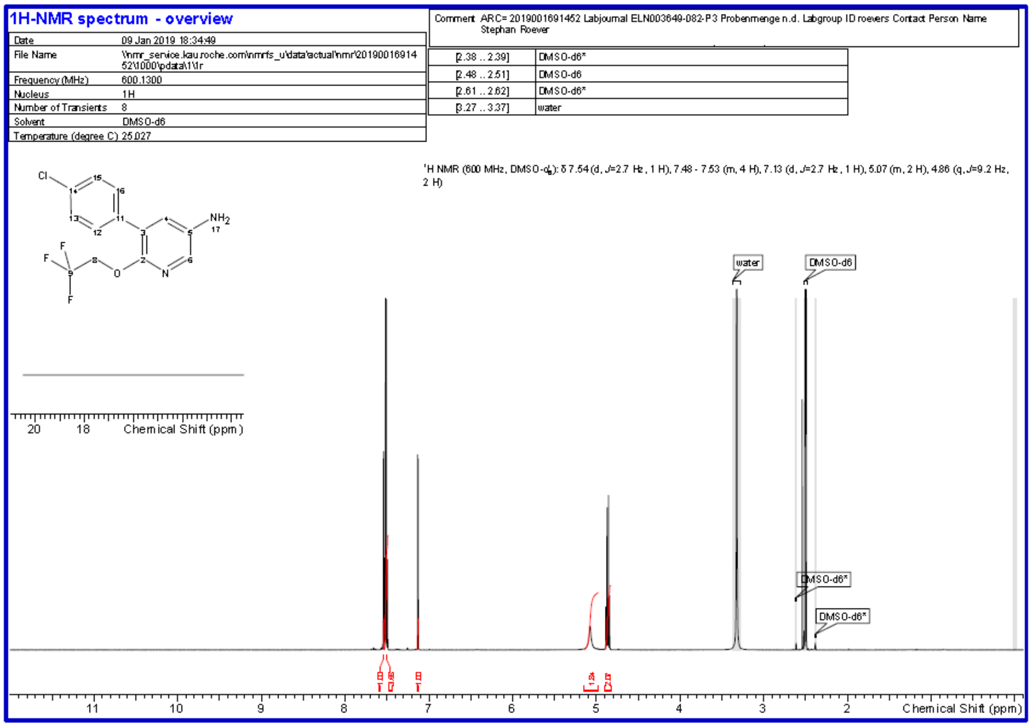


**$^{13}$C NMR of compound 15**

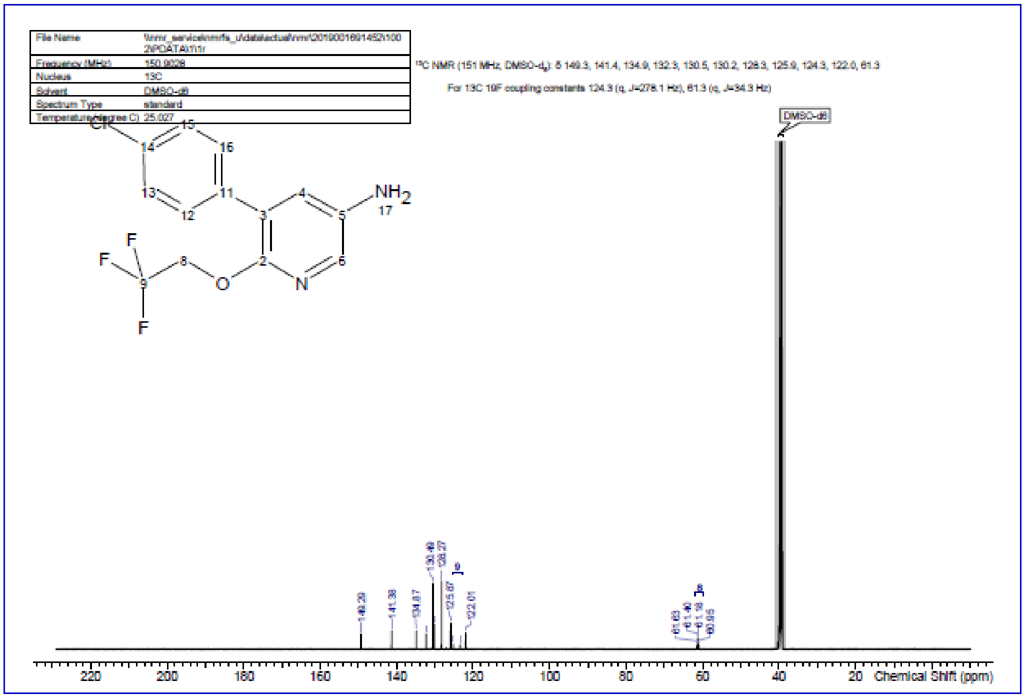


**_N_-[5-(4-Chlorophenyl)-6-(2,2,2-trifluoroethoxy)-3-pyridinyl]-3-pyridinecarboxamide (Cpd M)**

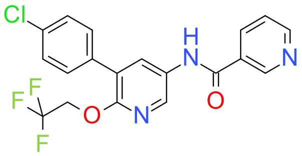

2-(1H-Benzotriazole-1-yl)-1,1,3,3-tetramethyluronium tetrafluoroborate (TBTU, 6.36 g, 19.8 mmol), *N*,*N*-diisopropyl ethyl amine (17 mL, 99.1 mmol) and 5-(4-chlorophenyl)-6-(2,2,2-trifluoroethoxy)pyridin-3-amine (**15**, 6 g, 19.8 mmol) was added to a solution of nicotinic acid (2.44 g, 19.8 mmol) in DMF (200 mL) and stirred for 3 h at room temperature. The mixture was concentrated in high vacuum and the resulting brown oil (19.3 g) was partitioned between ethyl acetate and sodium hydroxide solution (2N) followed by with water. Organic phases were combined, dried with $Na_2SO_4$ and concentrated in vacuo to give a light brown solid (9.3 g) The material was purified by silica chromatography (ethyl acetate/heptane gradient) to afford, after stirring the product fraction with heptane, filtration and drying the title compound as white solid (5.1 g, 63%). Mp.: 171-172°C. $^{1}$H NMR (600 MHz, DMSO-d6): δ 10.65 (s, 1H), 9.14 (dd, *J* = 2.3, 0.7 Hz, 1H), 8.79 (dd, *J* = 4.8, 1.7 Hz, 1H), 8.60 (d, *J* = 2.5 Hz, 1H), 8.34 – 8.31 (m, 1H), 8.26 (d, *J* = 2.5 Hz, 1H), 7.65 – 7.55 (m, 5H), 5.05 (q, *J* = 9.0 Hz, 2H). $^{13}$C NMR (151 MHz,

DMSO-d6): δ 164.1, 153.8, 152.4, 148.7, 137.5, 135.4, 133.9, 132.9, 132.5, 131.6, 130.6, 129.9, 128.5, 123.6, 124.1 (q, $J$ = 277.9 Hz), 122.0, 61.7 (q, $J$ = 34.8 Hz). HRMS calculated for $C_{19}H_{13}ClF_3N_3O_2$ $[M+H]^+$ 408.0727, found 408.0717.

### $^1$H NMR of compound M

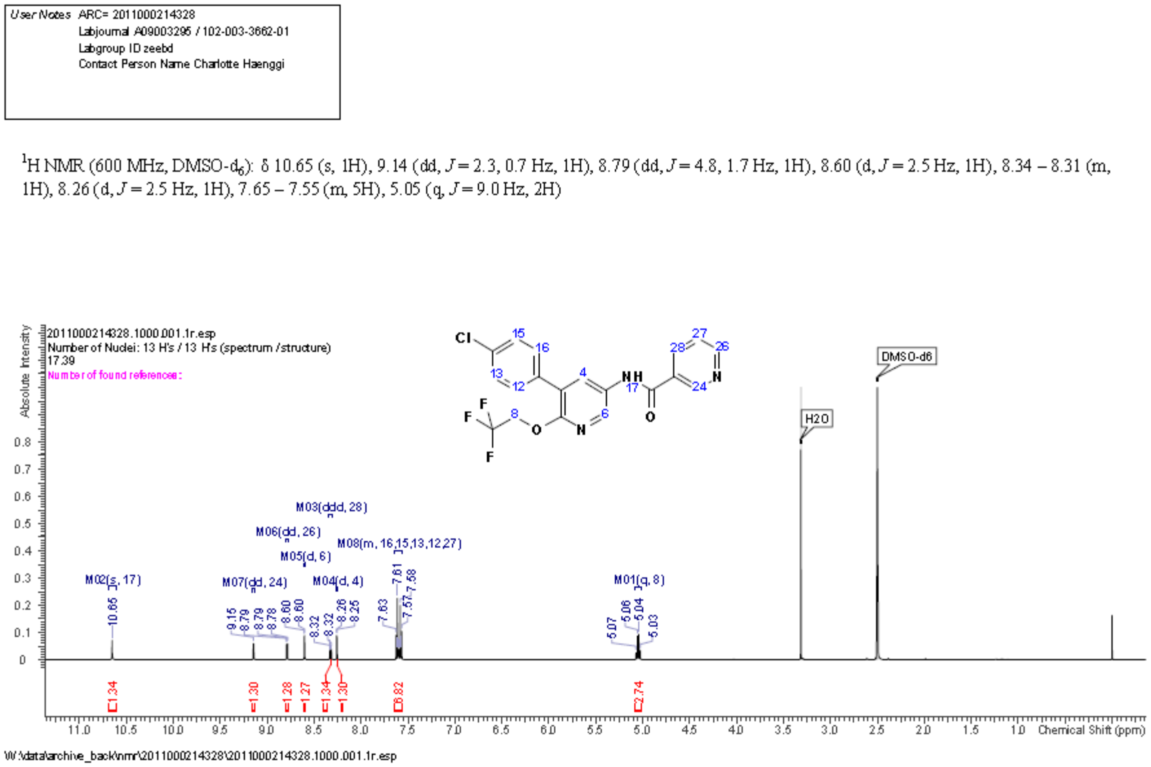


### $^{13}$C NMR of compound M

*User Notes* ARC= 2011000214328
Labjournal A09003295 / 102-003-3662-01
Labgroup ID zeebd
Contact Person Name Charlotte Haenggi

$^{13}$C NMR (151 MHz, DMSO-$d_6$): δ 164.1, 153.8, 152.4, 148.7, 137.5, 135.4, 133.9, 132.9, 132.5, 131.6, 130.6, 129.9, 128.5, 123.6, 124.1 (q, $J$=277.9 Hz), 122.0, 61.7 (q, $J$=34.8 Hz)

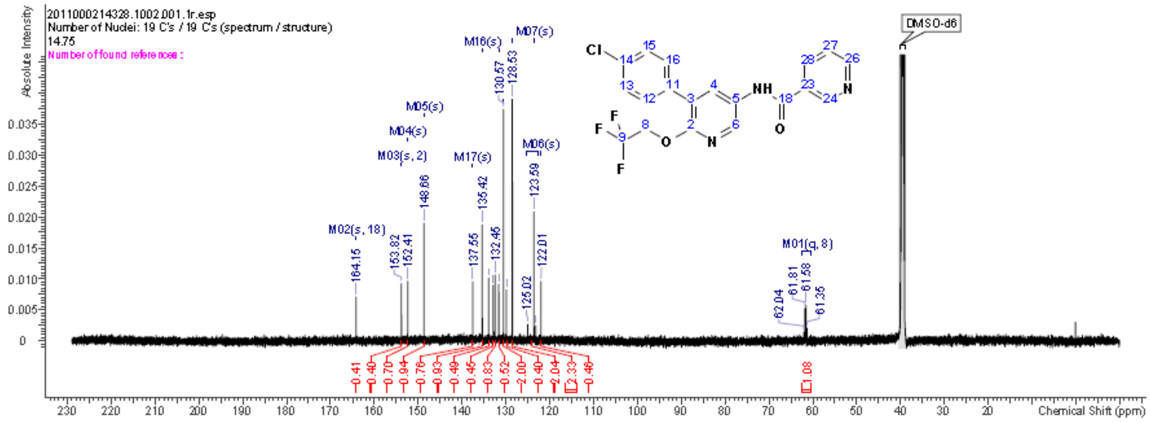


W:\data\archive_back\nmr\2011000214328\2011000214328.1002.001.1r.esp

**Synthesis of 4-(4-chlorophenyl)-*N*-[(3*R*)-2-oxotetrahydropyran-3-yl]-5-(2,2,2-trifluoroethoxy)pyridine-2-carboxamide (Cpd Q)**

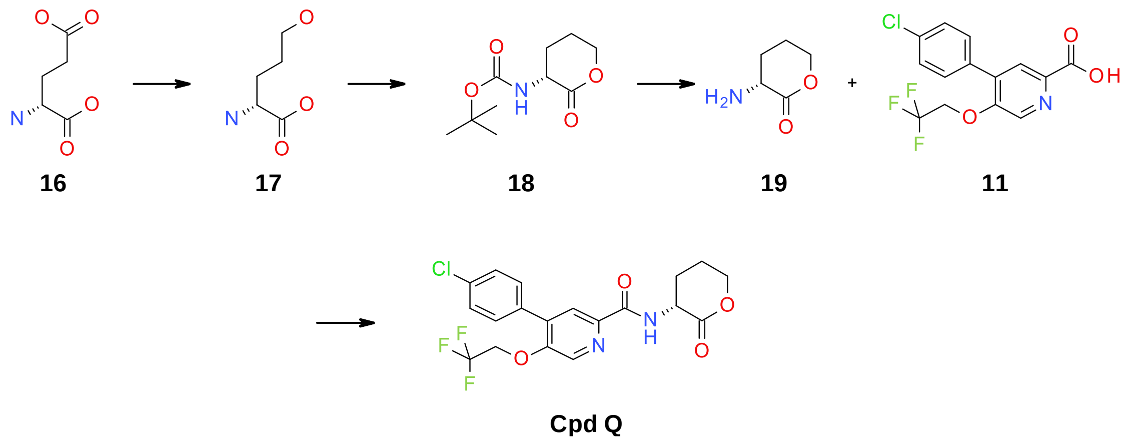

**5-Hydroxy-D-norvaline (17)**

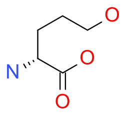

Following the procedure described for the enantiomer,[5] triethylborane solution in THF (100 mL, 1M, 0.1 mol) was added dropwise to a solution of D-glutamic acid (**16**, 12.5 g,85 mmol) in DME (100 mL). The mixture was stirred at reflux temperature over the weekend, cooled to room temperature, filtered, concentrated *in vacuo* and decanted thrice with heptane to give the boron complex as colorless viscous oil (10.8 g, ~50 mmol). This material was dissolved in DME (55 mL) and borane-THF complex (47 mL, 1M, 47 mmol) was added dropwise over a period of 30 min at 3-5°C (strong gas evolution). Cooling was removed and the mixture was strirred for 20 h at room temperature. Hydrochloric acid (40 mL, 1.5M) was added dropwise and the solvent was removed *in vacuo*. Additional hydrochloric acid (50 mL, 1.5 M) was added to the residue and the mixture was stirred at reflux temperature for 1h. Water and hydrochloric acid was removed in vacuo and by adding and removing MeOH by distillation three times. The residue, a light brown oil (7.6 g) was dissolved in sodium hydroxide solution (200 mL, 0.01 N) and purified by ion exchange chromatography (Amberlyte IRA-410, conditioned with 1 N NaOH, eluted with water, 0.1 N AcOH and 0.5 N AcOH). The product fraction was concentrated in vacuo, and the residue was crystallized with MeOH to give the title compound as white solid (2.3 g, 34%). $^1$H NMR (DMSO-d6, 600 MHz) $\delta$ 7.18 (m, 3H), 3.38 (t, *J* = 6.3 Hz, 2H), 3.14 (dd, *J* = 6.9, 5.6 Hz, 1H), 1.71 - 1.79 (m, 1H), 1.59 (m, 1H), 1.43 - 1.54 (m, 2H). $^{13}$C NMR (151 MHz, DMSO-d6): $\delta$ 169.8, 60.6, 54.1, 28.7, 28.0. HRMS calculated for $C_5H_{11}NO_3$ $[M-H]^-$ 132.0661, found 132.0670.

## $^{1}H$ NMR of compound 17

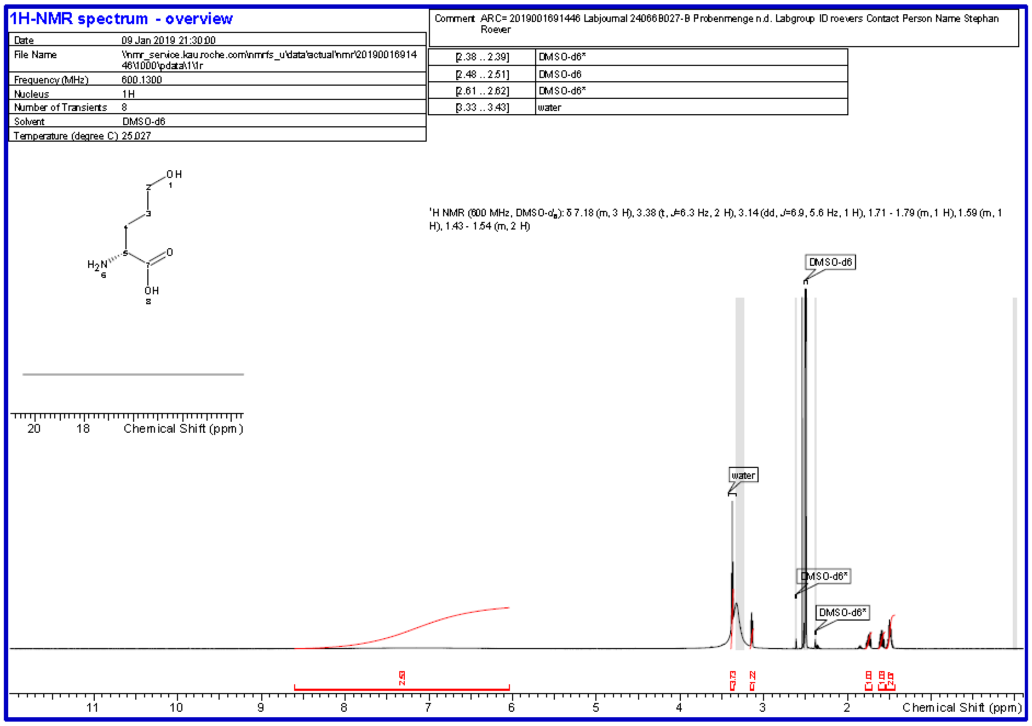


## $^{13}C$ NMR of compound 17

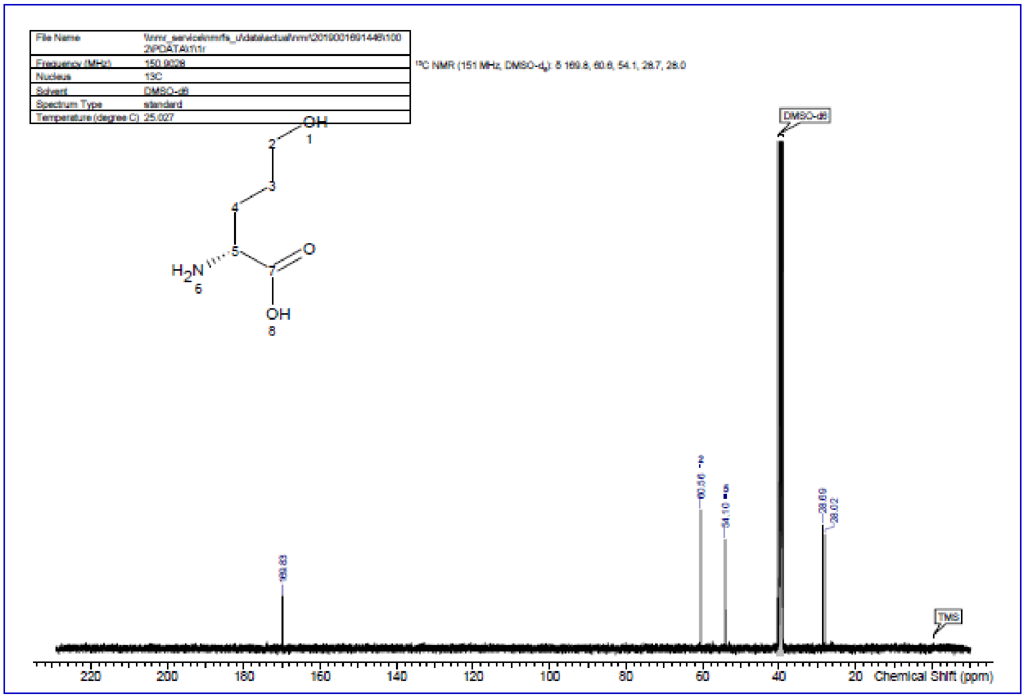

**_N_-[(3_R_)-tetrahydro-2-oxo-2H-pyran-3-yl]-1-carbamic acid 1,1-dimethylethyl ester (18)**

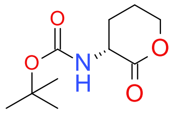

Di-*tert*-butyl-dicarbonate solution (5.7 g, 26 mmol) in THF (20 mL) and 4-dimethylaminopyridine (110 mg, 0.9 mmol) was added to a suspension of 5-hydroxy-D-norvaline (**17**, 2.3 g, 17 mmol) in THF (120 mL). After about 6 h stirring at room temperature an additional amount of di-*tert*-butyl-dicarbonate (1.5 g) was added and stirring commenced overnight. *N*,*N*-diisopropyl ethyl amine (3.0 mL, 17 mmol) and 1-(3-dimethylaminopropyl)-3-ethylcarbodiimide hydrochloride (3.3 g, 17 mmol) was added to the mixture and stirring continued at room temperature for another day. The mixture was then partitioned between ethyl acetate and water, organic phases were washed with brine, combined, dried with $MgSO_4$ and concentrated *in vacuo* to give a reddish-brown oil (5.0 g). This material was purified by flash chromatography (n-heptane, ethyl acetate 2:1) to give the title compound as white solid (1.95 g, 52%). $^1H$ NMR (DMSO-d6, 600 MHz) δ 7.0-7.3 (m, 1H), 4.1-4.4 (m, 3H), 1.7-2.1 (m, 4H), 1.3-1.4 (m, 9H). MS calculated for $C_{10}H_{17}NO_4$ $[M-C_4H_7]^+$ 160, found 160.

**$^1$H NMR of compound 18**

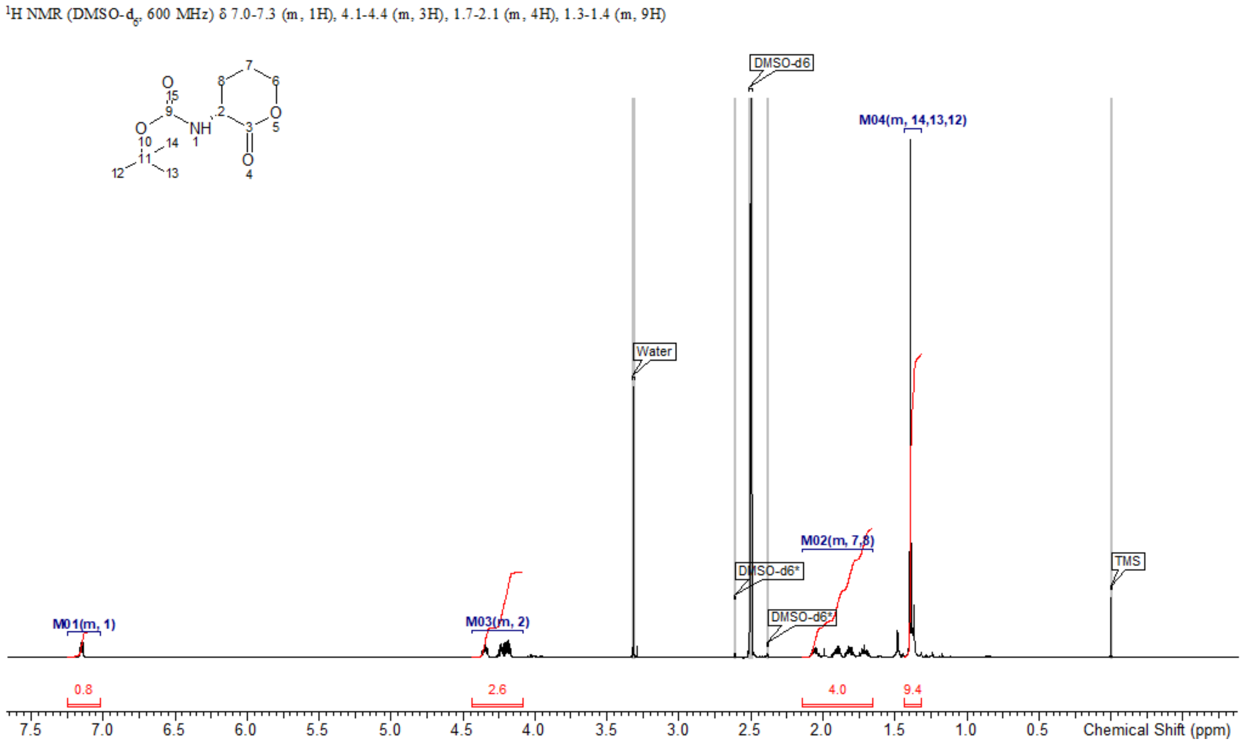

**(3*R*)-3-Amino-tetrahydro-pyran-2-one trifluoro-acetic acid salt (19)**

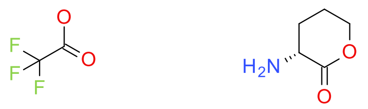

Trifluoroacetic acid (5.0 mL, strong gas evolution) was added to a solution of *N*-[(3*R*)-tetrahydro-2-oxo-2H-pyran-3-yl]-1-carbamic acid 1,1-dimethylethyl ester (**18**, 1.95 g, 9.1 mmol) in $CH_2Cl_2$ (10 mL) and the mixture was stirred for 1 h at room temperature and then concentrated *in vacuo*. The residue was stirred with diethylether (20 mL) to precipitate the trifluoroacetic acid salt of the title compound as white solid (2.1 g, quant.). $[\alpha]_D$ = -29.99° (c 0.8, MeOH, 20°C). $^1H$ NMR (DMSO-d6, 600 MHz) $\delta$ 8.2-8.6 (m, 3H), 4.2-4.5 (m, 3H), 1.7-2.3 (m, 4H). MS calculated for $C_5H_9NO_2$ $[M+H]^+$ 116, found 116.

**$^1H$ NMR of compound 19**

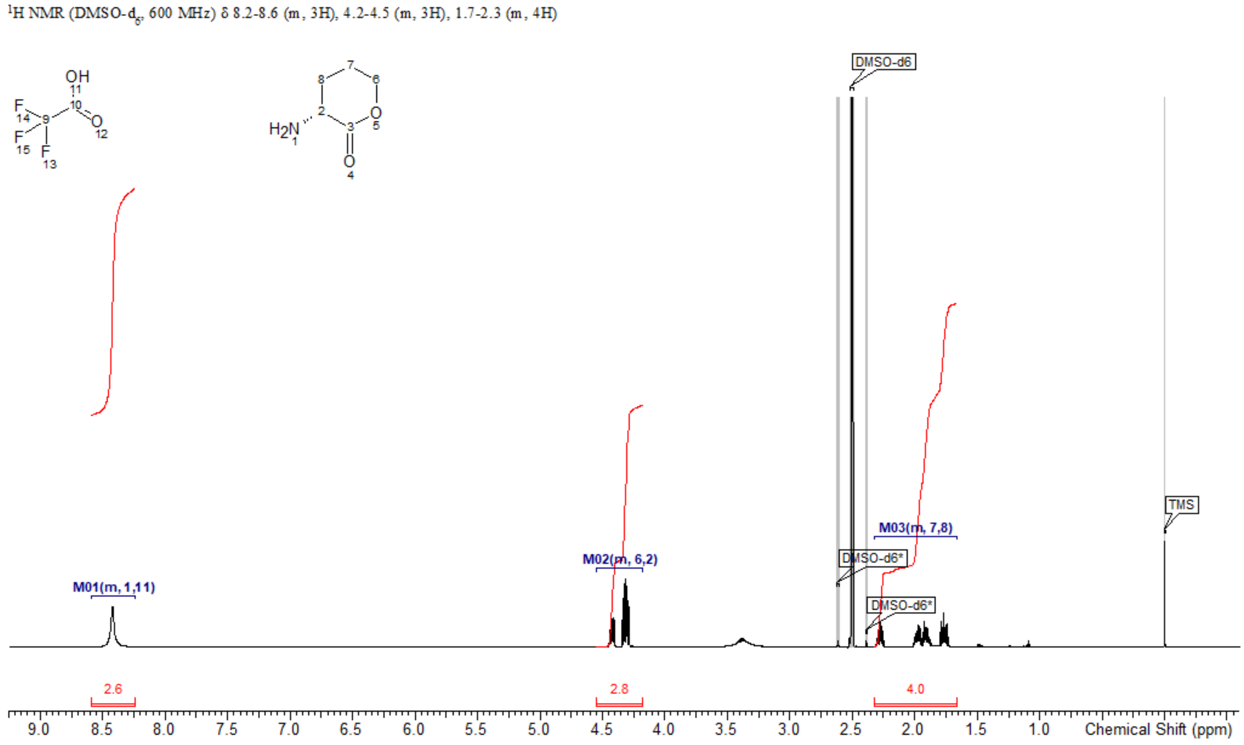


**4-(4-Chlorophenyl)-*N*-[(3*R*)-2-oxotetrahydropyran-3-yl]-5-(2,2,2-trifluoroethoxy)pyridine-2-carboxamide (Cpd Q)**

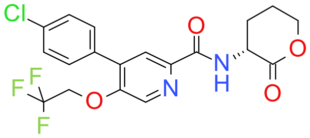


2-(1H-Benzotriazole-1-yl)-1,1,3,3-tetramethyluronium tetrafluoroborate (TBTU, 53 mg, 0.2 mmol), *N*,*N*-diisopropyl ethyl amine (129 µL, 0.8 mmol) and (3*R*)-3-amino-tetrahydro-pyran-2-one trifluoro-acetic acid salt (**19**, 38 mg, 0.2 mmol) was added to a solution of 4-(4-chloro-phenyl)-5-(2,2,2-trifluoro-ethoxy)-pyridine-2-carboxylic acid (50 mg, 0.2 mmol) in DMF (2 mL) and stirred for 6 h at room temperature. The mixture was concentrated in high vacuum, partitioned between ethyl acetate and sodium hydroxide solution (1 N), dried and pre-purified over ChemElut® with ethyl acetate elution to give a light-yellow oil (75 mg). This oil was purified by flash chromatography (n-heptane, ethyl acetate gradient) to give the title compound as white solid (53 mg, 82%). $[\alpha]_D$ = 1.88° (c 0.266, MeOH, 20°C). $^1$H NMR (DMSO-d6, 600 MHz) δ 9.02 (d, *J* = 8.3 Hz, 1H), 8.62 (m, 1H), 8.01 (m, 1H), 7.66 (d, *J* = 8.7 Hz, 2H), 7.58 (d, *J* = 8.7 Hz, 2H), 5.09 (q, *J* = 8.7 Hz, 2H), 4.75 (m, 1H), 4.40 - 4.46 (m, 1H), 4.29 (m, 1H), 2.14 - 2.22 (m, 1H), 1.95 - 2.05 (m, 2H), 1.85 - 1.94 (m, 1H). $^{13}$C NMR (151 MHz,DMSO-d6): δ 171.0, 162.9, 152.0, 144.1, 136.5, 135.1, 133.8, 133.0, 130.8, 128.6, 123.0, 123.6 (q, *J* = 277.6 Hz), 68.2, 65.6 (q, *J* = 34.6 Hz), 48.0, 25.2, 21.5. HRMS calculated for $C_{19}H_{16}ClF_3N_2O_4$ $[M+H]^+$ 429.0829, found 429.0830.

**$^1$H NMR of compound Q**

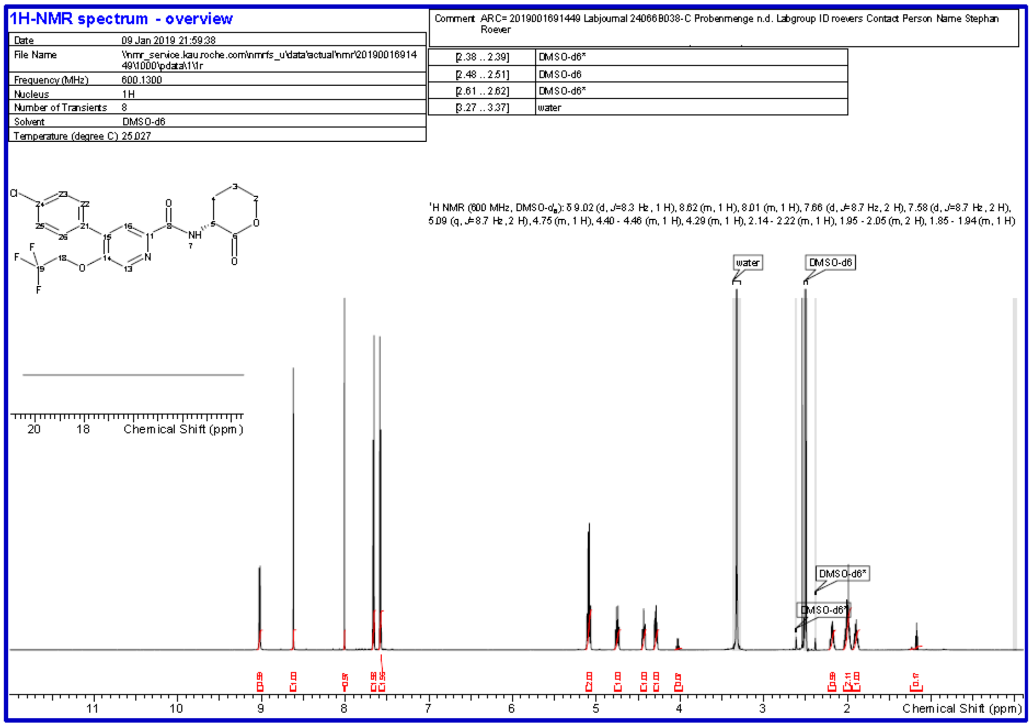


## $^{13}$C NMR of compound Q

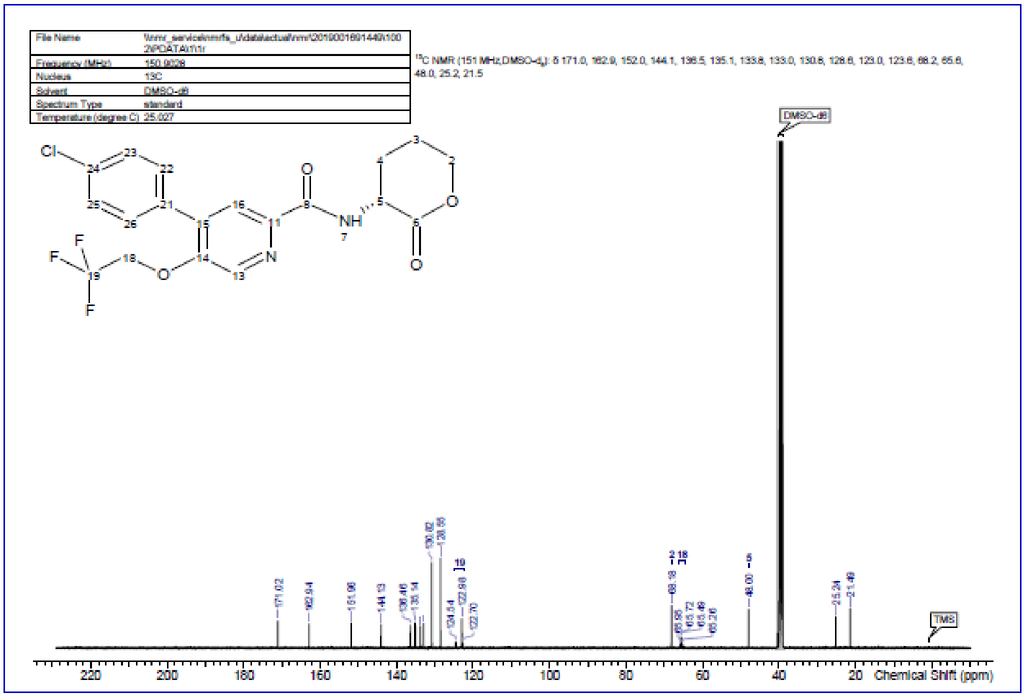

**Synthesis of *N*-[5-(2-methyl-4-pyridyl)-6-(2,2,2-trifluoroethoxy)-3-pyridyl]pyridine-3-carboxamide (Cpd R)**

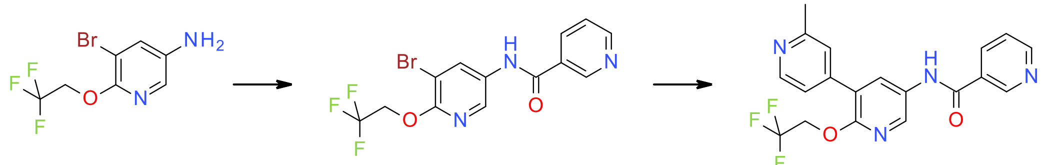

**14** **20** **Cpd R**

***N*-(5-Bromo-6-(2,2,2-trifluoroethoxy)-3-pyridinyl)-3-pyridinecarboxamide (20)**

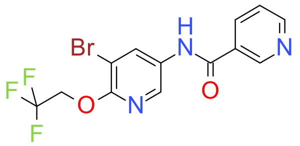

Nicotinoyl chloride hydrochloride (4.73 g, 26.6 mmol) was added to a solution of 5-bromo-6-(2,2,2-trifluoroethoxy)pyridin-3-amine (**14**, 6 g, 22.1 mmol) in dichloromethane (60 ml) and the mixture was cooled to 0°C. To the well stirred, dark solution was added dropwise during ca 20 min sodium hydroxide solution (30 ml, 1 N, 30.0 mmol) and the mixture was stirred at 0° C for another 30 min. A second batch nicotinoyl chloride hydrochloride (2 g, 11.2 mmol) was added dropwise, followed by a second batch of sodium hydroxide solution (15 mL, 15 mmol) and the mixture was stirred at 0 °C for 30 min again. To the resulting slurry nicotinoyl chloride hydrochloride (1 g, 5.62 mmol) was added and the mixture was stirred at 0°C for 1 h. The phases were separated, and the organic phase was purified by chromatography on silica gel to afford the title compound as off-white solid (6.5 g, 78.1 %). $^{1}$H NMR (DMSO-d6, 600 MHz) $\delta$ 10.68 (m, 1H), 9.12 (dd, $J$ = 2.2, 0.8 Hz, 1H), 8.79 (dd, $J$ = 4.8, 1.7 Hz, 1H), 8.55 (d, $J$ = 2.3 Hz, 1H), 8.52 (d, $J$ = 2.3 Hz, 1H), 8.31 (m, 1H), 7.60 (m, 1H), 5.06 (d, $J$ = 9.0 Hz, 2H). $^{13}$C NMR (151 MHz, DMSO-d6): $\delta$ 164.2, 153.5, 152.5, 148.7, 137.2, 135.5, 135.2, 131.9, 129.7, 123.6, 123.9 (q, $J$ = 278.0 Hz), 104.8, 62.4 (q, $J$ = 34.7 Hz). HRMS calculated for $C_{13}H_9BrF_3N_3O_2$ [M+H]$^+$ 375.9908, found 375.9902.

**$^{1}$H NMR of compound 20**

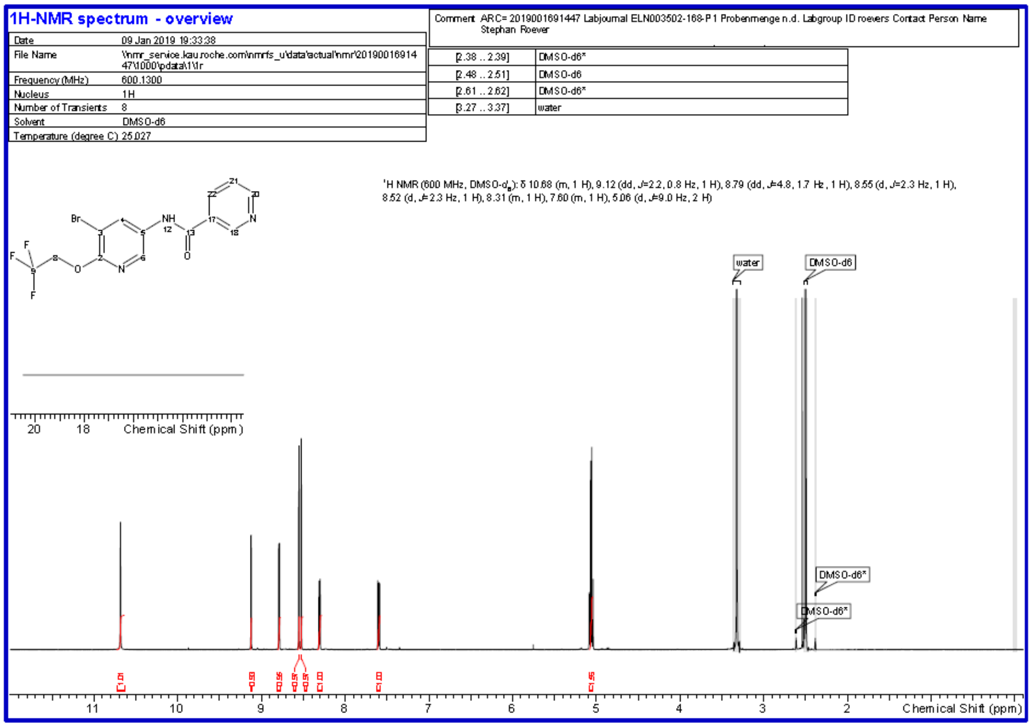


## $^{13}$C NMR of compound 20

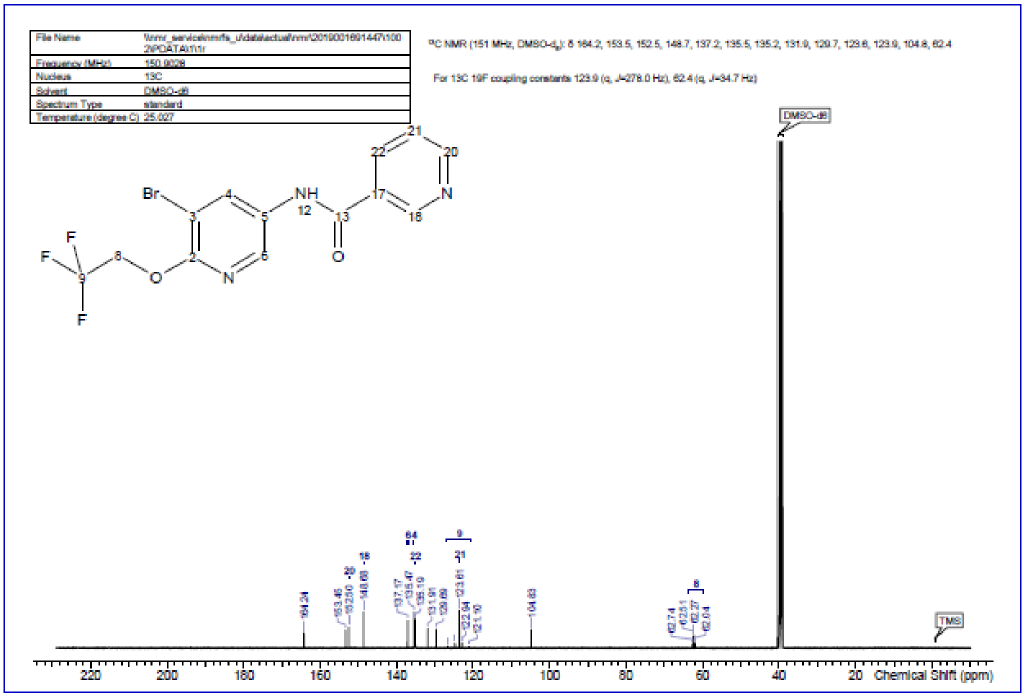

**N-[5-(2-methyl-4-pyridyl)-6-(2,2,2-trifluoroethoxy)-3-pyridyl]pyridine-3-carboxamide (Cpd R)**

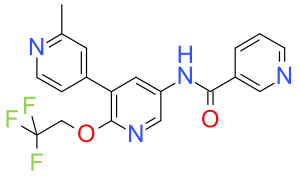

2-Methylpyridine-4-boronic acid (364 mg, 2.66 mmol), [1,1'-bis(diphenylphosphino)-ferrocene]dichloropalladium(II) x $CH_2Cl_2$ complex (1:1) (29 mg, 0.04 mmol) and sodium carbonate solution (1.33 mL, 2M, 2.66 mmol) was added to a solution of *N*-(5-bromo-6-(2,2,2-trifluoroethoxy)-3-pyridinyl)-3-pyridinecarboxamide (**20**, 500mg, 1.33 mmol) in toluene (20 mL) and stirred for 3 h at 90°C and at room temperature over the weekend. Water (15 mL) was added and organic material was extracted into ethyl acetate. After drying with magnesium sulfate and evaporation of solvent, the greenish-white solid (0.67 g) was purified by preparative HPLC to afford the title compound as white solid (52 mg, 10%). $^{1}$H NMR (DMSO-d6, 600 MHz) δ 10.72 (s, 1H), 9.16 (dd, *J* = 1.7, 0.7 Hz, 1H), 8.79 (dd, *J* = 4.7, 1.6 Hz, 1H), 8.65 (d, *J* = 2.5 Hz, 1H), 8.56 (d, *J* = 5.2 Hz, 1H), 8.36 (d, *J* = 2.5 Hz, 1H), 8.34 (m, 1H), 7.61 (m, 1H), 7.49 (m, 1H), 7.43 (dd, *J* = 5.2, 1.1 Hz, 1H), 5.07 (q, *J* = 9.1 Hz, 2H), 2.54 (s, 3H). $^{13}$C NMR (151 MHz, DMSO-d6): δ 164.2, 158.1, 154.0, 152.4, 148.9, 148.9, 148.7, 138.6, 135.4, 132.6, 131.7, 129.8, 123.6, 124.1 (q, *J* = 278.1 Hz), 122.7, 120.7, 120.6, 61.8(q, *J* =3 4.7 Hz), 24.0. HRMS calculated for $C_{19}H_{15}F_3N_4O_2$ $[M+H]^+$ 389.1225, found 389.1223.

**$^{1}$H NMR of compound R**

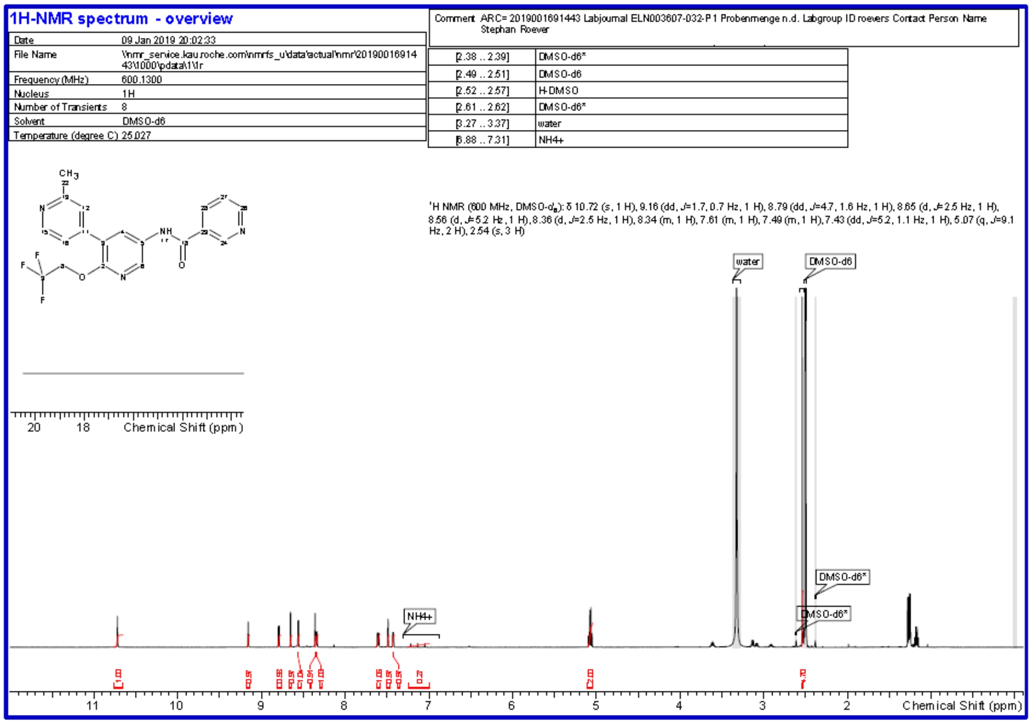


## $^{13}$C NMR of compound R

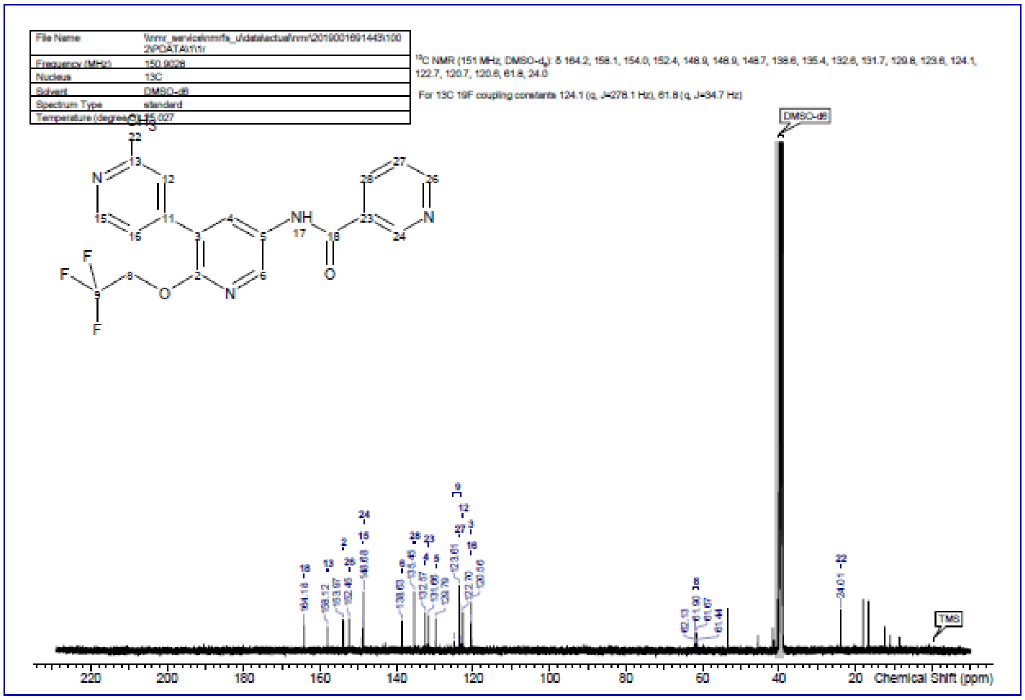

**Synthesis of *N*-[5-(5-methyl-3-pyridyl)-6-(2,2,2-trifluoroethoxy)-3-pyridyl]pyridine-3-carboxamide (Cpd S)**

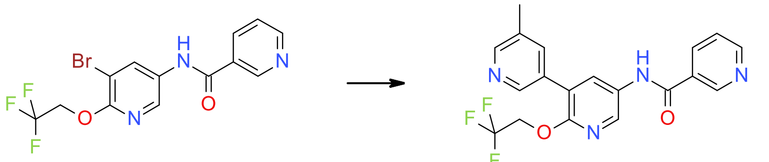

20 Cpd S

***N*-(5-Bromo-6-(2,2,2-trifluoroethoxy)-3-pyridinyl)-3-pyridinecarboxamide (20)**

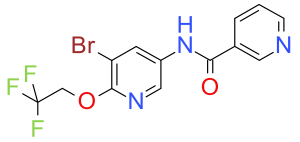

See above

***N*-[5-(5-methyl-3-pyridyl)-6-(2,2,2-trifluoroethoxy)-3-pyridyl]pyridine-3-carboxamide (Cpd S)**

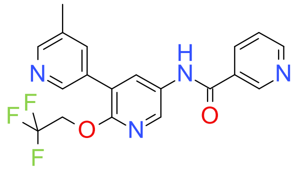

5-Methylpyridine-3-boronic acid (364 mg, 2.66 mmol), [1,1'-bis(diphenylphosphino)ferrocene]-dichloropalladium(II) x $CH_2Cl_2$ complex (1:1) (29 mg, 0.04 mmol) and sodium carbonate solution (1.33 mL, 2M, 2.66 mmol) was added to a solution of *N*-(5-bromo-6-(2,2,2-trifluoroethoxy)-3-pyridinyl)-3-pyridinecarboxamide (**20**, 500mg, 1.33 mmol) in toluene (20 mL) and stirred for 3 h at 90°C and at room temperature over the weekend. Water (15 mL) was added and organic material was extracted into ethyl acetate. After drying with magnesium sulfate and evaporation of solvent, the greenish-white solid (0.81 g) was purified by preparative HPLC to afford the title compound as white solid (34 mg, 6.6%). $^{1}$H NMR ($CDCl_3$, 600 MHz) $\delta$ 9.1-9.2 (m, 1H), 8.81 (dd, 1H, *J* = 1.5, 4.7 Hz), 8.62 (d, 1H, *J* = 1.7 Hz), 8.0-8.5 (m, 5H), 7.78 (s, 1H), 7.48 (dd, 1H, *J* = 4.8, 7.8 Hz), 4.83 (q, 2H, *J* = 8.5 Hz), 2.40 (s, 3H). $^{13}$C NMR (151 MHz, DMSO-d6): $\delta$ 164.1, 154.1, 152.4, 149.3, 148.7, 146.4, 138.0, 136.6, 135.4, 132.7, 132.6, 131.6, 130.4, 129.8, 123.6,

124.1 (q, $J$ = 277.6 Hz), 120.2, 61.8 (q, $J$ = 34.5 Hz), 17.8. HRMS calculated for $C_{19}H_{15}F_3N_4O_2$ $[M+H]^+$ 389.1225, found 389.1226.

## $^{1}$H NMR of compound S

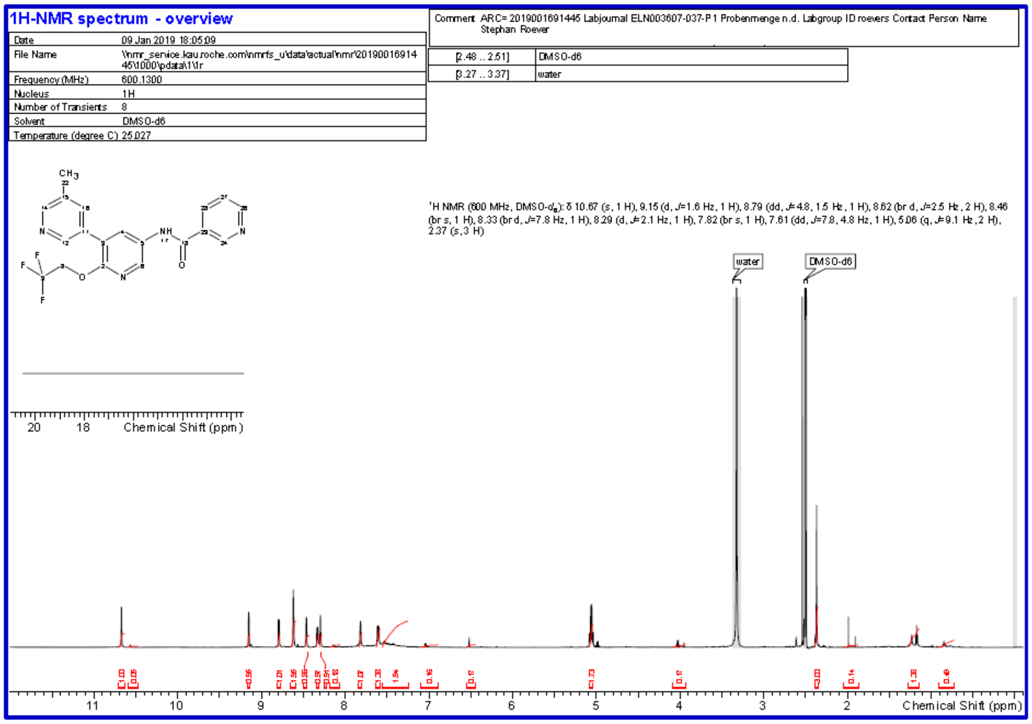


## $^{13}$C NMR of compound S

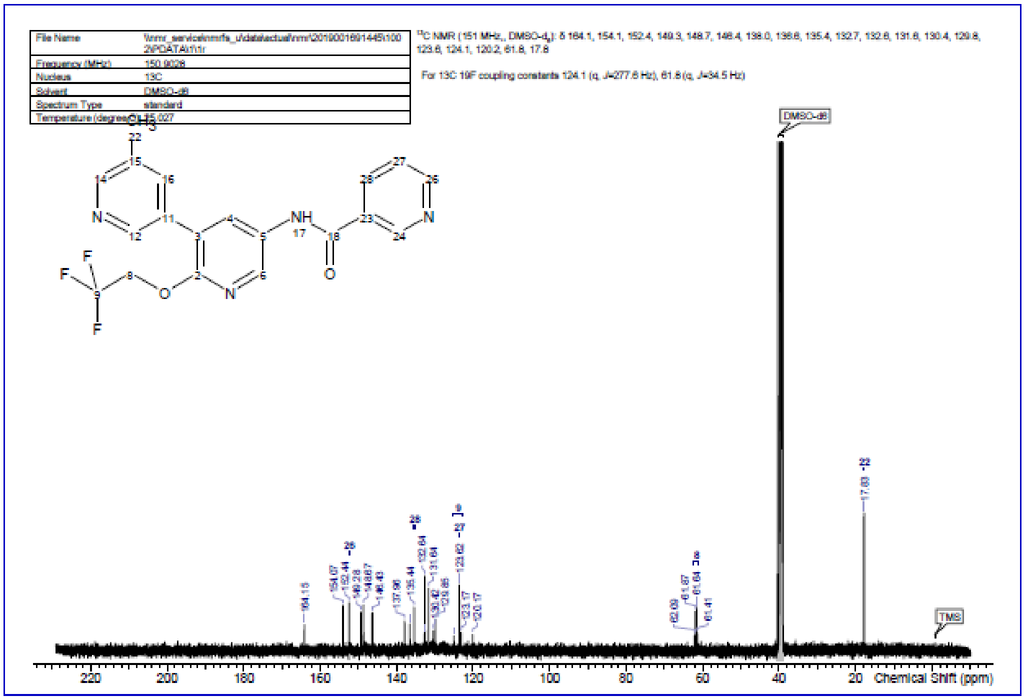


**Synthesis of 6-(3,4-dichlorophenyl)-*N*-[(1*R*,2*R*)-2-hydroxycyclohexyl]-5-(2,2,2-trifluoroethoxy-2-pyridinecarboxamide (Cpd A)**

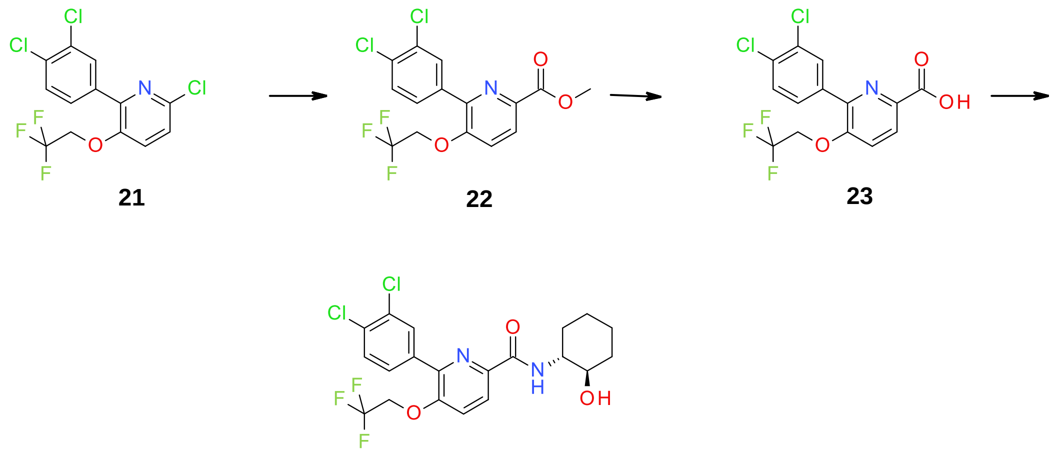


**6-Chloro-2-(3,4-dichlorophenyl)-3-(2,2,2-trifluoroethoxy)-pyridine**

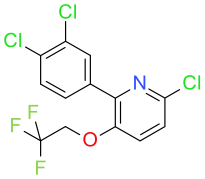

Commercial

**6-(3,4-Dichlorophenyl)-5-(2,2,2-trifluoroethoxy)-2-pyridinecarboxylic acid methyl ester (22)**

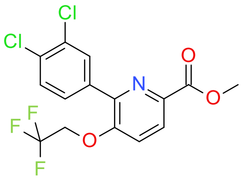

Triethylamine (7.47 mL, 53.6 mmol) and [1,1'-bis(diphenylphosphino)ferrocene]-dichloropalladium(II) x $CH_2Cl_2$ complex (1:1) (1.64 g, 2.01 mmol) was added to a solution of 6-chloro-2-(3,4-dichlorophenyl)-3-(2,2,2-trifluoroethoxy)-pyridine (**21**, 9.55 g, 18 mmol) in methanol (100 mL) and carbonylated in a CO atmosphere (70 bar, 110°C, 20 h). The reaction mixture was concentrated in vacuo and the residue was partitioned between water and ethyl acetate. Organic layers were washed with brine, dried over $Na_2SO_4$ and concentrated in vacuo. Purification by flash chromatography (silica, n-heptane, ethyl acetate gradient) afforded the title compound as white solid (5.65 g mg, 79%). $^1$H NMR (600 MHz, DMSO-d6): δ 8.15 (d, $J$ = 8.7

Hz, 1H), 8.13 (d, $J$ = 2.1 Hz, 1H), 7.90 (dd, $J$ = 8.4, 2.1 Hz, 1H), 7.85 (d, $J$ = 8.7 Hz, 1H), 7.80 (d, $J$ = 8.4 Hz, 1H), 5.05 (q, $J$ = 8.7 Hz, 2H), 3.93 – 3.83 (m, 3H). $^{13}$C NMR (151 MHz, DMSO-$d_6$): δ 164.4, 153.5, 143.8, 140.4, 136.4, 131.7, 130.8, 130.8, 130.4, 129.2, 126.2, 123.7 (q, $J$ = 277.9 Hz), 121.8, 65.0 (q, $J$ = 34.8 Hz), 52.4. HRMS calculated for $C_{15}H_{10}Cl_2F_3NO_3$ $[M+H]^+$ 380.0068, found 380.0065.

**$^1$H NMR of compound 22**

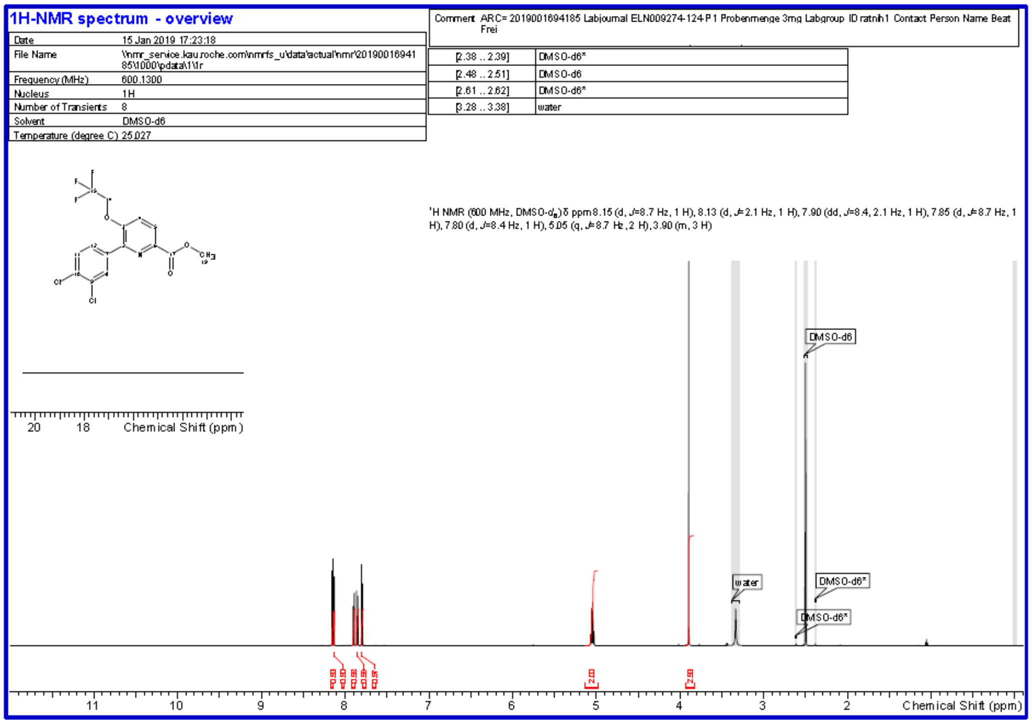


**$^{13}$C NMR of compound 22**

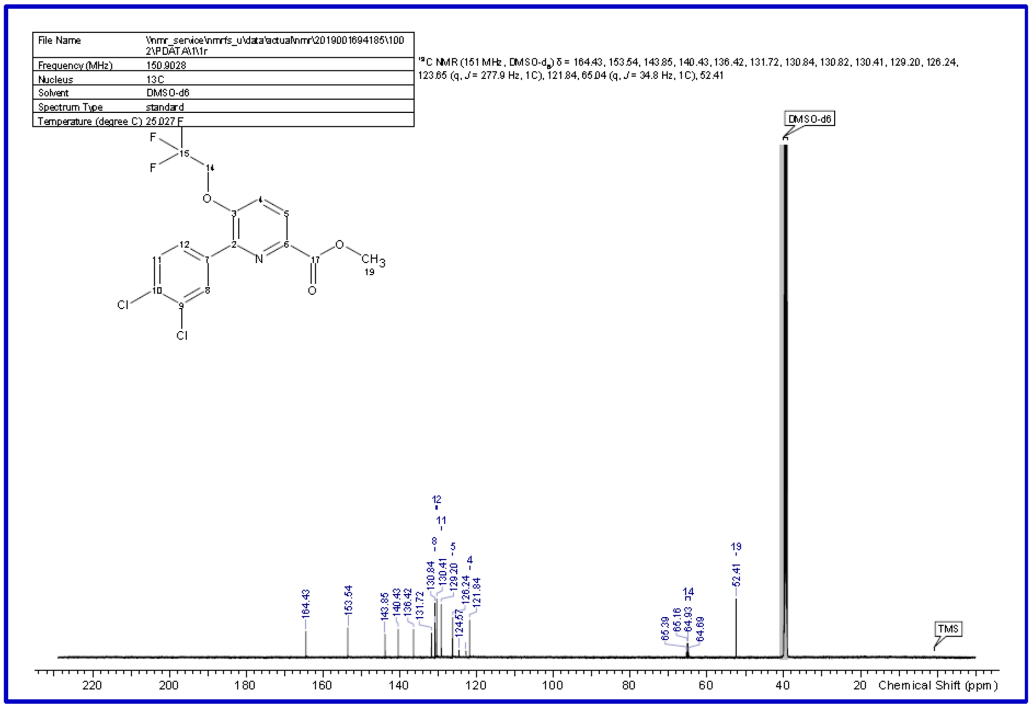
File Name
\\nmr_service\nmrfs_u\data\actual\nmr\2019001694185\1002\PDATA\1\1r
Frequency (MHz) 150.9028
Nucleus 13C
Solvent DMSO-d6
Spectrum Type standard
Temperature (degree C) 25.027
¹³C NMR (151 MHz, DMSO-d6) δ = 164.43, 153.54, 143.85, 140.43, 136.42, 131.72, 130.84, 130.82, 130.41, 129.20, 126.24, 123.65 (q, J = 277.9 Hz, 1C), 121.84, 65.04 (q, J = 34.8 Hz, 1C), 52.41
DMSO-d6
TMS
Chemical Shift (ppm)

**6-(3,4-Dichlorophenyl)-5-(2,2,2-trifluoroethoxy)-2-pyridinecarboxylic acid (23)**

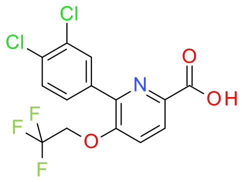

6-(3,4-Dichlorophenyl)-5-(2,2,2-trifluoroethoxy)-2-pyridinecarboxylic acid methyl ester (**22**, 543 mg, 1.43 mmol) was combined with THF (10 mL) to give a colorless solution. Water (5 mL) and LiOH (68.4 mg, 2.86 mmol) was added and the reaction mixture was stirred 2 h at 40°C.
The reaction mixture was poured into 50 mL saturated $NH_4Cl$ solution and partitioned into ethyl acetate. Organic layers were combined, dried over $Na_2SO_4$ and concentrated in vacuo to afford the title compound without further purification as white solid (540 mg, quant.). The compound was used in the next step without analysis of the sample.

**6-(3,4-Dichlorophenyl)-*N*-[(1*R*,2*R*)-2-hydroxycyclohexyl]-5-(2,2,2-trifluoroethoxy-2-pyridinecarboxamide (Cpd A)**

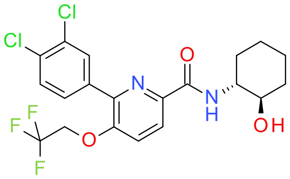


TBTU (424 mg, 1.32 mmol), *N*,*N*-diisopropylethylamine (768 µl, 4.4 mmol) and (1*R*,2*R*)-2-aminocyclohexanol hydrochloride (160 mg, 1.06 mmol) was added to a solution of 6-(3,4-dichlorophenyl)-5-(2,2,2-trifluoroethoxy)-2-pyridinecarboxylic acid (**23**, 322 mg, 879 µmol) in DMF (12 mL). The mixture was stirred for 3 h at room temperature and partitioned between water and $CH_2Cl_2$. Organic phases were combined, dried with $Na_2SO_4$ and concentrated. Purification by flash chromatography (basic alumina, n-heptane, ethyl acetate 2:8) afforded the title compound after precipitation as white solid (295 mg, 72%). $^{1}H$ NMR (600 MHz, DMSO-$d_6$): δ 8.23 (d, *J* = 2.0 Hz, 1H), 8.22 (d, *J* = 8.2 Hz, 1H), 8.08 (d, *J* = 8.6 Hz, 1H), 8.02 (dd, *J* = 8.5, 2.1 Hz, 1H), 7.86 (d, *J* = 8.6 Hz, 1H), 7.79 (d, *J* = 8.5 Hz, 1H), 5.08 – 4.95 (m, 2H), 4.76 – 4.61 (m, 1H), 3.65 – 3.57 (m, 1H), 3.52 – 3.47 (m, 1H), 1.94 – 1.86 (m, 2H), 1.70 – 1.59 (m, 2H), 1.40 – 1.31 (m, 1H), 1.28 – 1.21 (m, 3H). $^{13}C$ NMR (151 MHz, DMSO-d6): δ 162.9, 152.9, 143.8, 142.4, 136.4, 131.6, 131.1, 130.8, 130.3, 129.5, 123.0, 123.7 (q, *J* = 277.6 Hz), 122.4, 70.8, 65.1 (q, *J* = 34.6 Hz), 55.2, 34.6, 31.0, 24.4, 24.1. HRMS calculated for $C_{20}H_{19}Cl_2F_3N_2O_3$ $[M+H]^+$ 463.0803, found 463.0802.

## $^{1}H$ NMR of compound A

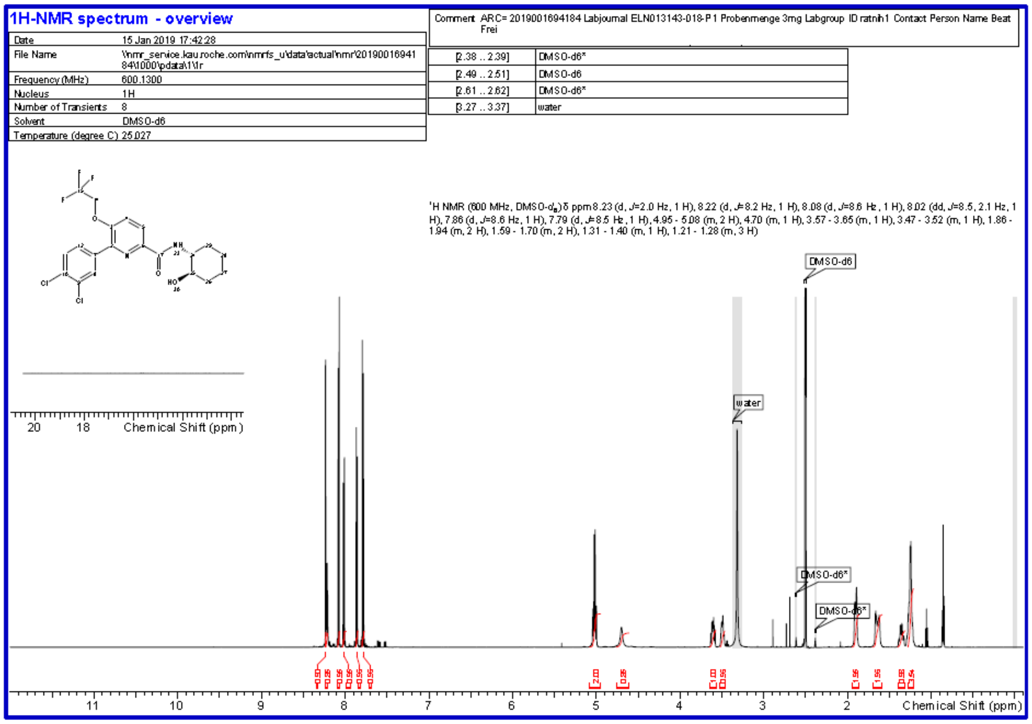


## $^{13}C$ NMR of compound A

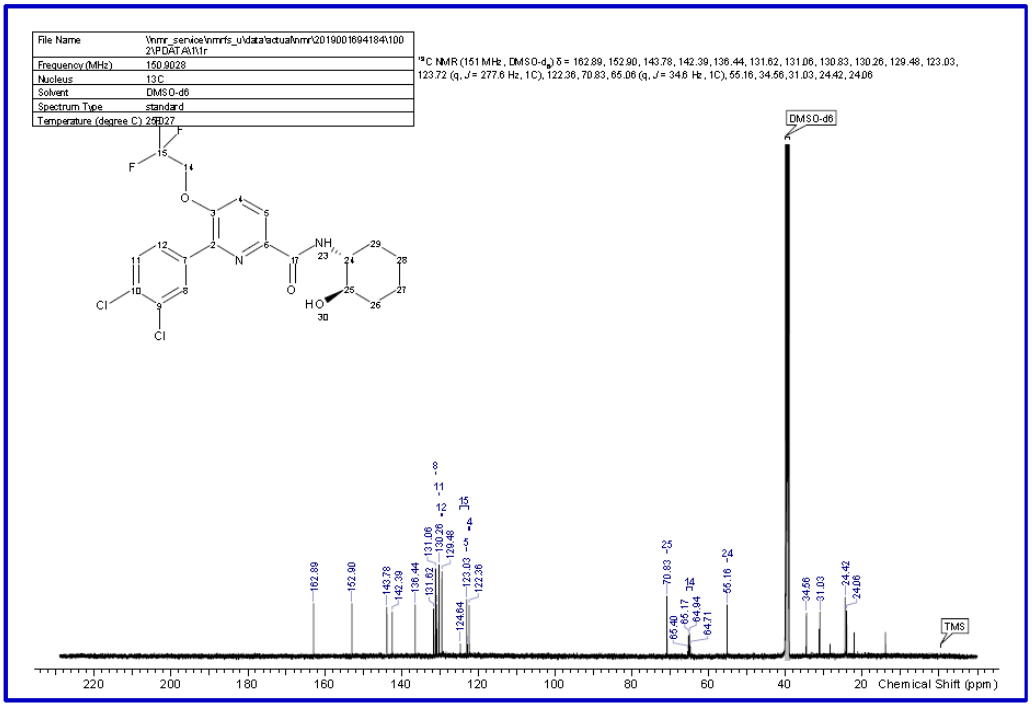

**Reference**

1. Röver, S.; Andjelkovic, M.; Bénardeau, A.; Chaput, E.; Guba, W.; Hebeisen, P.; Mohr, S.; Nettekoven, M.; Obst, U.; Richter, W. F.; Ullmer, C.; Waldmeier, P. and Wright M. B. **6-Alkoxy-5-aryl-3-pyridinecarboxamides, a New Series of Bioavailable Cannabinoid Receptor Type 1 (CB1) Antagonists Including Peripherally Selective Compounds** *J. Med. Chem.* 2013, 56, 9874−9896. dx.doi.org/10.1021/jm4010708

2. Cheng, Y.; Albrecht, B. K.; Brown, J.; Buchanan, J- L.; Buckner, W. H.; DiMauro, E. F.; Emkey, R.; Fremeau, R. T.; Harmange, J.-C.; Hoffman, B. J.; et al. **Discovery and Optimization of a Novel Series of N-Arylamide Oxadiazoles as Potent, Highly Selective and Orally Bioavailable Cannabinoid Receptor 2 (CB2) Agonists** *J. Med. Chem.* 2008, 51, 5019−5034. dx.doi.org/10.1021/jm800463f

3. Katoch-Rouse, R.; Pavlova, O. A.; Caulder, T.; Hoffman, A. F.; Mukhin, A. G.; Horti, A. G. **Synthesis, Structure−Activity Relationship, and Evaluation of SR141716 Analogues: Development of Central Cannabinoid Receptor Ligands with Lower Lipophilicity** *J. Med. Chem.* 2003, 46, 642−645. dx.doi.org/10.1021/jm020157x

4. Liu, Q.; Tor, Y. **Simple Conversion of Aromatic Amines into Azides** *Org. Lett.* 2003, 5, 2571-2572. dx.doi.org/10.1021/ol034919+

5. Garcia, M.; Serra, A,; Rubiralta, M.; Diez, A.; Segarra, V.; Lozoya, E.; Ryder, H.; Palacios, J. M. **Efficient method for the preparation of (S)-5-hydroxynorvaline** *Tetrahedron Asymmetry*, 2000, 991. dx.doi.org/10.1016/S0957-4166(00)00020-3

**Table 1. Compound names and structures**

| | |
|---|---|
| **Cpd H** | 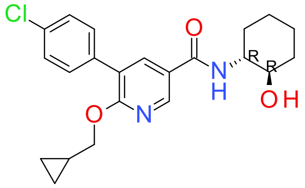 |
| **Cpd J** | 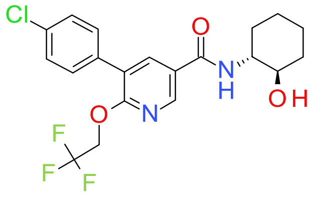 |
| **Rimonabant** | 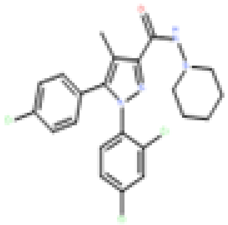 |
| **Cpd N** | 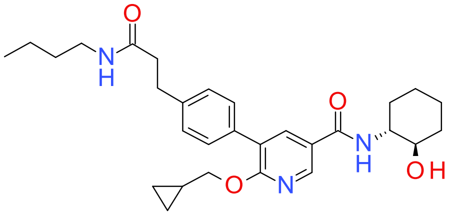 |
| **Cpd K** | 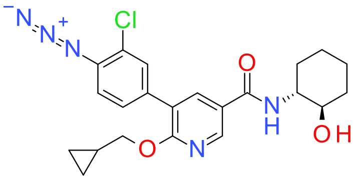 |
| **2,4-$^3H_2$-K** | 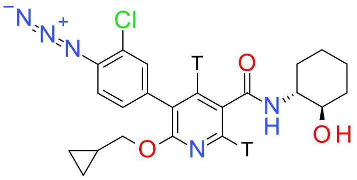 |
| **Cpd G** | 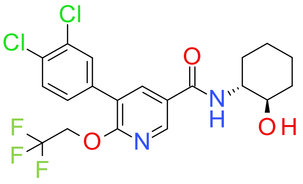 |

| | |
|---|---|
| **Cpd L** | 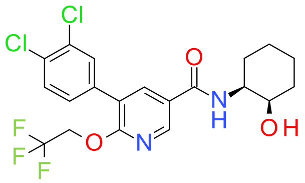 |
| **Cpd P** | 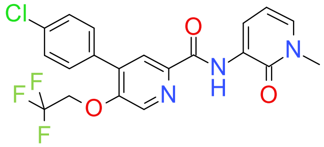 |
| **Cpd M** | 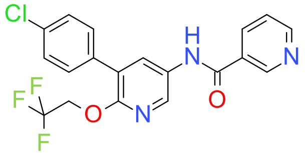 |
| **Cpd Q** | 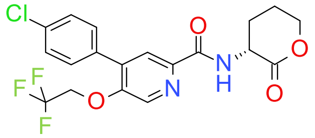 |
| **Cpd R** | 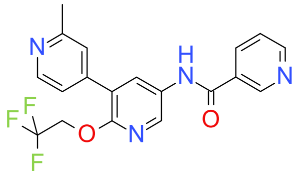 |
| **Cpd S** | 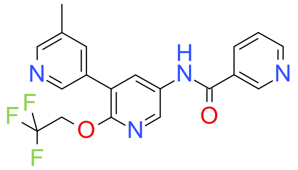 |
| **Cpd A** | 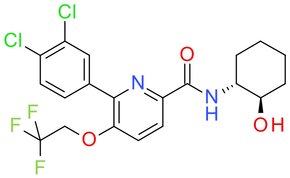 |
| **Cpd C** | 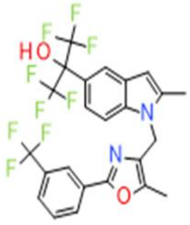 |
| **T091317** | 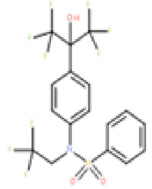 |
