# Supplementary material for: Compounds targeting OSBPL7 increase ABCA1-dependent cholesterol efflux preserving kidney function in two models of kidney disease
Source: Nat Commun. 2021 Aug 2;12:4662. doi: 10.1038/s41467-021-24890-3 (PMC8329197; doi:10.1038/s41467-021-24890-3)
Supplement: Supplementary file 4 — Reporting Summary [file 41467_2021_24890_MOESM4_ESM.pdf]

nature research

Corresponding author(s): Alessia Fornoni

# Reporting Summary

Nature Research wishes to improve the reproducibility of the work that we publish. This form provides structure for consistency and transparency in reporting. For further information on Nature Research policies, see Authors & Referees and the Editorial Policy Checklist.

## Statistical parameters

When statistical analyses are reported, confirm that the following items are present in the relevant location (e.g. figure legend, table legend, main text, or Methods section).

| n/a | Confirmed | |
|---|---|---|
| ☐ | ☒ | The exact sample size ($n$) for each experimental group/condition, given as a discrete number and unit of measurement |
| ☐ | ☒ | An indication of whether measurements were taken from distinct samples or whether the same sample was measured repeatedly |
| ☐ | ☒ | The statistical test(s) used AND whether they are one- or two-sided<br>*Only common tests should be described solely by name; describe more complex techniques in the Methods section.* |
| ☒ | ☐ | A description of all covariates tested |
| ☐ | ☒ | A description of any assumptions or corrections, such as tests of normality and adjustment for multiple comparisons |
| ☐ | ☒ | A full description of the statistics including central tendency (e.g. means) or other basic estimates (e.g. regression coefficient) AND variation (e.g. standard deviation) or associated estimates of uncertainty (e.g. confidence intervals) |
| ☐ | ☒ | For null hypothesis testing, the test statistic (e.g. $F$, $t$, $r$) with confidence intervals, effect sizes, degrees of freedom and $P$ value noted<br>*Give P values as exact values whenever suitable.* |
| ☒ | ☐ | For Bayesian analysis, information on the choice of priors and Markov chain Monte Carlo settings |
| ☒ | ☐ | For hierarchical and complex designs, identification of the appropriate level for tests and full reporting of outcomes |
| ☒ | ☐ | Estimates of effect sizes (e.g. Cohen's $d$, Pearson's $r$), indicating how they were calculated |
| ☐ | ☒ | Clearly defined error bars<br>*State explicitly what error bars represent (e.g. SD, SE, CI)* |

*Our web collection on statistics for biologists may be useful.*

## Software and code

Policy information about availability of computer code

| | |
|---|---|
| Data collection | - RTPCR signal captured using StepOne Plus Real Time PCR System, Applied Biosystems v2.3<br>- Western blot chemiluminescence signals were acquired using either X-ray films or using Azure Biosystems C600 with capture software version 1.9.7.082<br>- Microscope used to capture PAS-stained slides: Olympus BX41, equipped with camera Idea 5MP Color mosaic model 28.2 and software SPOT Basic version 5.1.23<br>- Microscope used to capture IF (ABCA1-WT-1): FluoView FV1000 Confocal Microscope (Olympus) with a 63x oil objective lens using fluview software version 4.3b (Olympus).<br>- Microscope used to capture IF (OSBPL7-SYNPO): Leica AF 7000 (software LAS AF, ver 2.6.0.7266)<br>- Reading for all lipid assays: Spectramax i3X, Molecular Devices, (software SoftMax Pro7 Standard)<br>No custom algorithms or software were used. |
| Data analysis | Statistics and graphs: All data were processed with Graph Prizm version 6.0<br>RT-PCR signals: captured and analyzed using StepOne Plus Real Time PCR System, Applied Biosystems v2.3<br>Western blot images adjusted with Image J Software, Java 1.8. 0_45 [64-bit]. Band intensity quantified using same software.<br>No custom algorithms or software were used. |

For manuscripts utilizing custom algorithms or software that are central to the research but not yet described in published literature, software must be made available to editors/reviewers upon request. We strongly encourage code deposition in a community repository (e.g. GitHub). See the Nature Research guidelines for submitting code & software for further information.

## Data

Policy information about availability of data

All manuscripts must include a data availability statement. This statement should provide the following information, where applicable:

- Accession codes, unique identifiers, or web links for publicly available datasets
- A list of figures that have associated raw data
- A description of any restrictions on data availability

The authors declare that the data supporting the findings of this study are available within the paper and its supplementary information files (Source Data file and Supplementary information file).
List of figures with associated raw data supplied either in Supplementary or Source Data files:
Figure 1
Figure 2
Figure 3
Figure 4
Figure 5
Figure 6
Figure 7
Figure 8
Fig. S8
Fig. S9
Fig. S10
Fig. S11
Fig. S12
Fig. S13
Tables S3 and S4

No restrictions applied for Data availability

# Field-specific reporting

Please select the best fit for your research. If you are not sure, read the appropriate sections before making your selection.

☒ Life sciences ☐ Behavioural & social sciences

For a reference copy of the document with all sections, see nature.com/authors/policies/ReportingSummary-flat.pdf

# Life sciences

## Study design

All studies must disclose on these points even when the disclosure is negative.

| | |
|---|---|
| Sample size | Minimal group sizes for in vitro and in vivo studies were determined via power calculation using the DSS Researcher's Toolkit with an α of 0.05. |
| Data exclusions | No data from from in vitro and in vivo experiments was excluded except for the one corresponding to a mouse that suffered accidental extravasation of Adriamycin (ADR) during IV injection and was euthanized. |
| Replication | In vitro experiments were repeated 3 times. If no inconsistencies were found, the experiments were considered successful, otherwise, the experiments were repeated more times.<br>For in vivo experiments, each animal was considered an independent experiment. All attempts at replication were considered successful as similar results/trends were observed in most samples. In addition, the in vivo experiment where more variation was observed was repeated with a larger set of animals with similar results/trennds even when done at a different time point with a new batch of animals and reagents. were used |
| Randomization | Animals were grouped unblinded, but randomized. Cell culture samples and animal samples were allocated randomly. |
| Blinding | All data derived from in vivo experiments, including pathology, IF study, lipid assays, ACR, BUN, sCre, etc were generated in a blind manner (the person performing the analysis did not know the distribution of animal identifiers (tags) within treatment groups)<br>Some in vitro studies, like Western blots, could not be performed in a blind manner because the investigator need to know the position in gels and plate layout for the treatments performed and there was no other personnel qualified to process the samples. However, cholesterol efflux experiments with THP1 cells were performed in a blind manner in different countries with similar results. |

# Materials & experimental systems

Policy information about availability of materials

| n/a | Involved in the study |
|---|---|
| ☐ | ☒ Unique materials |
| ☐ | ☒ Antibodies |
| ☐ | ☒ Eukaryotic cell lines |
| ☐ | ☒ Research animals |
| ☒ | ☐ Human research participants |

## Unique materials

| | |
|---|---|
| Obtaining unique materials | ABCA1 inducer compounds were synthesized at Roche, but are no longer available. |

## Antibodies

**Antibodies used**

Primary antibodies used for Western blots:
Rabbit anti Na/K-ATPase (#3010), Cell Signaling Technology (MA, USA), diluted 1: 1,000
Rabbit anti-MEK-1/2 (D1A5) (#8727), Cell Signaling Technology (MA, USA), diluted 1: 1,000
Rabbit anti-ERp72 (D70D12, # 5033), Cell Signaling Technology (MA, USA), diluted 1: 1,000
Rabbit anti-V5-Tag (D3H8Q, #13202): Cell Signaling Technology (MA, USA), diluted 1: 1,000
mouse anti-ABCA1 monoclonal anitbody, clone AB.H10. ABCAM Cat# ab18180, diluted 1: 1,000
Rabbit anti-OSBPL7 polyclonal antibody. Sigma-Aldrich, Cat# HPA036076, diluted 1:1,000
mouse anti-GAPDH monoclonal antibody, clone 6C5, Millipore-Sigma, Cat# CB1001) . Diluted 1: 10,000
mouse anti-beta-Actin monoclonal antibody, clone 4C2 (EMD-Millipore-Sigma , Cat# MABT825). Diluted 1:10,000

Secondary antibodies HRP-conjugated used in Western blot were purchased from Promega Corp. (WI, USA) and used in a 1:10,000 dilution.

Antibodies used for immunofluorescence (IF).
I. For mouse kidney sections stained with OSBPL7 and Synaptopodin
Primary antibodies:
- Rabbit polyclonal anti-OSBPL7. Sigma-Aldrich, Cat# HPA036076, Lot# R70903. Diluted 1:50
- Goat anti-Synaptopodin polyclonal antibody (P19). Santa Cruz Biotechnology (TX, USA), Cat# # sc-21537, Lot# F2415. Diluted 1:500

Secondary antibodies:
Goat anti-Rabbit IgG (H+L), Alexa Fluor 568, Invitrogen, Cat# A-11036, Lot #1301874. Diluted 1:500
Donkey anti-Goat IgG (H+L), Alexa Fluor 488, Invitrogen, Cat# A-11055, Lot# 1627966. Diluted 1:800

II. For ABCA1 and WT1 in mouse kidney sections
Primary antibodies:
- Rat anti-ABCA1 monoclonal antibody, clone 3A1.891.3. Novus Biologicals, Cat# NB400-164, Lot# B1. Diluted 1:200
- Rabbit anti-WT1 monoclonal antibody, clone CAN-R9(IHC).-56-2. Abcam, Cat# ab216646, Lot# GR3314729-6. Diluted 1:200

Secondary antibodies:
Goat anti-Rat IgG (H+L), Alexa Fluor 488, Invitrogen, Cat# A11006, Lot# 1928689. Diluted 1:500
Goat anti-Rabbit IgG (H+L), Alexa Fluor 594, Invitrogen (CA, USA), Cat# A11037, Lot# 1981132 . Diluted 1:500.

Capture antibody used for immunoprecipitation:
anti FLAG M2 Affinity Gel. Sigma Aldrich, Cat #A2220-10ml, Lot# SLBH2097V.
Dilution 15 ul (net volume) per 1 ml of clarified cell lysate.

**Validation**

Western Blots:
ABCA1. Mouse antiABCA1 monoclonal antibody (AB.H10). Provider validation statement including relevant citations is available here https://www.abcam.com/abca1-antibody-abh10-ab18180.html.
antibody validated with positive control cell lines (THP1, cells overexpressing ABCA1, compounds that induce protein expression), and negative control (cells that express very little of ABCA1 like HEK293)

- OSBPL7 rabbit polyclonal antibody (Sigma #HPA036076), anti-human reactivity. Provider validation statement including relevant citations is available here https://www.sigmaaldrich.com/catalog/product/sigma/hpa036076?lang=en&region=US. We also validated this Ab by WB in cells transfected with a vector to overexpress this protein or with siRNAs to reduce endogenopus expression.

- Na,K-ATPase: polyclonal anti-rabbit; human, mouse, rat, hamster, simian cross-reactivity. Provider validation statement including relevant citations is available here https://www.cellsignal.com/products/primary-antibodies/na-k-atpase-antibody/3010. Antibodypedia database profile is available here https://www.antibodypedia.com/gene/4542/ATP1A1/antibody/106224/3010.

- MEK1/2 (D1A5): monoclonal anti-rabbit; human, mouse, rat, monkey, D. melanogaster cross-reactivity. Provider validation statement including relevant citations is available here https://www.cellsignal.com/products/primary-antibodies/mek1-2-d1a5-rabbit-mab/8727?site-search-type=Products&N=4294956287&Ntt=mek1%2F2+&fromPage=plp.

- ERp72 (D70D12, # 5033): Rabbit polyclonal antibody anti-human, mouse, rabbit, donkey cross reactivity. Provider validation statement including relevant citations is available here https://www.cellsignal.com/products/primary-antibodies/erp72-d70d12-xp-rabbit-mab/5033.

V5-Tag (D3H8Q) Rabbit mAb #13202: Provider validation statement including over 126 citations is available at https://www.cellsignal.com/products/primary-antibodies/v5-tag-d3h8q-rabbit-mab/13202

GAPDH clone 6C5, Millipore-Sigma, Cat# CB1001. Provider validation statement including citations is available at https://www.emdmillipore.com/US/en/product/Anti-GAPDH-Mouse-mAb-6C5,EMD_BIO-CB1001. More citations at https://www.abcam.com/gapdh-antibody-6c5-loading-control-ab8245.html

Immunofluorescence:
- Rat anti-ABCA1 monoclonal antibody: clone 3A1.891.3., NOVUS, Cat# NB400-164, Lot# B1
Validation for IF in mouse aortas (PMID 19718435ABCA1 ).Provider validation statement including citation of use in immunofluorescence is available at https://www.novusbio.com/products/abca1-antibody-3a18913_nb400-164. This antibody failed recognize human ABCA1 (no IF signal in PFA-fixed and permeabilized THP1 cells, treated or untreated with LXR agonist)

-Goat anti-WT1 monoclonal antibody: Clone CAN-R9(IHC)-56-2, ABCAM, Cat# ab216646, Lot# GR3314729-6. Provider validation in mouse (PMID: 2387491) and in human (PMID: 23042785). Statement including citation is available at: https://www.citeab.com/antibodies/4636807-ab216646-anti-wilms-tumor-protein-antibody-can-r9-i

-Goat anti-Synaptopodin polyclonal antibody (P19), Santa Cruz, Cat# sc-21537, Lot# F2415. Validated in human podocytes (DOI:10.1093/ndt/gfs005). Provider's validation statement including citation is available at: https://www.scbt.com/p/synaptopodin-antibody-p-19

In vivo studies were consistent in two experiments (ADR model)

## Eukaryotic cell lines

Policy information about cell lines

| | |
|---|---|
| Cell line source(s) | Normal human podocytes (gift from Dr. Jochen Reiser, Rush University, Chicago).<br>Normal and Tangier fibroblasts were provided by Prof. Gerhard Schmitz , Institute of Clinical Chemistry and Laboratory Medicine, Regensburg, Germany.<br>THP1 TIB-202 were purchased from ATCC, all studies were done with passages up to 6.<br>HEK293, CaCO and HEPG2 also from ATCC |
| Authentication | Immortalized human podocytes are regularly checked for podocyte like morphology by phalloidin staining and podocyte marker protein expression such as synaptopodin by Western blot.<br>THP1 TIB-202 purchased from ATCC. We did not perform any authentication test and lost the Lot # from the original vial that would allow us to track authentication from ATCC. However, cell morphology, (size and shape), ability to be activated by PMA and response in cholesterol efflux assays was consistent with published literature. |
| Mycoplasma contamination | All cell lines used tested were negative for mycoplasma contamination by PCR |
| Commonly misidentified lines (See ICLAC register) | No commonly misidentified cell lines were used in the current study. |

## Research animals

Policy information about studies involving animals; ARRIVE guidelines recommended for reporting animal research

Animals/animal-derived materials

Animals were housed in a pathogen-free animal facility of the Division of Veterinary Resources, University of Miami, Miller School of Medicine on 12 h/12 h light/dark cycles under controlled temperature (18-23 C) and humidity (40-60%). Mice were provided a standard 18% protein rodent chow diet and water ad libitum. All animal studies have complied with all relevant ethical regulations and were performed in accordance with the National Institute of Health Guidelines. The study protocol was approved by the Animal Care and Use Committee of the University of Miami, Miller School of Medicine and was conducted accordingly

BALB/cJ (cat #000651) female mice 6 weeks of age, were purchased from the Jackson Laboratory (USA).

J29-Col4a3tm1Dec/J mice (cat # 002908) male and females heterozygous mice, 4 w of age were purchased from Jackson Laboratories. Mice were breeded to generate the Col4a3tm1Dec homozygous population used in the experimental groups. All mice were authenticated and identified by established genotyping methods using standard PCR on tail biopsies.

# Method-specific reporting

| n/a | Involved in the study |
|---|---|
| ☒ | ☐ ChIP-seq |
| ☒ | ☐ Flow cytometry |
| ☒ | ☐ Magnetic resonance imaging |
